# The identification of new cytosolic glutamine synthetase and asparagine synthetase genes in barley (Hordeum vulgare L.), and their expression during leaf senescence

**DOI:** 10.1093/jxb/erv003

**Published:** 2015-02-19

**Authors:** Liliana Avila-Ospina, Anne Marmagne, Joël Talbotec, Karin Krupinska, Céline Masclaux-Daubresse

**Affiliations:** ^1^INRA, UMR1318, Institut Jean-Pierre Bourgin, RD10, F-78000 Versailles, France; ^2^AgroParisTech, Institut Jean-Pierre Bourgin, RD10, F-78000 Versailles, France; ^3^Institute of Botany, Christian-Albrechts-University of Kiel, Olshausenstraße 40, D-24098 Kiel, Germany

**Keywords:** Asparagine synthetase, dark treatment, endoprotease, glutamine synthetase, nitrate.

## Abstract

Glutamine synthetase and asparagine synthetase are two master enzymes involved in ammonium assimilation in plants. Their roles in nitrogen remobilization and nitrogen use efficiency have been proposed. In this report, the genes coding for the cytosolic glutamine synthetases (*HvGS1*) and asparagine synthetases (*HvASN*) in barley were identified. In addition to the three *HvGS1* and two *HvASN* sequences previously reported, two prokaryotic-like *HvGS1* and three *HvASN* cDNA sequences were identified. Gene structures were then characterized, obtaining full genomic sequences. The response of the five *HvGS1* and five *HvASN* genes to leaf senescence was then studied. Developmental senescence was studied using primary and flag leaves. Dark-exposure or low-nitrate conditions were also used to trigger stress-induced senescence. Well-known senescence markers such as the chlorophyll and Rubisco contents were monitored in order to characterize senescence levels in the different leaves. The three eukaryotic-like *HvGS1_1*, *HvGS1_2*, and *HvGS1_3* sequences showed the typical senescence-induced reduction in gene expression described in many plant species. By contrast, the two prokaryotic-like *HvGS1_4* and *HvGS1_5* sequences were repressed by leaf senescence, similar to the *HvGS2* gene, which encodes the chloroplast glutamine synthetase isoenzyme. There was a greater contrast in the responses of the five *HvASN* and this suggested that these genes are needed for N remobilization in senescing leaves only when plants are well fertilized with nitrate. Responses of the *HvASN* sequences to dark-induced senescence showed that there are two categories of asparagine synthetases, one induced in the dark and the other repressed by the same conditions.

## Highlight

The five *HvGS1* and five *HvASN* genes identified in the barley genome are differentially expressed in barley leaves depending on senescence, nitrogen regime, and dark exposure.

## Introduction

Barley (*Hordeum vulgare* L.) is a major grain cereal grown widely and used as animal fodder and for fermentation to make beer or whisky. Cereals are of primary importance to ensure food security and nitrogen use efficiency is a key target for improvement. Barley is cultivated across substantially less area than maize, rice or wheat. Its genome (2*n*=2*x*=14) is smaller and simpler compared with wheat. The fact that a whole-genome shotgun assembly and an integrated physical map are available (Mayer *et al.*, 2012), makes barley a useful model system for the study of temperate cereal crops, especially wheat whose genome is much more complex.

Senescence is the last developmental stage before leaves die and a very important physiological process for the plant. The numerous molecular and biological processes that contribute to senescence are indeed essential for the recycling and remobilization of mineral nutrients and nitrogen-containing molecules from the leaves to the rest of the plant (Himelblau and Amasino, 2001; Diaz *et al.*, 2008). During leaf senescence, proteins and nucleic acids are used as nutrient sources for the building of new organs and for grain-filling in cereals (Kichey *et al.*, 2007; Distelfeld *et al.*, 2012; Fischer, 2012; Gregersen *et al.*, 2013).

The molecular mechanisms involved in nitrogen remobilization have been studied for a long time, in several plant species and using both reverse and forward genetics (see Masclaux-Daubresse *et al.*, 2010). Nitrogen availability has a strong effect on leaf senescence and on nitrogen remobilization efficiency (Lemaître *et al.*, 2008). The main source of nitrogen for remobilization is chloroplasts. The enzymes suspected of managing nitrogen during leaf senescence have been identified (Buchanan-Wollaston, 1997). Endopeptidase activities working with acidic pH optima in the vacuole (Martinez *et al.*, 2008) and the autophagy pathway (Ishida *et al.*, 2008; Michaeli *et al.*, 2014) are the most probable mechanisms involved in chloroplast protein degradation (Guiboileau *et al.*, 2013). Protein degradation releases a large variety of amino acids, however, it seems that not all of them can be mobilized and efficiently loaded in the phloem saps (Tegeder, 2014). Glutamine and asparagine are considered as the main amino acids involved in nitrogen translocation in the phloem saps of many plant species (Masclaux-Daubresse *et al.*, 2008; Taylor *et al.*, 2012). Watanabe *et al.* (2013) have conducted a large profiling of metabolite changes during senescence in *Arabidopsis*. They observed an increase in the Gln/Glu and Asn/Asp ratios, which suggested a more active interconversion of Asp to Asn and of Glu to Gln during leaf senescence. This observation is in agreement with the important role of glutamine and asparagine in senescing leaves for N management. Therefore, their biosynthesis in source leaves is certainly essential for nitrogen remobilization. Glutamine synthetases are responsible for the assimilation and re-assimilation of ammonium in young and old leaves, respectively. While the chloroplastic glutamine synthetase GS2 decreases with leaf ageing, the cytosolic ones (GS1) are induced in the mesophyll of senescing leaves (Brugière *et al.*, 2000; Masclaux *et al.*, 2000; Martin *et al.*, 2006; Diaz *et al.*, 2008; Orsel *et al.*, 2014). The importance of GS1 isoforms in plant productivity has been shown for maize and rice (Tabuchi *et al.*, 2005; Martin *et al.*, 2006; Lothier *et al.*, 2011). Correlation between GS activity and the amount of N remobilized from the shoot to the grain was demonstrated in wheat using cultivars exhibiting contrasting NUE (Kichey *et al.*, 2007) and using a quantitative genetic approach (Habash *et al.*, 2007). However, the role of the GS1 enzyme is complex because numerous isoforms encoded by a multigenic family exist (three genes in rice, five in maize, and five in *Arabidopsis*). These genes are not regulated in a similar manner nor are they localized in the same tissues (Brugière *et al.*, 2000; Ishiyama *et al.*, 2004; Lothier *et al.*, 2011). Among the five GS1 encoding genes (*GLN1-1* to *GLN1-5*) in maize, only *GLN1-4* is up-regulated during senescence (Martin et al., 2005, 2006). The maize *gln1-3*, *gln1-4*, and *gln1-3.gln1-4* mutants isolated by Martin *et al.* (2006) showed a sharp reduction of kernel yield, whereas kernel N (%) was increased. Interestingly the *GLN1-4* locus co-localized with one maize QTL for thousand-kernel weight, and the *GLN1-3* locus co-localized with another two QTLs for thousand-kernel weight and yield. ^15^N tracing experiments also showed that the *GLN1-2* and *GLN1-4* maize loci co-localize with a QTL for N-remobilization (Hirel *et al.*, 2001; Coque *et al.*, 2008).

Besides glutamine synthetase, asparagine synthetase is also able to assimilate ammonium in plants and it might be involved in N remobilization (Masclaux-Daubresse *et al.*, 2006; Gaufichon *et al.*, 2010). Much less is known about asparagine synthetase mutants. However, the regulation of *ASN* genes in several plant species suggests a role in N remobilization during dark periods and leaf senescence (Gaufichon *et al.*, 2010). In sunflower, the expression of the two *AS* genes (*HAS1* and *HAS1.1*) was only detected in senescing leaves when the amount of asparagine increased (Herrera-Rodriguez *et al.*, 2006). In *Arabidopsis* three asparagine synthetase genes (*Asn1*, *Asn2*, and *Asn3*) have been isolated (Lam *et al.*, 2003, and references cited therein). Preliminary results suggested a role in nitrogen remobilization for the *Asn1* gene, which is induced during leaf senescence (Masclaux-Daubresse *et al.*, 2010).

Our study was aimed at characterizing and studying the expression of barley *HvGS1* and *HvASN* genes during leaf senescence and in response to starvation-induced senescence. Not all of the *HvGS1* and *HvASN* genes have been characterized so far and the characterization of two more *HvGS1* and three more *HvASN* genes has been reported here. Expression of all the *HvGS1* and *HvASN* genes has been monitored during developmental and stress-induced senescence. Due to the dichotomy existing between monocot and dicot sequences in the phylogenetic trees of *GS1* and *ASN* genes, it was not possible to determine the barley homologous sequences directly from *Arabidopsis* and vice versa. This study, which permits the comparison of barley and *Arabidopsis GS1* and *ASN* genes on the basis of expression similarities, might facilitate the transfer of knowledge between the *Arabidopsis* model plant and monocot plants.

## Materials and methods

### Plant material and growth conditions

*Hordeum vulgare* L. cultivar Golden Promise was grown in a growth chamber (16/8h day/night photoperiod at 25/17 °C). Seeds were sown on sand and 5-d-old plants were transferred to polyvinyl chloride (PVC) tubes (6cm diameter×45cm units) containing sand as a substrate. Plants were watered eight times per day with a high nitrate (5mM ${\text{NO}}_{3}^{-}$; HN) or low nitrate (0.5mM ${\text{NO}}_{3}^{-}$; LN) nutrient solution (see Supplementary Annex S1 at *JXB* online). Twenty days after sowing (DAS) leaves were harvested individually (L1 to L4 in HN; L1 to L3 in LN; from the bottom to the top leaves). Four independent leaf rank samples (containing 18 leaves each) were harvested between 10.00h and 12.00h, and stored at –80 °C for further experiments. Before harvest, the chlorophyll content in the leaves was estimated using a SPAD (SPAD-502 Chlorophyll meter Konica Minolta, Japan). Three plantings were performed and analyses were undertaken on at least two plant cultures.

Dark-stress experiments were carried out in the same growth chamber on plants watered eight times per day with HN solution. Fourteen DAS, the whole plants were either covered with black boxes taking care that air could pass through (dark stress) or not (control) for 4 d. At the end of the dark stress (time point T1) half of the plants were harvested and leaf ranks collected. The remaining plants were left growing in the normal day/night conditions for 3 d (Recovery) and then harvested (T2). Three independent leaf rank samples (containing 12 leaves each) were harvested at T1 and T2 between 10.00h and 12.00h and stored at –80 C for further analyses.

In field experiments, the spring barley (*Hordeum vulgare* L.) cultivar Carina was sown using a drill on 2 April 2013 at Hohenschulen research farm, 15.5 km west of Kiel. The barley was managed organically (70kg N ha^−1^). Four replicate plots of 150 m^2^ each with 300 plants m^–2^ and 12.5cm of row distance were established. Thirty flag leaves from the main shoots were harvested from each plot between 10.00h and 12.00h and immediately stored at –80 °C for further analyses. Three plants were harvested from each plot and dark adapted for 30–45min for further photosynthesis and CO_2_ assimilation measurements. Harvests were performed 91 DAS (T0), 96 DAS (T1), and from T1 every 2 d for 2 weeks. Senescence was monitored by measuring chlorophyll contents (SPAD), photosystem II efficiency using a photosynthesis yield analyser (Mini-PAM, H Walz Effeltrich, Germany), and CO_2_ assimilation using a GFS-3000 portable gas exchange fluorescence system (H Walz Effeltrich, Germany).

After harvesting, all plant material was immediately frozen in liquid nitrogen and ground to obtain a fine homogenous powder. This powder was stored at –80 °C for further analysis.

### Determination of total nitrogen and carbon contents

Fifty milligrams of ground frozen plant material was weighed and freeze dried. Five milligrams of dry material were weighed in tin capsules to determine total N and C contents using a FLASH 2000 Organic Elemental Analyzer (Thermo Fisher Scientific, Villebon, France).

### Chlorophyll, ammonium, total amino acid, and total soluble protein determinations

Chlorophyll content was determined spectrophotometrically in crude leaf extracts according to Arnon (1949), (see Supplementary Annex S1 at *JXB* online). Total soluble protein was extracted in 50mM TRIS–HCl, pH 7.6 buffer and protein was determined using a commercially available kit (Coomassie Protein assay reagent, Bio-Rad, Hercules, CA). Total amino acid and ${\text{NH}}_{4}^{+}$ contents were determined after extraction in a 2% (w/v) solution of 5-sulphosalicylic acid by the Rosen colorimetric method using glutamine as a reference (Rosen, 1957).

### Glutamine synthetase and protease activity measurements

Enzymes were extracted from frozen ground leaf material stored at –80 °C in 50mM TRIS–HCl (pH 7.5), 1mM EDTA, 1mM MgCl_2_, 0.5% polyvinyl pyrrolidone (w/v), 0.1% β-mercaptoethanol (v/v), and 2× protease inhibitor cocktail complete EDTA-free (Roche). GS activity was measured according to Masclaux *et al.* (2000) The total soluble protein content was determined in the crude leaf extracts used for GS activity measurement using a commercially available kit (Coomassie Protein assay reagent, Bio-Rad, Hercules, California, USA). Analyses of endo- and exo-proteolytic activities were performed according to Guiboileau *et al.* (2013).

### RNA purification and RT-qPCR analysis

RNA isolation was performed with TRIzol reagent (Ambion) according to the manufacturer’s specifications. RNA suspended in nuclease-free water was stored at –80 °C. cDNA synthesis was performed using the first strand cDNA synthesis kit (Thermo Scientific). The qPCR mix was composed of 10 µl of MESA FAST qPCR master mix plus for SYBR assay (Eurogentec), 3.8 µl of water, 1.2 µl of 10 µM specific forward and reverse primers, and 5 µl of diluted cDNA 1:30 (v/v) in nuclease free water. Reactions were carried out in triplicate in 96 well plates in a Bio-Rad CFX connect thermocycler using the following program: 94 °C for 5min followed by 39 cycles of 94 °C for 5 s and 72 °C for 20 s sequences. Melt curves from 50 °C to 95 °C increasing by 0.5 °C every 30 s were performed. Fluorescence readings were taken during the elongation step (72 °C). Ct values were calculated by the CFX connect software. Genes and primers are listed in Supplementary Table S1 at *JXB* online. Several reference genes (including *GADPH*, *Actin*, *SAMd*, *CHS90*, *α-Tubulin*, *β-Tubulin*, *EF1a*, *ADPrf1*, *CDC48*, and *Ubiquitin*) were validated across all samples and conditions in accordance with the geNorm algorithm. The *GADPH* and *Actin* were used to calculate relative gene expression values of plant and flag leaf samples because they showed the lowest variation across samples and conditions.

### Protein separation, gel electrophoresis, and Western blot analysis

Proteins were extracted at 4 **°**C in TRIS–HCl (25mM, pH 7.5), EDTA (0.5mM), and 2× protease inhibitor cocktail complete EDTA-free (Roche) and denatured at 70 °C for 10min after adding one volume of NuPAGE LDS sample buffer 4×1: 0.1 (v/v) of NuPAGE sample reducing agent 10× (Life Technologies) to three volumes of protein extract. Proteins were then separated by SDS-PAGE on 10% polyacrylamide gels; equal amounts of protein were loaded in each lane. Denatured proteins were either transferred to PVDF membranes or stained directly in the gel with Coomassie blue for GS and Rubisco detection, respectively. For GS blotting, polyclonal antibodies were used for the detection of both GS1 and GS2 isoenzymes (Lemaître *et al.*, 2008). Antibodies against barley N-terminal and C-terminal Rubisco were kindly provided by Dr Urs Feller (University of Bern, Switzerland).

### Analysis and description of barley *HvGS* and *HvASN* genes

*HvGS2* and three isoforms of *HvGS1* (*GS1_1, GS1_2*, and *GS1_3*) were characterized by Baima *et al.* (1989) and Goodall *et al.* (2013), respectively. Two more sequences with similarities to prokaryotic *GS* genes were found through BLAST using *A. thaliana* [*AtGLN1.1* (NM_123119, *At*5g37600), *AtGLN1.2* (NM_105291, *At*1g66200), *AtGLN1.3* (NM_112663, *At*3g17820), *AtGLN1.4* (NM_121663, *At*5g16570), and *AtGLN1.5* (NM_103743, *At*1g48470)] and *O. sativa* GLN1 predicted the protein sequences [*OsGLN1_1* (NM_001054580.1, *Os*02g0735200), *OsGLN1_2* (NM_001055959.2, *Os*03g0223400), and *OsGLN1_3* (NP_001051067, *Os*03g0712800)] as queries in Genbank (www.ncbi.nlm.nih.gov/nuccore) and EnsemblPlants (http://plants.ensembl.org/Multi/enasearch) databases (see Supplementary Table S2 at *JXB* online). Primers for the *HvASN1* and *HvASN2* genes previously described by Moller *et al.* (2003) were designed. Other *HvASN* genes were found through the BLAST algorithm using *Arabidopsis* [*AtASN1* (*At*3g47340.1, NM_114602.3), *AtASN2* (*At*5g65010.2, NM_180941.2), and *AtASN3* (*At*5g10240.1, NM_121062.4)], rice [*OsASN1* (NM_001048300, *Os*12g38630), *OsASN2* (*Os*03g18130), *OsASN3* (*Os*06g15420), *OsASN4* (*Os*01g65260), and *OsASN5* (*Os*05g35580)], and maize [*ZmASN1* (GRMZM2G074589), *ZmASN2* (NM_001137541, GRMZM2G093175), *ZmASN3* (NM_001137542, GRMZM2G053669), and *ZmASN4* (NM_0011 37543, GRMZM2G078472)] predicted protein sequences as queries. Contigs for *HvGS* and *HvASN* genes were found through alignments with EST sequences with the barley genome and gene structure was obtained through contig and EST alignments using ApE plasmid editor (http://biologylabs.utah.edu/jorgensen/wayned/ape/) and Exon–Intron graphic maker (http://wormweb.org/exonintron). Multiple protein sequence alignments and phylogenetic trees were generated using the ClustalW algorithm (see Supplementary Tables S3, S4, and S5 at *JXB* online).

### Statistical analysis

For all data, *t* tests were used to determine significant differences using Excel (Microsoft Office 2011) software.

## Results

### Characterization of leaf senescence in barley leaves using known metabolic markers

Leaf senescence in barley was studied comparing leaf ranks of plants at the vegetative stage and comparing flag leaves harvested methodically during development. The induction of genes by stress-induced senescence via nitrate limitation or dark treatment was investigated on plants at the vegetative stage.

**Fig. 1.**
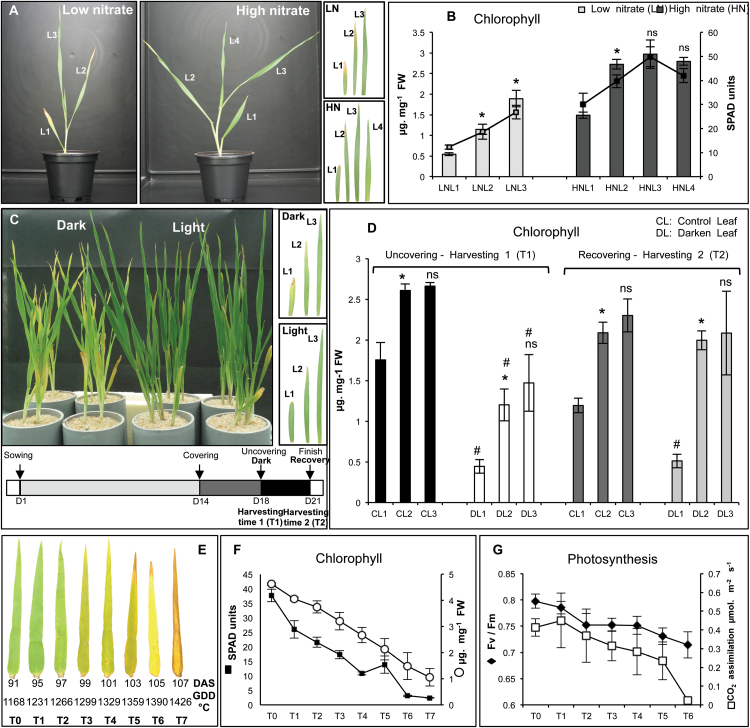
Changes in chlorophyll and photosynthesis during leaf senescence in barley. (A, B) Leaves of plants grown under low (LN) and high (HN) nitrate conditions. (C, D) Leaves of plants submitted or not to dark treatment. T1: 4 d of dark treatment. T2: 3 d of recovering under day/night conditions after T1. CL (control untreated leaves: black and dark grey bars), DL (darkened leaves: white and light grey bars). (E) Flag leaves harvested at different time points after heading (from T0 to T7). DAS (days after sowing); GDD °C (growing degree days in °C). (F) Chlorophyll contents in flag leaves measured by SPAD (open circles) and spectrophotometer (solid squares). (G) Photosystem II efficiency (solid circles) and CO_2_ assimilation (open squares) in flag leaves. All data represent mean ±SD of 3–4 biological replicates. (B, D) The ns and the asterisks (*) indicate, respectively, the non-significant and significant differences between leaf (n+1) and leaf (n) in each treatment or growth condition. (D) The # symbol indicates the significant differences existing between each leaf rank of control and dark-treated plants at each time point. Significance was evaluated using a *t* test with *P* <0.05.

In plants grown under low (LN) or high (HN) nitrate conditions, chlorophyll content decreased with ageing in all leaf ranks (Fig. 1A, B). Comparing the same leaf rank of plants under LN and HN, *t* test analysis showed significantly (*P* <0.05) lower chlorophyll content in LN samples (Fig. 1A, B). An age-related decrease in chlorophyll concentrations was observed in the dark-treated plants and in their untreated controls (Fig. 1C, D). The dark-exposed leaves showed less chlorophyll than control leaves at T1. After 3 d of recovery in the light, chlorophyll concentrations in the dark-exposed leaves were the same as in the control leaves (Fig. 1D). A steady decrease in chlorophyll and photosynthesis was observed in flag leaves from T1 to T7 (Fig. 1F, G) showing the gradation of leaf senescence throughout flag leaf development.

### Changes in carbon and nitrogen contents in barley leaves during senescence

**Fig. 2.**
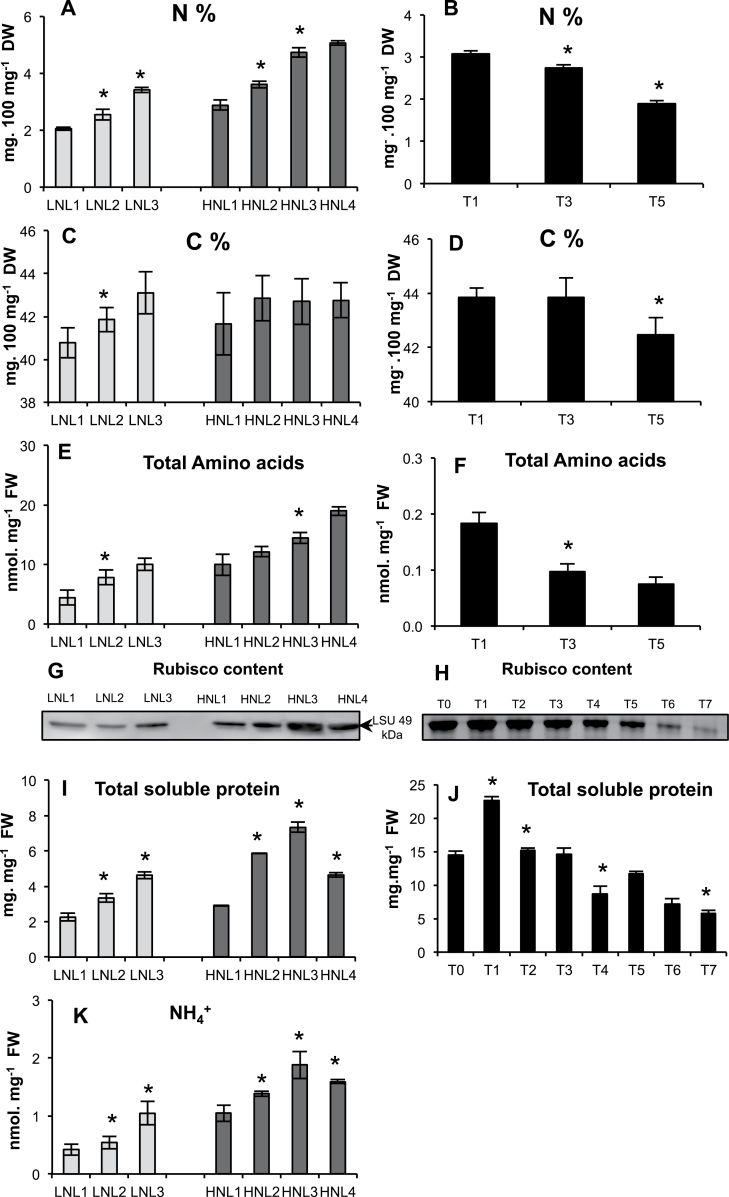
Changes in carbon, nitrogen, and nitrogen-containing compounds during leaf senescence. Leaf ranks of plants grown under low (LN; light grey) and high (HN; dark grey) (A, C, E, G, I, K) and flag leaves (B, D, F, H, J) were analysed. In flag leaves, only total protein (J) and Rubisco contents (H) were measured at eight time points (T0 to T7). Rubisco content was determined using antibodies against the barley Rubisco C-terminal. For gels, equal protein amounts were loaded in each lane. Experiments were repeated twice and gave similar results. Data represent mean ±SD of 3–4 biological replicates. (A, C, E, I, K) The asterisks (*) indicate significant differences between leaf (n+1) and leaf (n) under the same nitrate conditions. (B, D, F, J) The asterisks (*) indicate the significant differences occurring during ageing. Significance was evaluated using a *t* test with *P* <0.05.

Nitrogen concentration (N%) decreased significantly with ageing between leaf ranks and in flag leaves. In the oldest leaves, the N% was close to 50% of the levels in the youngest (Fig. 2A, B). By contrast, the carbon concentration (C%) remained stable or decreased slightly with ageing (Fig. 2C, D). Total amino acids, total soluble protein, ${\text{NH}}_{4}^{+}$, and Rubisco decreased steadily with ageing (Fig. 2E–K), except in HNL3 compared with HNL4. Similarly, protein patterns in flag leaves confirmed that the flag leaf at T0 is a young developing leaf and that the flag leaf at T1 is mature.

### Changes in nitrogen remobilization enzymes during leaf senescence

**Fig. 3.**
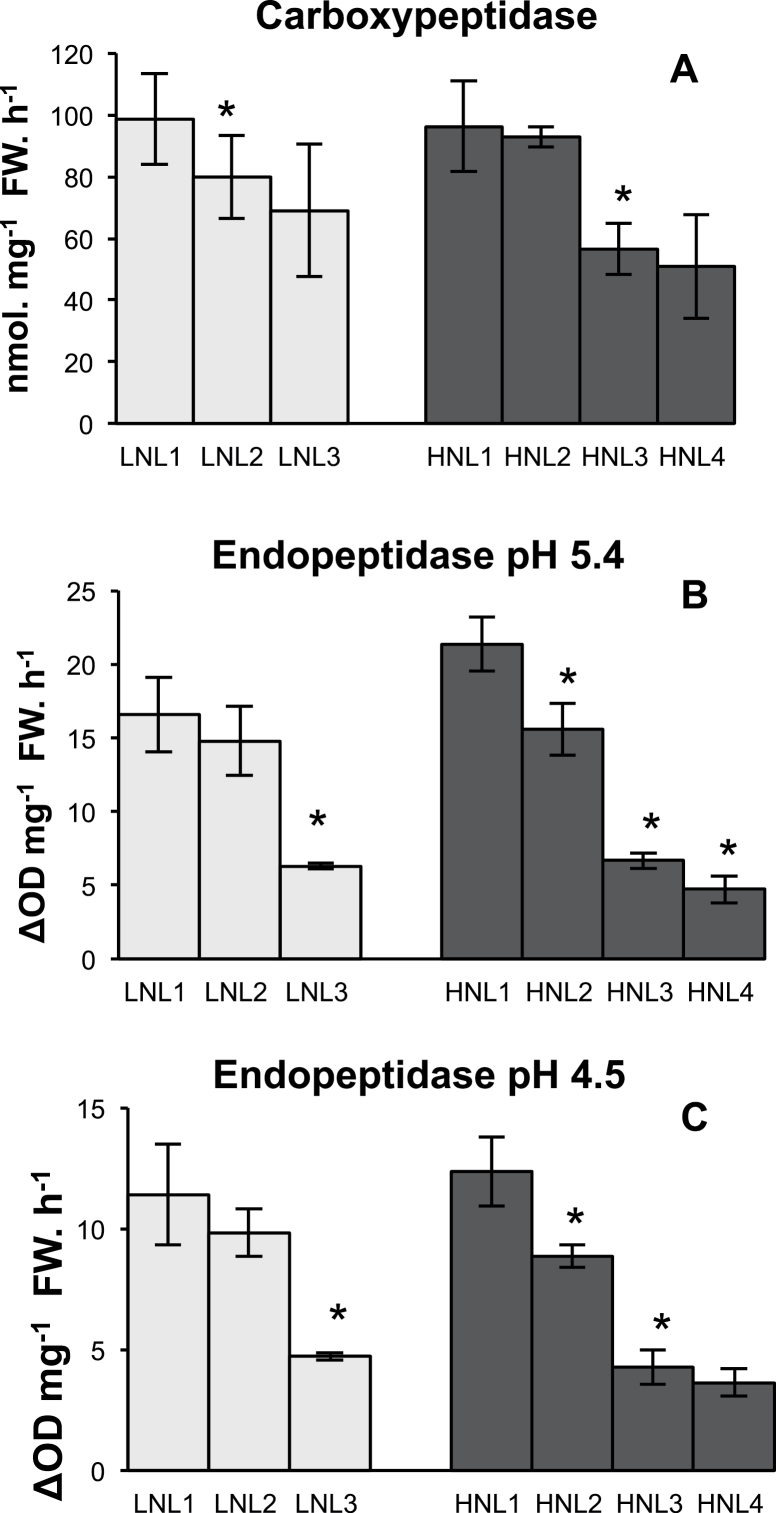
Protease activities are increased in old leaves of plants. Carboxypeptidase (A) and endopeptidase at pH 5.4 (B) and at pH 4.5 (C) were measured. LN (low nitrate; light grey) and HN (high nitrate; dark grey). Data represent mean ±SD of 3–4 biological replicates. The asterisks (*) indicate the significant differences between leaf (n+1) and leaf (n) under the same nitrate conditions. Significance was evaluated using a *t* test with *P* <0.05. Similar results have been obtained on two independent cultures.

**Fig. 4.**
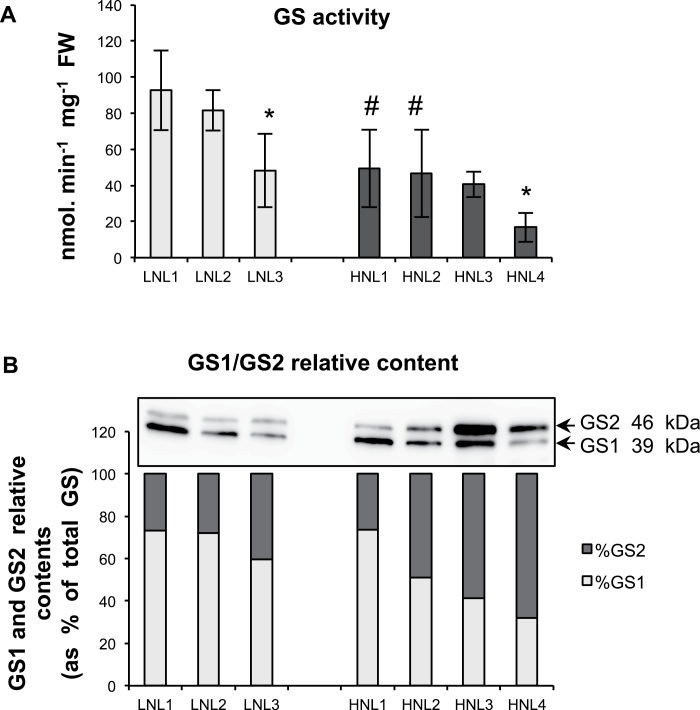
Glutamine synthetase (GS) activity and protein contents in leaf ranks of plants. (A) GS activity. Data are mean ±SD of 3–4 biological replicates. The asterisks (*) indicate the significant differences between leaf (n+1) and leaf (n) under the same nitrate conditions. The symbol (#) indicates significant differences between LN and HN for the same leaf rank. Significance was evaluated using a *t* test with *P* <0.05. LN (low nitrate; light grey) and HN (high nitrate; dark grey). (B) GS1 (39kDa) and GS2 (46kDa) were identified on Western blots. GS1 and GS2 proportions were calculated after quantification of signals using densitometry and ImageJ imaging software. Equal protein amounts were loaded in each lane. All experiments were performed on two different cultures giving the same results.

Carboxypeptidase, endopeptidase (pH 5.4 and pH 4.5), and GS activities are well-known markers of both leaf senescence and nitrogen remobilization. Both were monitored in leaf ranks from plants grown under LN and HN conditions. All activities were significantly higher in old leaves compared with young ones under both LN and HN (Figs 3, 4A). For the same leaf rank, the magnitude of protease activity was quite similar in the two nitrate conditions. By contrast, for L1 and L2, total GS activity was higher in the LN leaf compared with the corresponding HN leaf. Western blot analysis allowed the GS1 and GS2 isoform contents to be semi-quantified and to estimate their respective proportions (Fig. 4B). GS1 relative content increased with leaf ageing, especially in plants grown under HN (HNL1 and HNL2 compared with HNL3 and HNL4). The relative content of GS1 was already high (60%) in young leaves of LN-grown plants, suggesting that *HvGS1* expression is higher under LN compared with HN, which was confirmed subsequently for the *HvGS1_1* gene (see below).

### Characterization of the glutamine synthetase (*GS)* and asparagine synthetase (*ASN*) genes

**Fig. 5.**
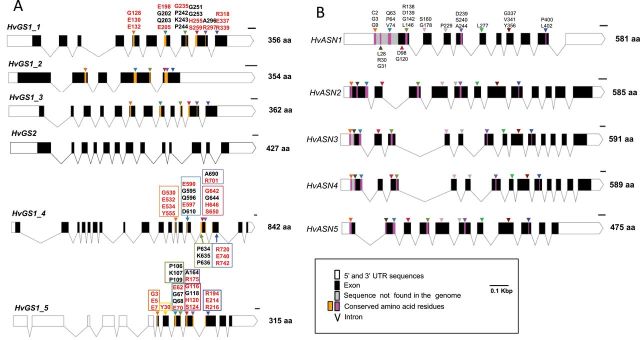
Description of barley *HvGS* and *HvASN* gene structures. Diagrams of *HvGS* (A) and *HvASN* (B): white boxes represent untranslated regions, black boxes represent coding regions, solid V lines represent introns, yellow and pink boxes indicate the conserved amino acid residues among GS and AS proteins, respectively, from barley (*H. vulgare*), *Z. mays, O. sativa, A. thaliana*, *T. aestivum, S. officinarum, V. vinifera, P. sativum, L. perenne, S. bicolor, S. smithii, D. melanogaster, H. sapiens, A. nidulans, S. filamentosus, E. coli*, and *S. aureus.* Conserved groups of amino acid residues in all *Hv*GS1 and *Hv*AS proteins analysed are indicated in black font and by coloured arrowheads. Amino acid residues responsible for ligand binding specificity in the GS family are indicated in red font and by coloured arrowheads. The predicted amino acid (aa) length for each of the corresponding proteins is shown on the right. (This figure is available in colour at *JXB* online.)

The barley *HvGS2* mRNA sequence encoding the GS2 isoform has previously been reported by Baima *et al.* (1989). Three different *HvGS1* cDNAs encoding three isoforms of GS1 have been described by Goodall *et al.* (2013). In this report, two additional *HvGS1* mRNA sequences were identified by alignment analysis of barley ESTs with *GS* counterparts from *Arabidopsis* (*A. thaliana*), rice (*O. sativa*), and maize (*Z. mays*) as queries (Table 1). In the phylogenetic tree (see Supplementary Fig. S1 at *JXB* online), the predicted proteins of these two new isoforms were clustered with the GS proteins of prokaryotes (Mathis *et al.*, 2009; Swarbreck *et al.*, 2011; Table 1). Conserved amino acid residues essential for ligand binding specificity of the GS1 protein family (Gly^128^, Glu^130^, Glu^132^, Tyr^158^, Glu^198^, Val^199^, Glu^205^, Asn^250^, Gly^251^, His^255^, Ser^259^, Arg^318^, Glu^337^, Arg^339^) (van Rooyen *et al.*, 2011) were found in all the GS protein sequences (see Supplementary Fig. S2 and Supplementary Table S4 at *JXB* online). Based on this analysis, these two supplementary sequences were named *HvGS1_4* and *HvGS1_5*. *HvGS1_4* is predicted to encode a long protein of 842 amino acids homologous to the prokaryotic GSI-type. *HvGS1_5* is predicted to encode a peptide of 315 amino acids that presents homologies with the prokaryotic GSIII-type proteins (Table 1; Mathis *et al.*, 2009). All the *HvGS1* sequences correspond to two different contigs in the barley genome. Gene sequences were aligned to the barley genomic sequence in order to deduce 5′ and 3′ untranslated regions, introns and exons (see Supplementary Table S3 at *JXB* online) and to establish the *HvGS* gene models (Fig. 5A).

**Table 1.** *HvGS* and *HvASN* genes and proteins

Gene in *Hordeum* *vulgare*	Gene code	NCBI gene accession number	NCBI protein accession number	BAC clone	Contig	No. of amino acid residues	% Identity of *H. vulgaris* to			
							*A. thal.*		*O. sat.*	*Z. mays*
*HvGS1_1*	MLOC_11890	JX878489	AFX60875	–	x_contig_1562081	356	85.67 ^a^	14.41^ b^	92.70^ a^	83.15^ a^
*HvGS1_2*	No gene reported	JX878490	AFX60876	–	x_contig_1569958	354	84.18 ^a^	14.78^ b^	84.18^ a^	86.72^ a^
*HvGS1_3*	MLOC_62030	JX878491	AFX60877	–	x_contig_46131	362	81.97^ a^	15.76^ b^	83.10^ a^	81.69^ a^
*HvGS1_4*	MLOC_59238	AK252215	–	FLbaf147e20	x_contig_43390	842	15.65^ a^	61.55^ b^	15.94^ a^	16.47^ a^
*HvGS1_5*	No gene reported	AK365395	BAJ96598	NIASHv2033O14	x_contig_1558692	315	21.97^ a^	57.46^ b^	21.97^ a^	20.98^ a^
*HvGS2*	MLOC_54057	AK360336	BAJ91545	NIASHv1115P04	x_contig_38845	427	–	–	–	–
*HvASN1*	MLOC_63089	AK359770	BAJ90979	NIASHv1051G03	X_contig_47260	585	80.90^*c*^		77.45^*d*^	76.42^*c*^
*HvASN2*	MLOC_75057	AK357350;	BAJ87368;	NIASHv1102L13	X_contig_6705	581	79.35^*c*^		75.30^*d*^	75.47^*c*^
		AK373732	BAK04929							
*HvASN3*	MLOC_37219	AK353762	BAJ84981	NIASHv1002J05	X_contig_2547996	591	75.65^*c*^		91.17^*d*^	88.12^*c*^
*HvASN4*	MLOC_72774	AK363899	BAJ95102	NIASHv2019N19	X_contig_6234	589	75.78^*c*^		91.82^*d*^	88.78^*c*^
*HvASN5*	MLOC_44080	AK361923	BAJ93127	NIASHv2001E23	X_contig_274144	475	70.68^*c*^		68.99^*d*^	70.04^*c*^

^*a*^ Proteins aligned with *AtGS1_1*, *OsGS1_1*, and *ZmGS1_1*.

^*b*^ Proteins aligned with *AtGS1_6.*

^*c*^ Proteins aligned with *AtASN1* and *ZmASN1.* ^c^

^*d*^ Proteins aligned with *OsASN3*

Barley *HvASN1* and *HvASN2* sequences have been described by Moller *et al.* (2003). Three new *HvASN* mRNA sequences were found by alignment analyses using the *ASN* sequences from *Arabidopsis*, rice, and maize as queries (see Supplementary Table S2 at *JXB* online). All five *HvASN* sequences correspond to five different contigs in the barley genome (Table 1). In the phylogenetic tree, two predicted proteins (HvASN1 and HvASN2) were clustered with AtASN1 (class I), two (HvANS3 and HvASN4) with AtASN2 (class II), and the fifth predicted protein (HvASN5) was placed away from the branch of *Arabidopsis* proteins. Nevertheless, it was clustered with AS proteins from rice and maize (see Supplementary Fig. S3 at *JXB* online). The predicted proteins of all five isoforms showed between 67% and 90% conservation of the amino acid sequence with AS proteins of maize, rice, and *Arabidopsis* (see Supplementary Table S5 at *JXB* online). These conserved regions contain amino acid residues from the *purF*-type glutamine binding domain (Cys^2^, Asp^34^, His^104^), essential amino acids for glutamate binding and positioning (Arg^50^, Leu^51^, Ile^53^, Asn^75^, Gly^76^, Glu^77^, Asp^98^), and essential residues for binding of aspartate and ATP (Thr^316^, Thr^317^, Arg^319^) (see Supplementary Fig. S4 at *JXB* online). *HvASN* sequences were aligned to the genomic sequence in order to obtain gene models (see Supplementary Table S3 at *JXB* online; Fig. 5B). Based on the sequences found for the *HvGS* and *HvASN* genes, primers were designed for qPCR detection and *HvGS* and *HvASN* transcript levels were monitored during natural and induced senescence.

### Changes in *HvGS* and *HvASN* transcript levels in plants grown under nitrate-limiting conditions

**Fig. 6.**
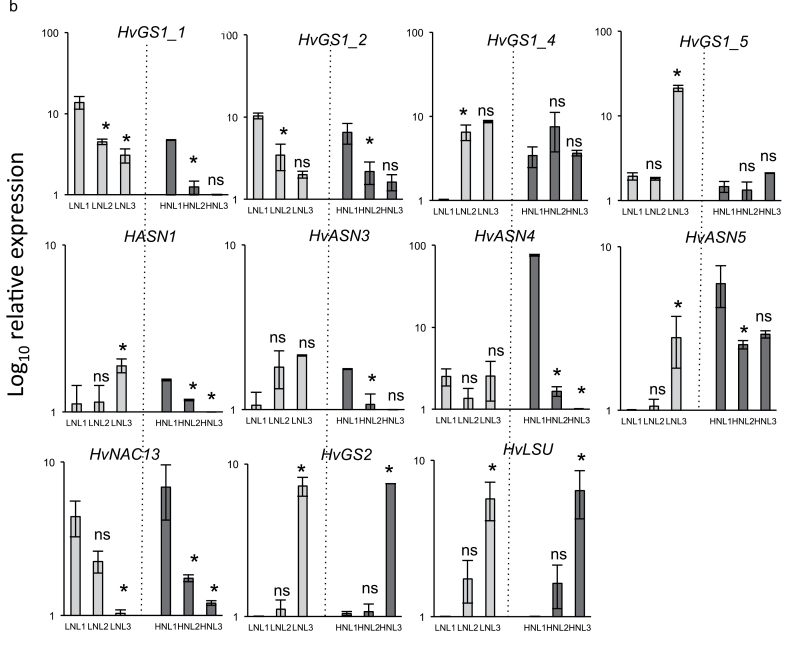
Transcript levels of *HvGS*, *HvASN*, *HvNAC13,* and *HvLSU* genes in leaves of plants grown under low (LN) and high (HN) nitrate conditions. Only leaf ranks L1, L2, and L3 from LN (grey bars) and HN (black bars) plants were analysed. Both line charts and histograms are presented for *HvASN* (LN: light grey line; HN: dark grey line). Log_10_ relative expression values are shown. Data are means ±SD (*n*=3 biological replicates). The ns and asterisks (*) indicate, respectively, the non-significant and significant differences between leaf (n+1) and leaf (n) under the same nitrate conditions. Significance was evaluated using a *t* test with *P* <0.05. *HvGAPDH* was used as the reference gene.

Changes in the transcript levels were measured in leaf ranks of plants grown under low or high nitrate conditions. As a control, the expression of the senescence-associated *HvNAC13* and the senescence repressed *HvLSU* genes were also monitored (Christiansen *et al.*, 2011; Hollmann *et al.*, 2014). As expected, *HvNAC13* expression increased with leaf senescence while *HvLSU* decreased (Fig. 6). *HvGS2,* which is another well-known senescence-repressed gene, was down-regulated during leaf ageing.

The *HvGS1_1* and *HvGS1_2* expression levels increased in old leaves under both HN and LN (Fig. 6). Expression of *HvGS1_1* was higher under LN relative to HN. By contrast, the two prokaryote-like *HvGS1_4* and *HvGS1_5* genes decreased with ageing, but only when plants were grown under low-nitrate nutrition. Under high nitrate, no difference in expression levels were noticed between the leaf ranks (Fig. 6). All attempts to measure *HvGS1-3* expression levels were unsuccessful for unknown reasons (Fig. 6).

The effect of leaf ageing on *HvASN* transcript levels was more surprising. Indeed, all the *HvASN* transcript levels (except *HvASN4*) decreased with ageing under LN conditions and increased under HN. This contrasting nitrate-dependent effect of leaf ageing has never been described before. *HvASN4* gene expression, which remained steady under LN, was sharply induced under HN similar to the other *HvASN* genes (Fig. 6). *HvASN2* expression levels could not be measured because no specific primers could be found for this gene.

### Changes in *HvGS* and *HvASN* transcript levels in flag leaves during senescence

**Fig. 7.**
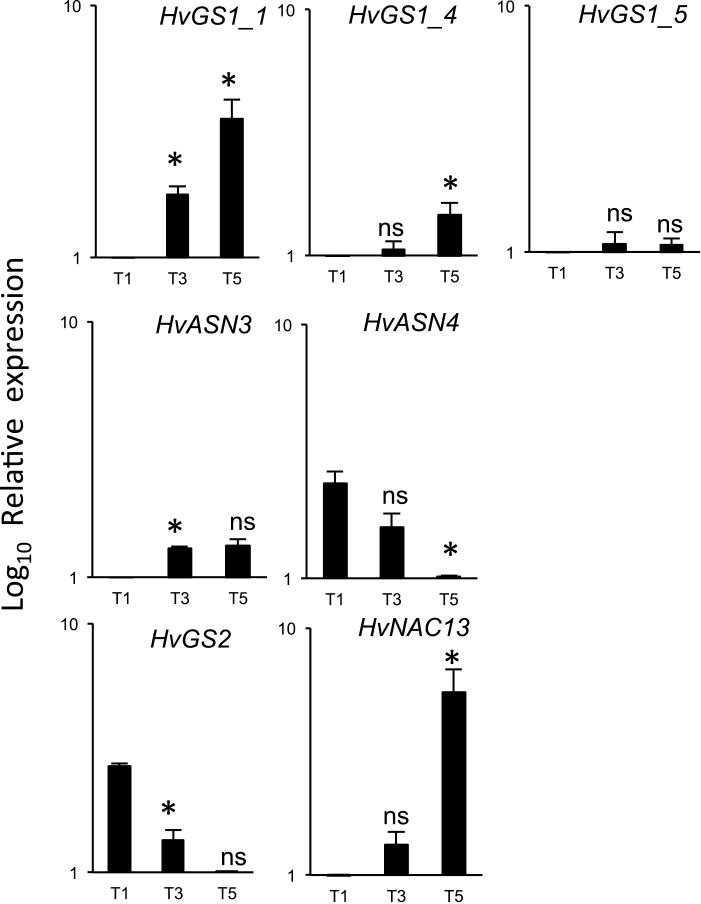
Transcript levels of *HvGS*, *HvASN,* and *HvNAC13* genes in flag leaves harvested at different stages of senescence. Young leaves (T1), mature leaves (T3), and senescing leaves (T5) were analysed. Log_10_ relative expression values are shown. Data are mean ±SD (*n*=3 biological replicates). The ns and asterisks (*) indicate, respectively, the non-significant and significant differences occurring during ageing, i.e. between T3 and T1 and between T5 and T3. Significance was evaluated using a *t* test with *P* <0.05. *HvActin* was used as the reference gene.

Unfortunately, and certainly because of gene polymorphisms between Golden Promise and Carina genotypes, the expression of only *HvGS1_1*, *HvGS1_4*, *HvGS1_5*, *HvGS2*, *HvASN3*, and *HvASN4* could be monitored in the flag leaf samples. The primers identified for the other *HvGS* and *HvASN* genes on the Golden Promise genomic sequence could not be used for real-time RT-qPCR on Carina samples. The *HvGS1_1* and *HvGS1_4* transcript levels gradually increased from T1 to T5 in parallel with *HvNAC13*, which was opposite to the *HvGS2* transcripts (Fig. 7). *HvGS1_5* gene expression did not change with ageing. The *HvASN3* mRNA level slightly increased in old leaves (T3 and T5) compared with young ones (T1). *HvASN4* gene expression decreased from T1 to T5 showing a completely opposite pattern to *HvASN3* (Fig. 7).

### Changes in *HvGS* and *HvASN* transcript levels in leaf ranks of plants grown under dark-stress conditions

**Fig. 8.**
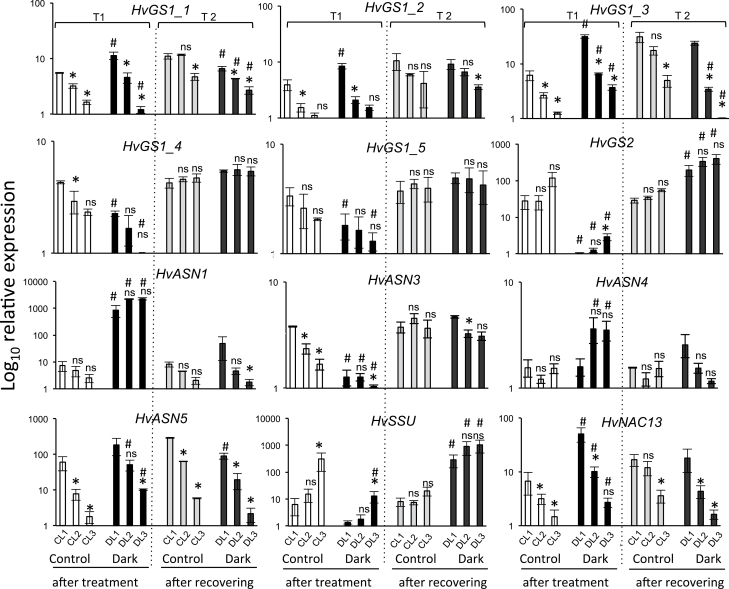
Transcript levels of *HvGS*, *HvASN*, *HvNAC13,* and *HvSSU* genes in leaves of plants following dark treatment and recovery. CL (control leaves at T1: white; control leaves at T2: light grey); DL (darkened leaves at T1: black; darkened leaves at T2: dark grey). Log_10_ relative expression values are shown. Data are means ±SD (*n*=3 biological replicates). The ns and asterisks (*) indicate, respectively, the non-significant and significant differences between leaf (n+1) respective to leaf (n) in control and dark-treated plants. The symbol # indicates the significant differences existing between each leaf rank of control and dark-treated plants at each time point. Significance was evaluated using a *t* test with *P* <0.05. *HvGAPDH* was used as the reference gene.

In order to observe the effect of dark-induced senescence on *HvGS* and *HvASN* genes, transcript levels were evaluated in each leaf rank after 4 d of dark treatment and also after a recovery (a similar photoperiod as before the dark treatment) time of 3 d (Fig. 1C). Expression of these genes in leaf ranks of dark-treated plant (DL) and control plants gave global confirmation of the age-dependent trends found previously in the HN samples (Fig. 6). Expression levels were then compared between untreated controls (CL) and dark-treated samples for each leaf rank. This allowed the effect of darkness on gene expression, and also the recovery ability of each gene, to be determined. *HvNAC13* and *HvSSU* were used as dark-senescence-induced and -repressed controls, respectively (Gregersen *et al.*, 2008; Fig. 8). The dark effect on the *HvGS2* expression level was quite similar to *HvSSU. HvGS1_1* and *HvGS1_2* transcript levels slightly increased after dark treatment and this effect was more pronounced in the old leaves than in the young leaves. The *HvGS1_*3 expression level sharply increased in all the dark-treated leaves compared with the controls. By contrast, *HvGS1_*4 and *HvGS1_*5 transcription levels were lower in dark-treated leaves than in the controls, showing a similar trend to *HvGS2.* For the *HvASN* genes, two opposite trends were also observed. *HvASN1*, *HvASN4*, and *HvASN5* transcript levels were highly increased after dark treatment while *HvASN3* was highly repressed by darkness.

After recovery time (T2), *HvGS* and *HvASN* transcript levels were similar in dark-treated plants to those from the control plants, with the exception of *HvGS2* and *HvSSU* (Fig. 8). Indeed, these two genes were expressed at greater levels in plants after the recovery time than in the control. As they both encode chloroplast enzymes, this higher expression in dark-treated leaves after light exposure during recovery could be related to the need of leaves to restore chloroplast functions that have been lost during the dark treatment.

## Discussion

The aim of this study was to provide a picture of the expression of the *HvGS1* and *HvASN* genes in response to leaf senescence, nitrogen supply, and dark exposure. Natural and starvation-induced senescence were characterized using several biochemical markers that have already been largely described in other plant species such as tobacco, *Arabidopsis*, sunflower, oilseed-rape, and maize (Masclaux *et al.*, 2000; Diaz *et al.*, 2005, 2008; Hirel *et al.*, 2005*a*, *b*; Niewiadomska *et al.*, 2009; Orsel *et al.*, 2014). As such, measurements of chlorophyll content, photosynthesis, and senescence-associated gene expression led to the identification of young, mature, and senescing leaves in each experiment. On both flag leaf and leaf rank models, the same picture of leaf senescence was observed globally, i.e. a decrease in all nitrogen compounds, an increase in N remobilization markers such as GS1 proteins, endoprotease, and carboxypeptidase activities, and a decrease in the Rubisco and GS2 protein contents (see Masclaux-Daubresse *et al.*, 2010 and Avila-Ospina *et al.*, 2014 for reviews).

However, and by contrast with other plant species like *Arabidopsis* or tobacco, the total GS activity surprisingly increased significantly with ageing (Masclaux *et al.*, 2000; Martin *et al.*, 2006; Diaz *et al.*, 2008). GS activity was also slightly higher in the LN leaves compared with the HN ones, as also described in *Arabidopsis* by Lemaître *et al.* (2008). Because it was observed that the GS1 isoforms were more abundant under LN and in old leaves than under HN and young leaves, respectively, it was concluded that the increase in total GS activity observed here during senescence and under low nitrate is mainly due to GS1 activity. It is then likely that, in barley, as in other monocots like maize and rice, GS1 is predominant over the GS2 isoenzyme (Tabuchi *et al.*, 2005; Martin *et al.*, 2006).

Our aim was then to identify the glutamine synthetase and the asparagine synthetase genes of barley in order to analyse their response to natural and stress-induced senescence. Several of them had already been described. Goodall *et al.* (2013) had identified the three *HvGS1_1, HvGS1_2*, and *HvGS1_3* sequences. These authors showed that *HvGS1_*3 is more highly expressed in the grain, *HvGS1_1* in the stem, and *HvGS1_2* in the leaf and root. *HvGS1_3* is more highly expressed when ammonium is provided as the sole nitrogen source. While the same authors characterized *HvGS1* expression in response to nitrate and ammonium supply, they did not provide data about their responses to leaf senescence. Moller *et al.* (2003), who described the two *HvASN1* and *HvASN2* genes, focused on the up-regulation of *HvASN1* induced by darkness.

**Fig. 9.**
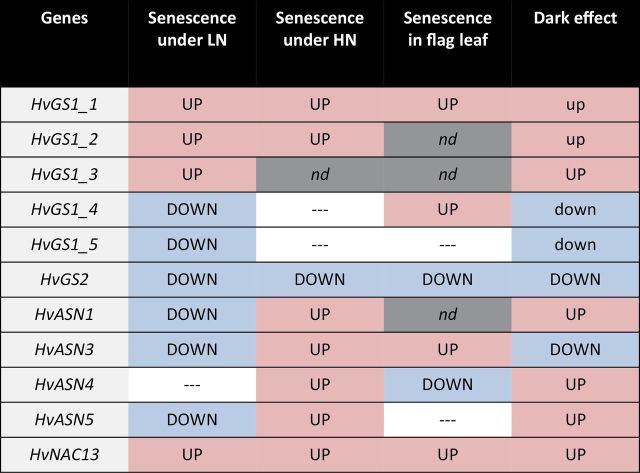
Up and down regulation of *HvGS1* and *HvASN* depending on natural or stress-induced senescence. The significant increases and decreases in transcript levels in senescing tissues are indicated by UP and DOWN, respectively, when the variation observed is high and by up and down when the senescence effect is significant but not as high. nd means not determined and — indicates that there was no effect of senescence on transcript levels. Blue cells are for down-regulation, pink cells for up-regulation, white cells are for no difference, and grey cells for nd. (This figure is available in colour at *JXB* online.)

Using a barley genome sequence, two putative *HvGS1_4* and *HvGS1_5* genes were identified that are more similar to the prokaryotic-like forms (Nogueira *et al.*, 2005; Mathis *et al.*, 2009). Three types of *GS* genes, GSI, GSII, and GSIII, have been identified in eukaryotes and prokaryotes (Ghoshroy *et al.*, 2010). The most studied *GS* in plants is the GSII-type composed of the plastidial *GS2* and the cytosolic *GS1* isoforms. The GSI-type is commonly found in prokaryotes (Merrick and Edwards, 1995; van Rooyen *et al.*, 2011), however, GSI-type genes have also been found in many dicot and monocot plants (Swarbreck *et al.*, 2011). These GSI-type genes are characterized by being longer than GSII-type genes and code for longer proteins than GSII-type products. For example, the length of the GSI-type gene in rice is 6790bp, while the length of the GSII-type *GS1;1* is 3696bp. The GSI-type product in rice has 845 amino acid residues, while the GSII-type GS1;1 has 356 amino acids. Although the GSI prokaryotic-like forms have been reported in several plant species including *Arabidopsis* (*At3g53180*; see Supplementary Table S2 at *JXB* online) and wheat, their exact enzymatic roles remain enigmatic in plants because no enzyme purification or mutant analysis has ever been performed. For the GSIII-type prokaryotic forms, no full sequence has ever been found in plant genomes, but fragments of GSIII-type prokaryotic forms have been identified in several eukaryotic species and plants, such as the *HvGS1_5* in barley (Swarbreck *et al.*, 2011). The expression patterns of the two GSI- and GSIII-type prokaryotic forms *HvGS1_4* and *HvGS1_5* were more similar to that of *HvGS2* than to *HvGS1_1*, *HvGS1_2*, and *HvGS1_3* (Fig. 9). Like HvGS2, they were also repressed by senescence at the vegetative stage and by the dark treatment. Studying the sub-cellular localization and activity of purified HvGS1_4 and HvGS1_5 might be interesting in order to determine if they can contribute to glutamine synthesis, similar to the GS2 isoenzymes, inside the chloroplasts or other organelles. By contrast with the prokaryotic forms, *HvGS1_1*, *HvGS1_2, and HvGS1_3* were induced by leaf senescence and by dark treatment, as has been observed for *GS1* genes in other plant species. Consequently, a role for these three genes in nitrogen remobilization during leaf senescence can be proposed.

In addition to the two *HvASN1* and *HvASN2* genes already described by Moller *et al.* (2003), three more *HvASN* mRNA sequences were found. From the barley genome sequence gene structures and specific primers for qPCR analyses for the *HvGS1* sequences were deduced. When measuring transcript levels in the different leaf ranks of plants grown under low or high nitrate conditions, it was surprised to see that *HvASN1*, *HvASN3*, and *HvASN5* were repressed by ageing under low-nitrate conditions, while under high-nitrate conditions all of them, including *HvASN4*, were induced by leaf senescence (Fig. 9). To our knowledge, such a nitrogen-dependent effect of leaf senescence on gene expression has never been described. The reason why the response of *HvASN* genes to ageing is different in leaves depending on nitrate nutrition is actually difficult to understand. Indeed, the regulation of *ASN* genes in plants is poorly known, even in *Arabidopsis*. The little information available on this subject is limited to sugar signalling effects. It has been reported in *Arabidopsis* that the *AtASN1* gene, also known as *DIN 6* (*Dark Inducible 6*), is repressed during the light period and also when sugar is provided (Oliveira *et al.*, 2002; Gaufichon *et al.*, 2010). The sucrose regulated transcription factor, bZIP11, regulates *AtASN1* expression in *Arabidopsis* (Hanson *et al.*, 2008). By contrast, it has been shown that *AtASN2* gene expression is enhanced by light and sugars and that this isoform certainly plays a role during the light period (Oliveira *et al.*, 2002). Because it is known that starch and sugar contents are much higher in plants grown under low-nitrate conditions and are modulated during leaf ageing (Diaz *et al.*, 2008; Lemaitre *et al.*, 2008), it can be hypothesized that the differences in senescence-related effects on the expression of the *HvASN* genes might result from differences in metabolite signalling.

Although the senescence effects on the different *HvASN* genes seemed quite homogenous at the vegetative stage, the picture was different in flag leaves. In the flag leaf, there was indeed more contrast due to senescence because the *HvASN3* mRNA level increased with ageing while *HvASN4* decreased. Such a contrast could again be due to the relatively different sugar and amino acid concentrations in seedlings and flag leaves. From the phylogenetic trees, it was deduced that *HvASN1* and *HvASN2* are more similar to the *Arabidopsis AtASN1* gene, while *HvASN3* and *HvASN4* are more similar to *AtASN2.* This is in good agreement with the fact that *HvASN1* is sharply induced by dark treatment (Moller *et al.*, 2003; this work), like *AtASN1*, and that *HvASN3* is dark repressed (this work) like *AtASN2*. For *HvASN4*, which is induced by dark treatment, such an analogy is, however, not upheld.

Whatever the effects of metabolites on the expression of *HvASN* genes, the results obtained from seedlings grown under low- or high-nitrate conditions suggest that, on a global level, asparagine synthetase (AS) is needed more in senescing leaves when plants are grown under high nitrate than when grown under nitrate-limiting conditions. This then suggests that asparagine synthesis could be important for nitrogen remobilization in plants grown with high levels of N fertilization. Under fertilizer limitation, GS1 and glutamine as a combination might be more essential than AS and asparagine, as shown by the relatively high GS1 protein level in the LN samples compared with the HN samples (Fig. 4).

It can be concluded from this study that, in barley, the three eukaryotic-like *HvGS1* genes identified previously by Goodall *et al.* (2013) show the typical senescence-induced profile described for *GS1* genes in other plant species. The two new prokaryotic-like *HvGS1* genes identified are, by contrast, repressed by leaf senescence and their expression pattern appears to be more similar to *HvGS2*. If these *HvGS1_4* and *HvGS1_5* genes indeed encode glutamine synthetase enzymes as proposed, they are probably not needed in old leaves for nitrogen remobilization. The expression of the five *HvASN* genes characterized here suggests that they are all needed in senescing leaves when plants are well fertilized with nitrate. Otherwise, they might have a predominant and complementary role in response to shading or during the dark/light periods.

## Supplementary data

Supplementary data can be found at *JXB* online.

Supplementary Fig. S1. Phylogenetic tree of the cytosolic glutamine synthetase 1 gene family.

Supplementary Fig. S2. Protein alignment of the GS1 family.

Supplementary Fig. S3. Phylogenetic tree of the asparagine synthetase gene family.

Supplementary Fig. S4. Protein alignment of the ASN family.

Supplementary Table S1. Primers used for transcript amplification of *HvGS* and *HvASN* genes by qPCR.

Supplementary Table S2. *GS* and *ASN* genes in *A. thaliana, O. sativa*, and *Z. mays*

Supplementary Table S3. Intron–exon sequences of *HvGS* and *HvASN*.

Supplementary Table S4. Per cent identity matrix: glutamine synthetase proteins.

Supplementary Table S5. Per cent identity matrix: asparagine synthetase proteins.

Supplementary Annex S1. Supplementary materials and methods.

Supplementary Data
