# Supplementary material for: The identification of new cytosolic glutamine synthetase and asparagine synthetase genes in barley (Hordeum vulgare L.), and their expression during leaf senescence
Source: J Exp Bot. 2015 Feb 19;66(7):2013–26. doi: 10.1093/jxb/erv003 (PMC4378633; doi:10.1093/jxb/erv003)
Supplement: Supplementary Data [file supp_66_7_2013__index.html]

The identification of new cytosolic glutamine synthetase and asparagine synthetase genes in barley (Hordeum vulgare L.), and their expression during leaf senescence — The identification of new cytosolic glutamine synthetase and asparagine synthetase genes in barley (Hordeum vulgare L.), and their expression during leaf senescence — Supplementary Data 

# The identification of new cytosolic glutamine synthetase and asparagine synthetase genes in barley (Hordeum vulgare L.), and their expression during leaf senescence

## Supplementary Data

Data files

**Files in this Data Supplement:**

- Supplementary Data - Supplementary Data
- Supplementary Data - Supplementary Data
