# Supplementary material for: The identification of new cytosolic glutamine synthetase and asparagine synthetase genes in barley (Hordeum vulgare L.), and their expression during leaf senescence
Source: J Exp Bot. 2015 Feb 19;66(7):2013–26. doi: 10.1093/jxb/erv003 (PMC4378633; doi:10.1093/jxb/erv003)
Supplement: Supplementary Data [file supp_erv003_jexbot132365_file002.pdf]

Annex 1: Complement to methods.

Cytosolic glutamine synthetase and asparagine synthetase genes are differentially regulated by ageing, nitrogen availability and dark induced senescence in barley (*Hordeum vulgare* L.)

Liliana Avila-Ospina, Gilles Clément, Anne Marmagne, Joël Talbotec, Karin Krupinska and Céline Masclaux-Daubresse

Plant growth

In nitrogen stress experiments, barley (Hordeum Vulgare L.) Cultivar Golden promise, a two-rowed spring barley cultivar was used due to its routine usage in biotechnological applications. Plants were grown in a growth chamber with controlled photoperiod, temperature and humidity (16h – 25°C/ 8h – 17°C). Seeds were sown on a seedbed and five days seedlings were transferred into polyvinyl chloride (PVC) tubes containing sand as a substrate. The experimental unit was a tube (6 ø – 45 cm units) containing 3 seedlings. Plants were watered eight times per day with a nutritive solution containing 5 mM NO3- (124 mM KH2PO4, MgSO4 ; 19.95 mM KNO3 ; 2.49 mM CaN2O6, 1mM NaCl ; 0.04 μM (NH4)6Mo7O24, 24.3 μM H3BO3, 11.8 μM MnSO4, 3.48 μM ZnSO4, 1 μM CuSO4 ; 0.001% Sequestrene 138 FE 100 Syngenta) named high nitrate treatment (HN) or a 0.5 mM NO3- (124 mM KH2PO4, K2SO4, MgSO4, KNO3 ; 0.625 mM CaN2O6, CaCl2 ; 0.04 μM (NH4)6Mo7O24, 24.3 μM H3BO3, 11.8 μM MnSO4, 3.48 μM ZnSO4, 1 μM CuSO4 ; 0.001% Sequestrene 138 FE 100 Syngenta) named low nitrate treatment (LN).

In field experiments, spring barley (Hordeum Vulgare L.) Cultivar Carina was used. The experiments were performed at *Hohenschulen research farm at 15.5 km west of Kiel* during the 2013 growing season, June being nearly wet and July warm and relatively dry. Spring barley was sown using a drill on April 2 of 2013. The barley was managed organically and organic manure equal to 70 kg N ha−1 was added. There were four replicate plots 150 m2 each. Plants were grown in a concentration of 300 plants/m2 with 12.5 cm of row distance. Crop was spreaded with 1.5 L/ha of Ariane C (Dow agrosciences) and 20 g/ha of Trimmer SX (FCS) [herbicides] on May 14 of 2013. Subsequently, it was added 0.3 L/ha of Moddus (Syngenta) and Ethephon (Bayer CropSc.) [growth regulators], 0.5 L/ha of Gladio (Syngenta) [fungicide], 5 kg/ha of MgSO4 and 10 L/ha of Mn-EDTA on June 5 of 2013. At last, 150 kg/ha Kierserit (KALI) [25% MgO, 20% S) and 30 kg/ha KAS (76% NH4NO3, 24% CaCO3] were added on June 7 of 2013.

Chlorophyll measurements adapted from Arnon (1949):

50 μl of crude leaf extract, obtained grinding fresh material in 50 mM Tris-HCl pH7.5 buffer (100 mg FW / 1 ml buffer) were homogenised in 950 μl of Acetone 80% and kept overnight

at 4°C in the dark. Acetone extract was then centrifuged to remove cell debris and absorbance was measured spectrometrically at 652 nm on the whole volume. Calculation is: [mg of chlorophyll / cuvette = 36xDAbsorbance].

Glutamine synthetase essay:

According to O'Neal and Joy (1973)

BUFFERS:	Stock solutions concentration (mM)
Extraction buffer<br>Tris-HCl pH 7.6<br>$MgC1_2$<br>Na-EDTA	 250<br>10<br>10
Reaction buffer<br>Tris-HCl pH 7.6	 50
AMIX (X5) pH 7.6<br>MgS04<br>Glutamate<br>Hydroxylamine<br>EDTA	 150<br>600<br>45<br>30
ATP (X5) pH 7.6	60
STOP buffer<br>FeC13 (28% solution)<br>TCA<br>HCl	 370<br>200<br>1.79 M
Gamma-glutamylhydroxamate	20

AMIX and ATP are prepared in the 50mM Tris-HCl pH7.6 reaction buffer. Gamma-glutamylhydroxamate is dissolved in H20.

150 mg FW are grinded in 1 mL extraction buffer containing 2X protease inhibitor cocktail complete EDTA-free (Roche), 0.5% polyvinyl pyrrolidone (w/v) and 0.1% beta-mercaptoethanol (v/v) added just before extraction.
After homogenisation, extract was centrifuge 13000g for 10 min and supernatant used for protein quantification using Coomassie Protein assay reagent from BioRad, Hercule, California, USA, abd GS assays.

For GS activity measurement in 96-well plates, 50 μl of extract is added to 60 μl of reaction buffer, 20 μl of AMIX and 20 μl of ATP. No ATP is added in blank control; ATP is replaced by reaction buffer.
Standard is obtained with successive dilutions of gamma-glutamylhydroxamate (from 0 to 20 mM).

After 30 min incubation at 30°C and under shaking, 150 μl of STOP solution is added in each well. Plates are then centrifuged (4000g for 10 min) and 200 μl of each well transferred to clean plate. Absorbance is then measured at 540 nm. Activity is expressed as nmol gamma-glutamylhydroxamate formed per min and per mg protein or mg FW.
