# Supplementary material for: The identification of new cytosolic glutamine synthetase and asparagine synthetase genes in barley (Hordeum vulgare L.), and their expression during leaf senescence
Source: J Exp Bot. 2015 Feb 19;66(7):2013–26. doi: 10.1093/jxb/erv003 (PMC4378633; doi:10.1093/jxb/erv003)
Supplement: Supplementary Data [file supp_erv003_jexbot132365_file001.pdf]

# The identification of new cytosolic glutamine synthetase and asparagine synthetase genes in barley (Hordeum vulgare L.), and their expression during leaf senescence

**Liliana Avila-Ospina[1,2], Anne Marmagne[1,2], Joël Talbotec[1,2], Karin Krupinska[3] and Céline Masclaux-Daubresse[1,2,*]**

Supplementary
Journal of Experimental Botany
online

**Supplementary Figure 1. Phylogenetic tree of the cytosolic Glutamine synthetase 1 gene family.** DNA coding sequences (CDS) were translated to protein and then aligned using ClustalW. Species abbreviations are as follows : Hv (*Hordeum vulgare*), Os (*Oryza sativa*), Ta (*Triticum aestivum*), So (*Saccharum officinarum*), Zm (*Zea mays*), At (*Arabidopsis thaliana*), Vv (*Vitis vinifera*), Ps (*Pisum sativum*), Lp (*Lolium perenne*), Sb (*Sorghum bicolor*), Cs (*Chlamydomonas smithii*), Dm (*Drosophila melanogaster*), Hs (*Homo sapiens*), An (*Aspergillus nidulans*), Sf (*Streptomyces filamentosus*), Ec (*Escherichia coli*) and Sa (*Staphylococcus aureus*).

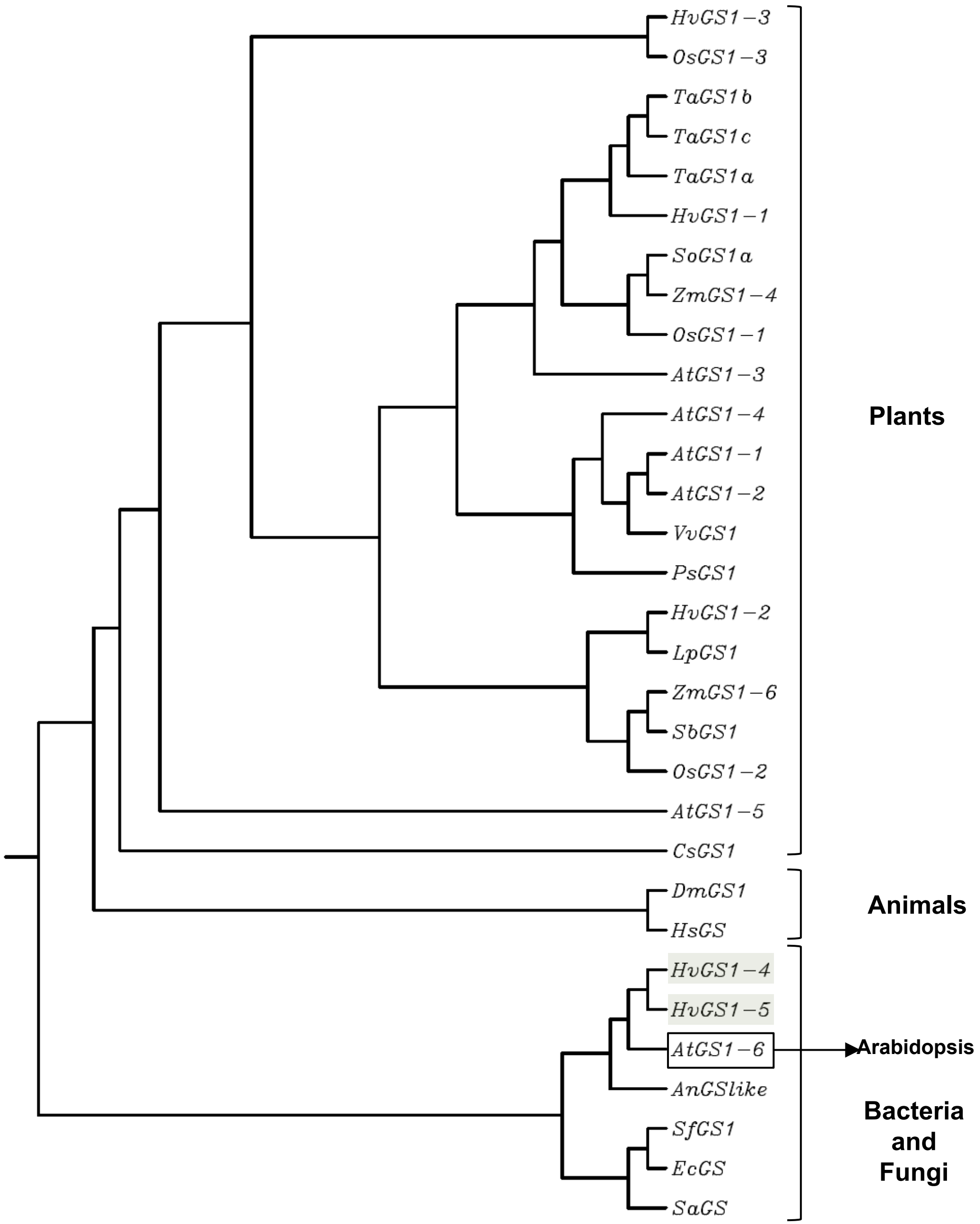

**Supplementary Figure 2. Protein alignment of the GS1 family.** GS proteins of different species including prokaryote and eukaryote organisms were aligned using ClustalW. Conserved amino acids (aa) are shown shaded in blue going from less conserved aa (light blue) to more conserved aa (dark blue). Conserved aa residues found in all species analysed are indicated by black arrowheads (▼) and aa residues responsible for ligand binding specificity in the GS family are indicated by red arrowheads (▼). Species abbreviations are as follows: Hv (*Hordeum vulgare*), Os (*Oryza sativa*), Ta (*Triticum aestivum*), So (*Saccharum officinarum*), Zm (*Zea mays*), At (*Arabidopsis thaliana*), Vv (*Vitis vinifera*), Ps (*Pisum sativum*), Lp (*Lolium perenne*), Sb (*Sorghum bicolor*), Cs (*Chlamydomonas smithii*), Dm (*Drosophila melanogaster*), Hs (*Homo sapiens*), An (*Aspergillus nidulans*), Sf (*Streptomyces filamentosus*), Ec (*Escherichia coli*) and Sa (*Staphylococcus aureus*).

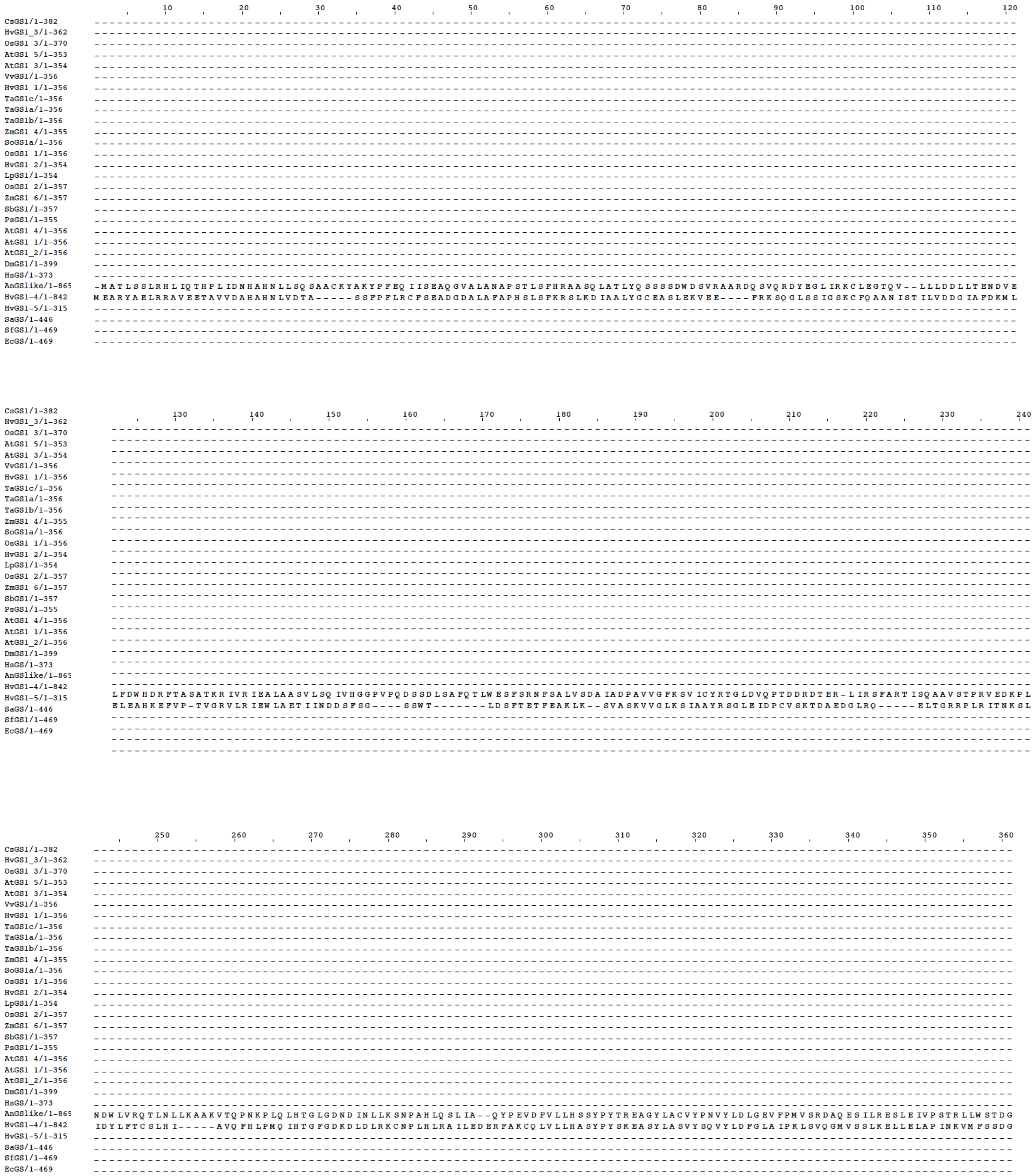

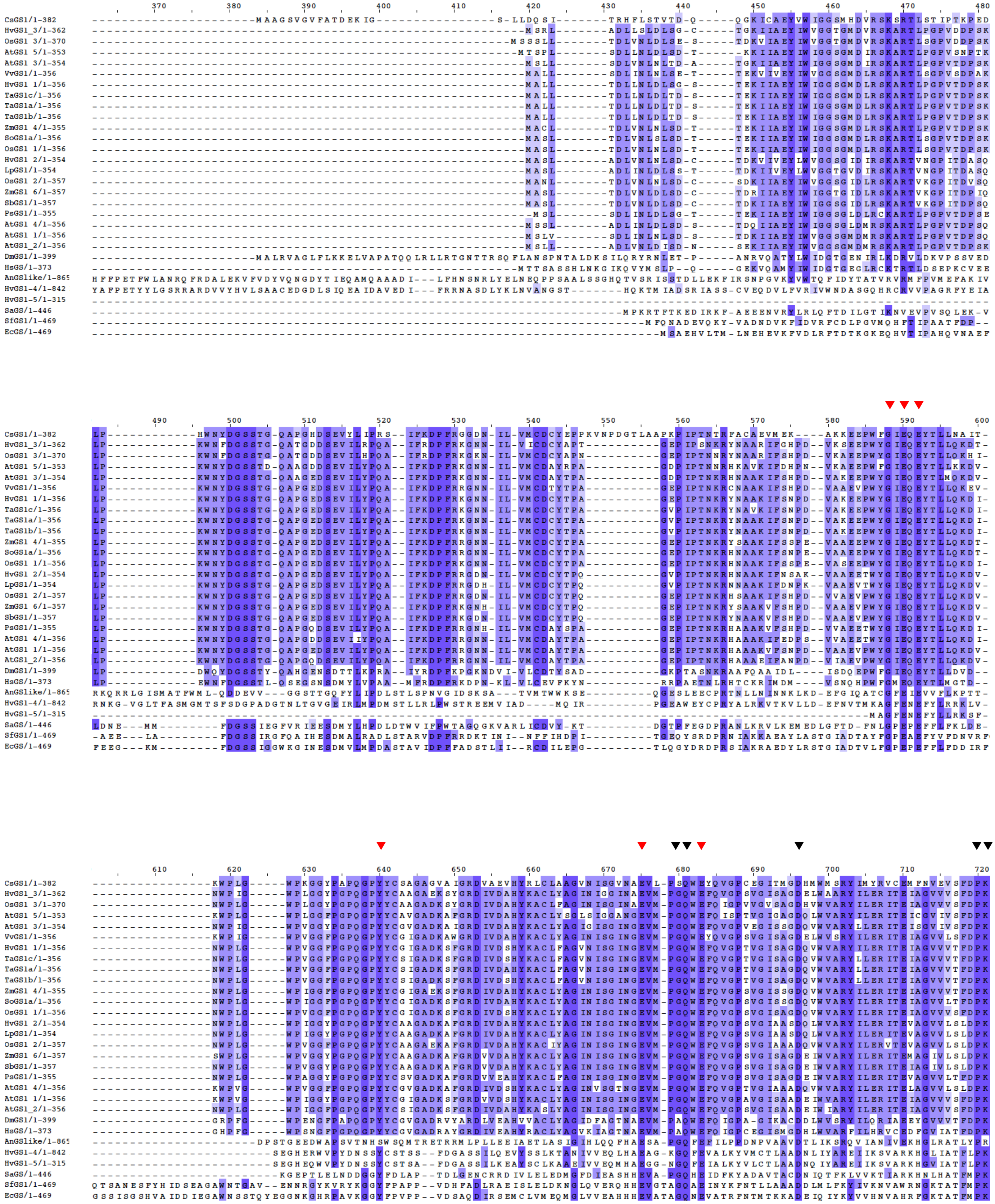

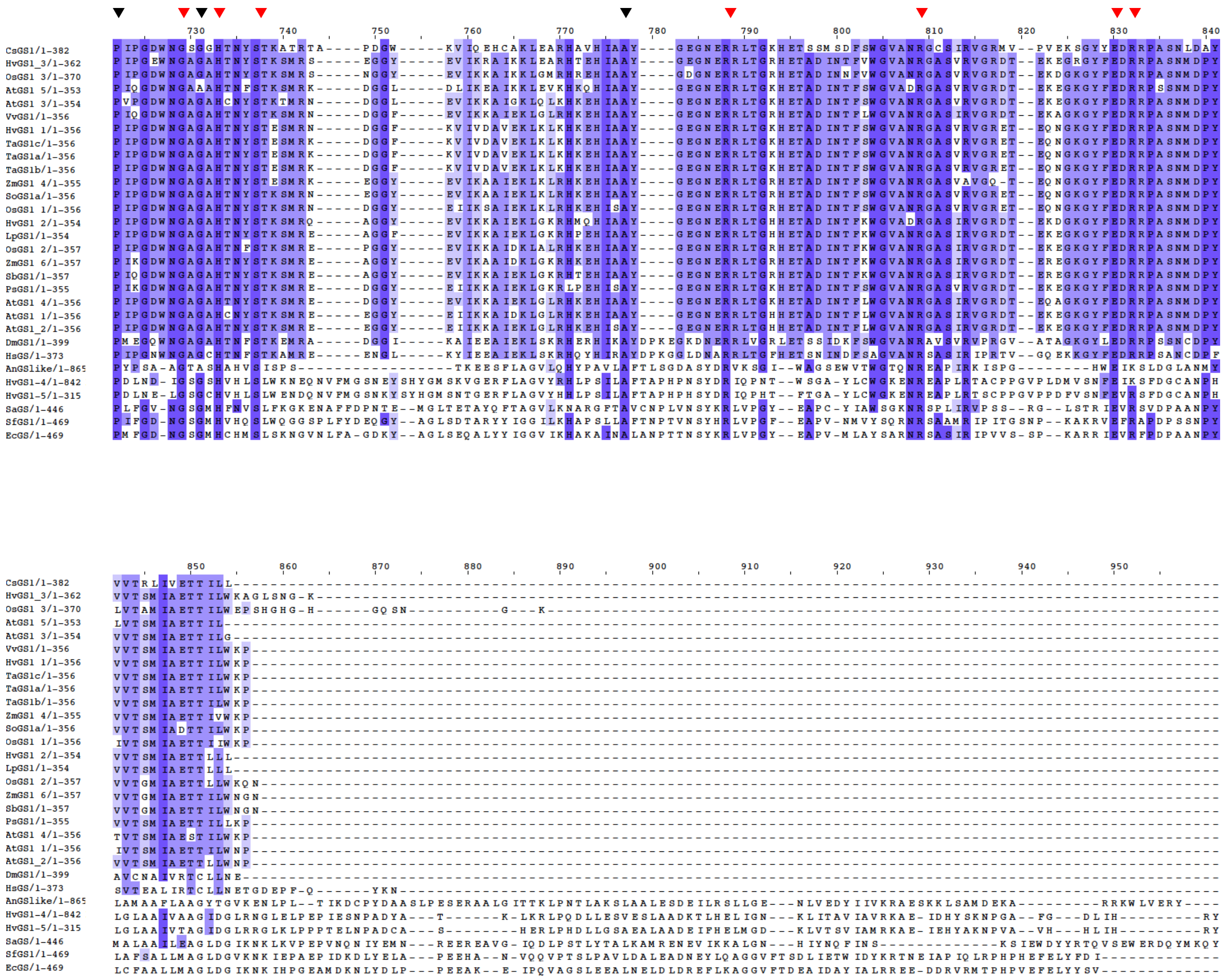

730
740
750
760
770
780
790
800
810
820
830
840
850
860
870
880
890
900
910
920
930
940
950
CsGS1/1-382
HvGS1_3/1-362
OsGS1 3/1-370
AtGS1 5/1-353
AtGS1 3/1-354
VvGS1/1-356
HvGS1 1/1-356
TaGS1c/1-356
TaGS1a/1-356
TaGS1b/1-356
ZmGS1 4/1-355
SoGS1a/1-356
OsGS1 1/1-356
HvGS1 2/1-354
LpGS1/1-354
OsGS1 2/1-357
ZmGS1 6/1-357
SbGS1/1-357
PsGS1/1-355
AtGS1 4/1-356
AtGS1 1/1-356
AtGS1_2/1-356
DmGS1/1-399
HsGS/1-373
AnGSlike/1-865
HvGS1-4/1-842
HvGS1-5/1-315
SaGS/1-446
SfGS1/1-469
EcGS/1-469

**Supplementary Figure 3. Phylogenetic tree of the Asparagine synthetase gene family.** DNA coding sequences (CDS) were translated to protein and then aligned using ClustalW. Species abbreviations are as follows: Hv (*Hordeum vulgare*), Zm (*Zea mays*), Os *(Oryza sativa*), At *(Arabidopsis thaliana*) and Gm (*Glycine max*).

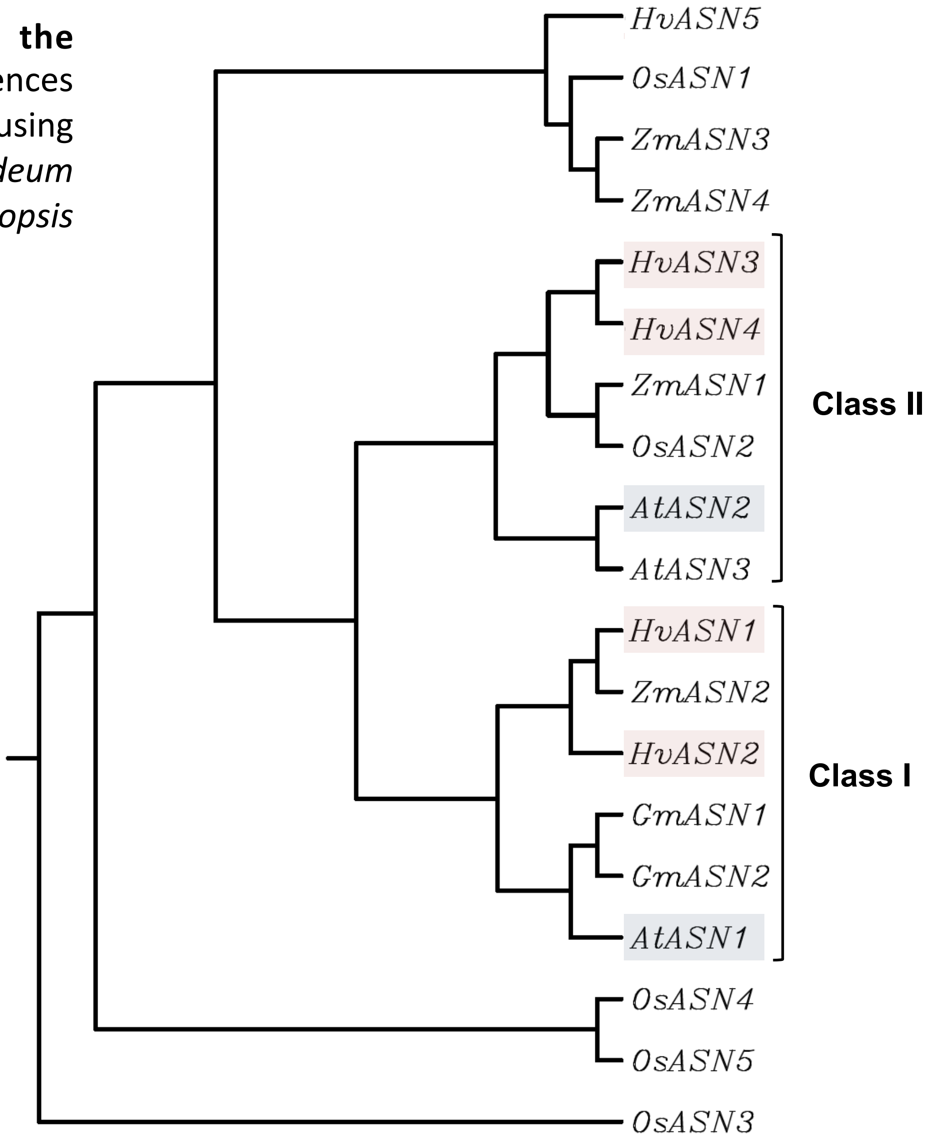

**Supplementary Figure 4.** Protein alignment of the ASN family. ASN proteins of different species were aligned using ClustalW. Conserved amino acids (aa) are shown shaded in blue going from less conserved aa (light blue) to more conserved aa (dark blue). Conserved aa residues found in all species analysed were indicated by orange arrowheads (▼), amino acid residues from the *PurF*-type glutamine binding domain are indicated by purple arrowheads (▼), essential residues for the glutamine binding and positioning are marked by green arrowheads (▼) and amino acid residues proposed to facilitate the binding of aspartate and ATP are indicated by light blue arrowheads (▼). Species abreviation are as follows : Species abreviation are as follows : Hv (*Hordeum vulgare*), Zm (*Zea mays*), Os (*Oryza sativa*), At (*Arabidopsis thaliana*) and Gm (*Glycine max*).

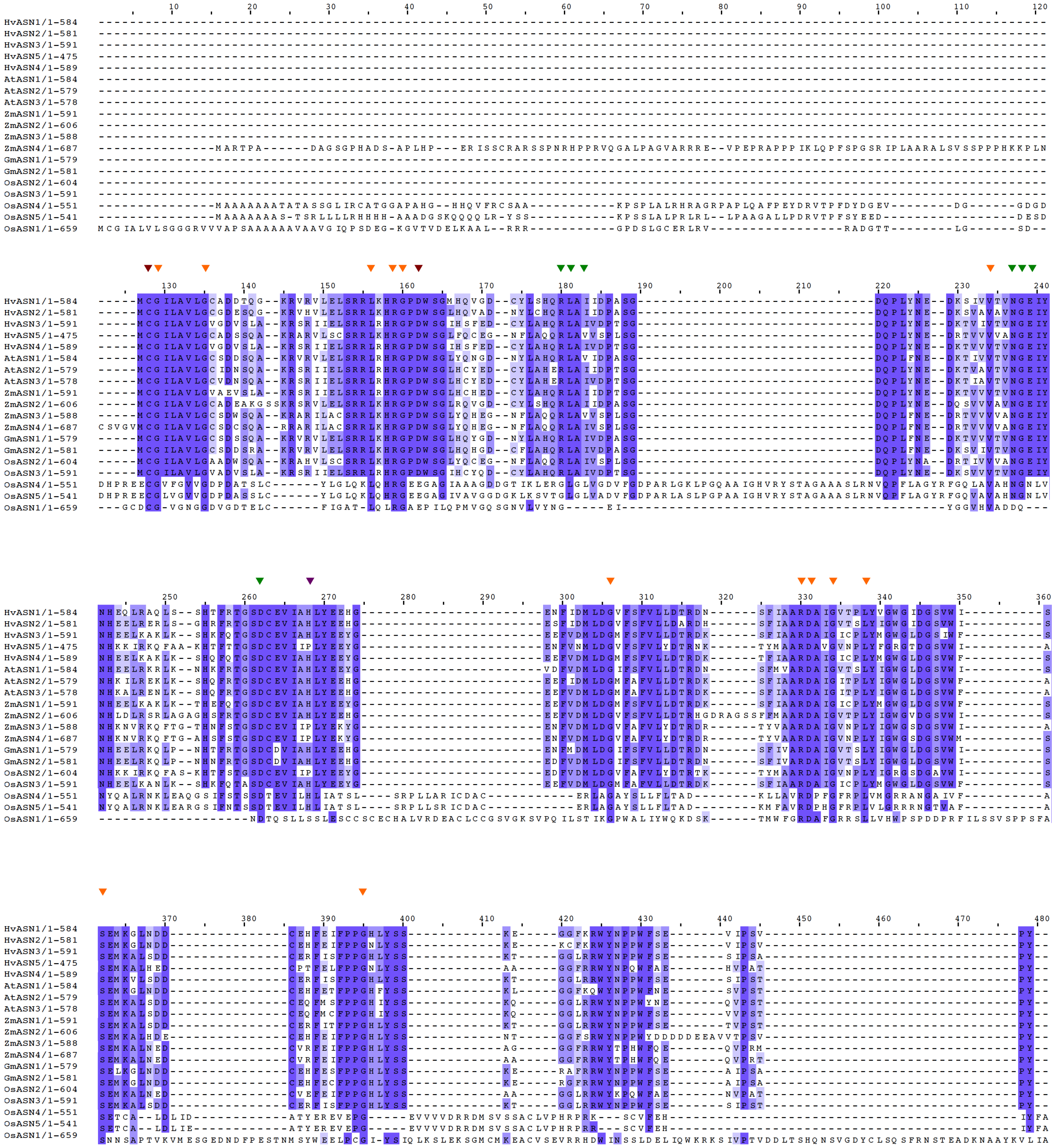

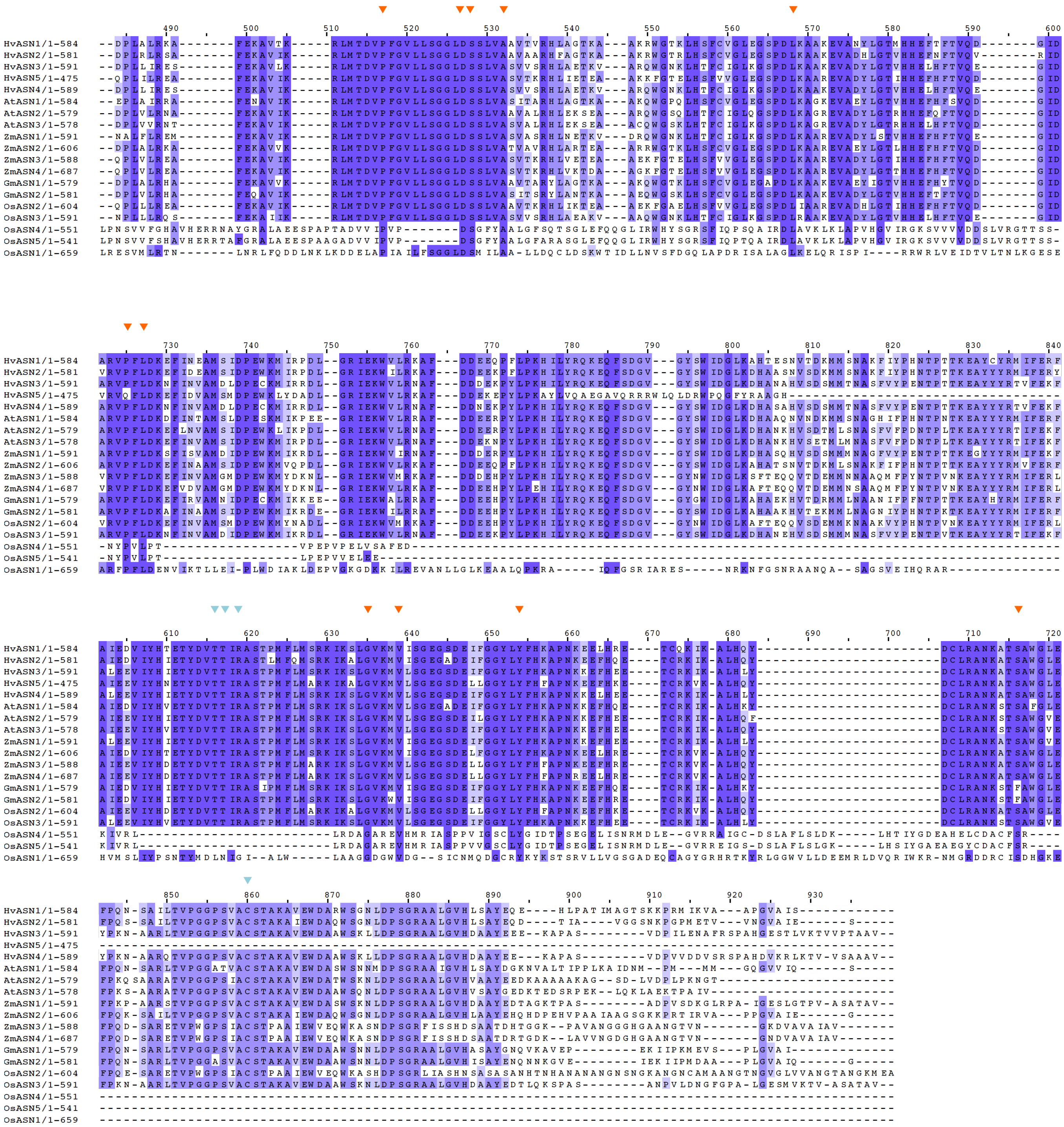

HvASN1/1-584
HvASN2/1-581
HvASN3/1-591
HvASN5/1-475
HvASN4/1-589
AtASN1/1-584
AtASN2/1-579
AtASN3/1-578
ZmASN1/1-591
ZmASN2/1-606
ZmASN3/1-588
ZmASN4/1-687
GmASN1/1-579
GmASN2/1-581
OsASN2/1-604
OsASN3/1-591
OsASN4/1-551
OsASN5/1-541
OsASN1/1-659
490 500 510 520 530 540 550 560 570 580 590 600
610 620 630 640 650 660 670 680 690 700 710 720
730 740 750 760 770 780 790 800 810 820 830 840
850 860 870 880 890 900 910 920 930

**Supplementary Table 1.** Primers used for transcript amplification of *HvGS* and *HvASN* genes by RT-qPCR

| Gene | Accession | Sequence 5'-3' | Source | Amplification size (bp) |
|---|---|---|---|---|
| *HvGS1_1* | JX878489 | Fwd GGACCGTCGGTGATGGGG<br>Rev AAGACGAGAACGAGAAGAGAGACCAGAC | Goodal *et al*, 2013 | 178 |
| *HvGS1_2* | JX878490 | Fwd CACTTTGGGCAGGCTCTCGTCTC<br>Rev CAGACTAGACCTTGCAATTGCAAAAGAAAC | Goodal *et al*, 2013 | 106 |
| *HvGS1_3* | JX878491 | Fwd CTCCAATGGCAAGTAGAGTTACCTGTG<br>Rev TTATTCAAACCTTGCCAGTCTCATCAC | Goodal *et al*, 2013 | 107 |
| *HvGS2* | AK360336 | Fwd AAGCTGGCGCTGAAGGTATGAAGG<br>Rev GACGGAACCACAGGATCAACAAGAATG | Goodal *et al*, 2013 | 124 |
| *HvGS1_4* | AK252215 | Fwd AACGGTTCCTTGCTGGAGTA<br>Rev AAGGCCGTTCCTAAGTCCAT | This work | 284 |
| *HvGS1_5* | AK365395 | Fwd GTTGCTCGAAAACACGGAGT<br>Rev CACATGACAACCGGATCCAA | This work | 78 |
| | | | | |
| *HvASN1* | AK359770 | Fwd ACGGAGAGAGGTCTTCTAGC<br>Rev ATTCACAGTGACGACGATGG | This work | 274 |
| *HvASN2* | AK357350; AK373732 | No primers could be found for this gene | | |
| *HvASN3* | AK353762 | Fwd CAAGAATGCTGCTAGGCTGA<br>Rev GGGTGGAAGGTTAAACAGCA | This work | 266 |
| *HvASN4* | AK363899 | Fwd TGGTTGTAAATATTTCACCGTGG<br>Rev ACCAAATGCTGAGCAACTCAA | This work | 53 |
| *HvASN5* | AK361923 | Fwd CCTCGGACTGATGAGCCA<br>Rev GACGGATCGATCAATACATCACA | This work | 202 |
| | | | | |
| *HvLSU* (Rubisco large subunit) | | Fwd CTCGCGGTATCTTTTTCACTC AGG<br>Rev CGTCCCCAAAGATTTCGGTCAGA | Goodal *et al*, 2013 | |
| *HvSSU* (Rubisco small subunit) | | Fwd CTACCACCGTCGCACCCTTCC<br>Rev TGATCCTTCCGCCATTGCTGAC | Christiansen *et al*, 2011 | |
| *HvNAC13* | AK376297 | Fwd ATGCCGCCGCACATGATGTAC<br>Rev ACAGGTCGCCGGAATTAGCG | Christiansen *et al*, 2011 | |
| *HvActin* | AY145451 | Fwd CGACAATGGAACCGGAATG<br>Rev CCCTTGGCGCATCATCTC | Rapacz *et al*, 2012 | |
| *HvGAPDH* | AAA32956 | Fwd GCTCAAGGGTATCATGGGTTACG<br>Rev GCAATTCCACCCTTAGCATCAAAG | Hebelstrup *et al*, 2010 | 98 |

**Supplementary Table 2. *GS* and *ASN* queries.** Genes used as queries for barley homologs searching

| Gene in *Arabidopsis thaliana* | TAIR locus | No. of amino acid residues | Gene in *Oriza sativa* | TIGR locus | No. of amino acid residues | Gene in *Zea mays* | Gramene locus | No. of amino acid residues |
|---|---|---|---|---|---|---|---|---|
| *AtGS1_1* | At5g37600 | 356 | *OsGS1_1* | Os02g0735200 | 356 | *ZmGS1_1* | GRMZM2G050514 | 357 |
| *AtGS1_2* | At1g66200 | 356 | *OsGS1_2* | Os03g0223400 | 357 | *ZmGS1_2* | GRMZM2G024104 | 368 |
| *AtGS1_3* | At3g17820 | 354 | *OsGS1_3* | Os03g0712800 | 370 | *ZmGS1_3* | GRMZM2G046601 | 357 |
| *AtGS1_4* | At5g16570 | 356 | *OsGS2* | Os04g0659100 | 428 | *ZmGS1_4* | GRMZM5G872068 | 356 |
| *AtGS1_5* | At1g48470 | 353 | | | | *ZmGS1_5* | GRMZM2G036464 | 355 |
| *AtGS1_6* | At3g53180 | 845 | | | | *ZmGS2* | GRMZM2G098290 | 423 |
| *AtGS2* | At5g35630 | 430 | | | | | | |
| | | | | | | | | |
| *AtASN1* | At3g47340 | 584 | *OsASN1* | Os03g18130 | 604 | *ZmASN1* | GRMZM2G074589 | 591 |
| *AtASN2* | At5g10240 | 578 | *OsASN2* | Os06g15420 | 591 | *ZmASN2* | GRMZM2G093175 | 604 |
| *AtASN3* | At5g65010 | 578 | *OsASN3* | Os12g38630 | 561 | *ZmASN3* | GRMZM2G053669 | 588 |
| | | | *OsASN4* | Os01g65260 | 551 | *ZmASN4* | GRMZM2G078472 | 588 |
| | | | *OsASN5* | Os05g35580 | 541 | | | |

**Supplementary Table 3. Intron-Exon sequences of *HvGS* and *HvASN*.** Exon sequences (blue), intron (black) and untranslated regions (red) of five isoforms of *HvGS1, HvGS2* and five isoforms of *HvASN*.

| Gene | Intron/Exon | Lenght | Sequence |
|---|---|---|---|
| ***HvGS1_1*** | 5' upstream sequence | | AGCGCATCCATCCATCCATCCATCCATCCTTGCGTCGCGCGTATCCGGAACGGGGCGGGGGTAATAAATCACCGCCAAAAACCTCTTCCCGTCGCGCTGCCATGCCGGAAACCTAGTGTATTTCCCGACGAGAAAGGTGGCGTTCACCGCCCGCACGAGGGAGCACCGCCGCCCGCCTCGCTGCGCCCGCGGATCGCGAGGGCGAAAAAGCGTCTGATTAAAAATAAAAAAGGAACACTGCTACGACGAATCTGCACCCCCCATGAATTTAGCATCGAGGAAAAACGACCCAGCGCTCCGGGCGATCCCGCCTCCTCCTCTCCCCCCCCC |
| | Exon1 | **113 nt** | CTGGCCTTGCTCCTGAGATCCATGCCAGATCCGCCGATCCATATGTACTCGGCGATGATCTTCTCCGTGGAGCCGGAGAGGTCGAGGTTGAGGAGATCGGTGAGGAGCGCCAT |
| | Intron1 | **236 nt** | TGCACCAACCAGAGCAGCAGCAGCAGCAACAGTTAAAAAATAATACGCACGCTGTTAACCAAAAGGAATCTGGCAACCTCTGATATATAAGCACAAGAACAGCTCAGTGGTGGATAACTAGCTAGCAAAAAGGTGATAGGACACGGCAAGCAAGGTCATGGGAATGATCCATGCCATGCCGGCCGGGGAGGGGAATCTTTATCTGTATATGTATGCCTCTGTATATGCATCTTACC |
| | Exon2 | **105** | TACAGGATGACCTCGCTGTCCTCGCCCGGGGCCTGGCCGGTGCTGGAGCCGTCGTAGTTCCACTTGGGCAGCTTGCTGGGGTCGGTGACCGGGCCGGGGAGGGTC |
| | Intron2 | **133 nt** | CTGCAGCATGGCACATGCCCCAAGGGAAGCAAAAATTACTCCTCGGGTCCAAGGTTAAAAAAAAAAAGGTGATACTAGAATTGCACTTGACAGAGGTAAATAGTTCTGGGGTGGCACGAGAGACGGCACTTAC |
| | Exon3 | **49 nt** | AAGGATGTTGTTTCCCTTCCTGAACGGGTCCTTGAAGATGGCCTGTGGG |
| | Intron3 | **450 nt** | CTGCAAGTGGTTCGAATTTAGTAAGGGGGGATCTTGATTGGCATCAAGGATGTTGACAAGATGATGACCATTGGATTCCCTCTCTACTGTAGTTATTAGGTGGACCAGACAATCACACCTAATTAATAACCAAAAATACGTATAACAATTCATTCACAACCCTGGATGGTCCTTGACTGGGATACAATTTGCGTCAGAAATAGGAGTATCAACAGCTAGTATGAATACTGCACCAAAAAAATCTGCACCAAGTAGTACATGAGAAGATAGTTAAGTTGATCTCCCCTCTTAATGGTGCTCGTTAATCTTCGTAGTTGACCAAAAGAGCTATAGATCAATCGACTACAGTGAAAAACACTCCTACTAGTACTACAGGTGCAGATCACTTGCGCGGCCAGCAGTGCTACAGTCTATTGTACACACGATGATGATAGTGGAGTGGTCACTTAC |
| | Exon4 | **160 nt** | ATGGCTCCTCCTTGGCAACATCGGGGTTGCTAAAGATCTTAGCAGCGTTGTATCTCTTGTTGGTGGGGATTGGCTCTCCAGCTGGGGTGTAGCAATCGCACATGAC |
| | Intron4 | **112 nt** | TATCATCACAAACAATAGAAAGACCAGTCGATCAATTATGATCATAAAAACAGTTAGACCATTATTCTTTAGCGTTCGTAATCATGGATCAATTTCGTATATTATATATACC |
| | Exon5 | **87 nt** | GAGGACCAGGGAAGCCACCAACAGGCCAGCCGAGAGGCCAGTTGATGTCCTTCTGTAGGAGGGTGTACTCCTGCTCAATACCGTACC |
| | Intron5 | **96 nt** | ACACACAAAAAAAGTTGTGATTGGTAAGTGTACACTTCACCAAAATTAGGGCCAAATGTCCAAATTCAGCCGGTCAAGCTGAAAGAGACGTACCTG |
| | Exon6 | **131 nt** | CTGTCCGGGCATGACCTCGCCGTTGATGCCACTGATGTTGACGCCGGCAAAGAGGCAAGCCTTGTAGTGGGAGTCAACTATGTCACGCCCAAACGACTTGTCAGCACCAATACCACAGTAGTAAGGACCCT |
| | Intron6 | **100 nt** | CTGAAAACCAAACATAATAATTCTACTATGGTCAGAAAGACGGTGCTAATTCTGCAAGATACAGAAAATAGGAATGTTCTGCGTTGTAGTGGTGTTTCAC |
| | Exon7 | **75 nt** | CTCAAGAATGTAGCGAGCGACCCACACTTGGTCACCAGCAGAAATGCCAACAGTCGGGCCAACTTGGAACTCCCA |
| | Intron7 | **148 nt** | CTGAAAAACAAAAGGGAAAGTGACATTTAATCACGAGAATTCTAACCGAATGCTAAAGCTATTTGAGCTGTCATTCAACCACAAGGGCCTTATCAGGATAAGATCACATGACTATTCTAGATCATCTTTAAATGAAGACTATCATTAC |
| | Exon8 | **89 nt** | TAGTTCGTGTGAGCACCAGCACCGTTCCAGTCGCCTGGGATGGGCTTGGGGTCAAACGTGACGACAACTCCGGCGATCTCGGTGATCCT |
| | Intron8 | **92 nt** | GATGCAGCAGAGTCGTTCGCCGTCAGCAAAATAGCACGGTAAGATGAACGGATCATATTCATCAGTTCGAGTATCTCAATCTCAATTACCTG |
| | Exon9 | **162 nt** | CAGCTGAAGGTGTTGATGTCGGCCGTCTCGTGCTTGCCGGTCAGACGGCGCTCGTTGCCCTCGCCGTAGGCCGCGATGTGCTCCTTGTGCTTCAGCTTGAGCTTCTCGACCGCGTCCACGATGACCTTGAACCCACCGTCATTCCTCATCGACTCGGTACTG |
| | Intron9 | **96 nt** | TGCAGAGCAAGCAACACGTTTTTCATTTTAGAACCACTGTTGGAGTAACGAGATTCAGGTGATTCAGGAGCAAGAAGGCGAAGGTGGGGGCGTACC |
| | Exon10 | **154 nt** | TCAGGGCTTCCACAGGATGGTGGTCTCGGCGATCATGGAGGTGACCACGTAGGGGTCCATGTTGGACGCCGGCCGGCGGTCCTCGAAGTAGCCCTTGCCGTTCTGCTCCGTCTCCCGGCCCACGCGCACCGACGCGCCACGGTTTGCCACACCC |
| | 3' downstream sequence | | CGGCTGCGGTACGGACCATTTTATTACCATATCAATCGATGATTACACGAGACCATAGCATCCAGACGGCAAGGGAAAACAATCAACCAAATACTGTAGTAATCTTGTGCTTTGACACGCCCCTTGCCACCCATCACCCACCACCCACCCAAAGACGAGAACGAGAAGAGAGACCAGACACCCTGATGAGATGACCATGATGATGTGACCTACCTAAGCGGACTAGGAGTAGTATGCAACCAAACGTAAAAGGAAAATGCTGGAAGAATTTTCGCCCAAGGCACGAATCCTCCAATGGCCACCACCGGACCCCATCACCGACGGTCCATCACACGGCGATCGGAGCT |
| | | | |
| ***HvGS1_2*** | 5' upstream sequence | | **GAGCTGCACACCTCATCTCATCATCGTCTTCCCCCCATTGCCATCGACCTCCCTCCCTGCGAGCAGCAGCAGCAGCA** |
| | Exon1 | **279 nt** | **TTACCAATCACAATCTTACACATCAGTTTGTACGTAAAATCTTTACGTAAGCCTTTGTAGGTGTAGCATTACTCGTACCAAAAAGGGTTGAAGGACCACATAATTTGGAGGTTTTCCACCTTTGTATCCCTGTCCTACCTTATCTGCTTACGTTAAAGCACTGATTGGGCTTATCTCTGAATCCAAACAGGTATGGTATTGAGCAGGAGTACACACTCCTCCAGAAGGATGTGAACTGGCCTCTTGGCTGGCCAATTGGTGGCTACCCTGGTCCTCAGG** |
| | Intron1 | **183 nt** | **CAAGAAAATTCTCAGCATGCTCTCCAGCTCTTTATGTTTTAAGCTTACATAGAAACATAATAACTGAACATTAAGAAAAAAAAAATCAGAGTCCTCTGAAGATAGTAGGTTCCAGACTGAGCAATAAGAGAGGAAGAACCATACTGAATTACTGATCATGTTGATTCTATGCATTTGCGCAGG** |
| | Exon2 | **128 nt** | GACCATACTACTGCGCCGCCGGTGCCGACAAGGCGTTCGGGCGTGACATCGTGGACGCCCACTACAAGGCGTGCCTCTACGCCGGGATCAACATCAGCGGCATCAACGGGGAGGTCATGCCCGGCCAG |
| | Intron2 | **91 nt** | GTACTAGAGTCTAGAGATGATCGATCCTCCTCCTGCAACAAGTTGTATAAAAACTCTCTCTGAACCTGATTCTCTGTCCCCTTGGTGACAG |
| | Exon3 | **73 nt** | TGGGAGTTCCAAGTTGGGCCGTCCGTCGGGATCGCCGCCTCCGACCAGCTGTGGGTGGCGCGCTACATCCTCG |
| | Intron3 | **115 nt** | AGGTCCTCCCCTGATACGTAGTGTACTGTAGTATTATTCGCACCTCGTATCGCAACTGCGTGAATCTGGTTAGATGCTGAACTGATTTATTGGATTGGTTGATCGGTTGATCCTC |
| | Exon4 | **255 nt** | AGAGGATCACAGAGGTTGCCGGGGTGGTGCTGTCCCTGGACCCGAAGCCGATCCCGGGTGACTGGAACGGCGCGGGCGCGCACACCAACTACAGCACCAAGTCCATGAGGCAGGCCGGCGGCTACGAGGTGATCAAGAAGGCCATCGAGAAGCTTGGCAAGCGCCACATGCAGCACATCGCCGCCTACGGCGAGGGCAACGAGCGCCGCCTCACCGGCCACCACGAGACCGCCGA CATCAACACCTTCAAATGGG |
| | Intron4 | **104 nt** | TACGTGCTGCCACATCTGTTGTACTACTGTGTTGCCTGTCGTGTCTGACTCTTACCTGACGATGATGAATCGATGATGATGATGATGATGATGCATTTAGG |
| | Exon5 | **146 nt** | GCGTGGCGGACCGCGGCGCGTCCATCCGCGTGGGGCGCGACACGGAGAAGGACGGCAAGGGCTACTTCGAGGACCGCAGGCCGGCCTCCAACATGGACCCCTACGTCGTCACCTCCATGATCGCCGAGACCACGCTTCTCCTCTGA |
| | 3' downstream sequence | | GCACACGGCCGGCAATGCCTACTCCACCGCCAGATGACACTTTGGGCAGGCTCTCGTCTCGACTCTCTCGATCGAGGGTGGTGATTGATTTCTGCAAAACGTTTCCCGTTTCCATTTCTTTTGCAATTGCAAGGTCTAGTCTGTTTTTGGGGCGTGCCTTTGGTATCTTTCATAGTAGTACGTCTACTGCTCTTCAGGATAAGAAGAGTCTTCAGTGTACTCTGAAAATAATGTTGTTTCCGCATTCTGATAAAATGGAATCATGGAACCGGTTGTGATTCTGTCTGTTCCATCACATGTCATACACACATACAGTACACGGTTCCATGTTCTCCAGTGCTCTTTCCACGTCACCCTGAGCCTGAGGCGAACAAAATTGACGTGCGGGCGCGAAAGCGGATGCGCCAGTTGCAATTGCCTCTCTGCATTTCGAGCAAGTGTCGTGTCACTAGCTGCGTGCGGCCATGCTGCCTTATGTGTTGCAACTTGTTGCAGCCTAATCCGTGGGCCAAACGCATCTTATCTTATCTTTCCTCACTCGCTCACGCGACAACTCGACGAATATGATCTCTGGCCCTCTTTGACGTTGGGCCATTCTTTTTTACGCACACCTTTCCGGACGCAGCGTACCGC |
| | | | |
| ***HvGS1_3*** | 5' upstream sequence | | **GCGAGCTACCCCTCCTCCTCCTCCTTCTTCTNNNNNNNNNNNNNNNNNNNNNNNNCTCCTCTTCTTCTTCTTCTTCCCCGGAGACGGAGCCTGAGCCGGAGAGGGAGGGGGAGAGCAGGCGGAGGAGGAGGGAGAGGTGTTCTTGTGGGAGGCAAATGGTGGCTAGCTCTCTTGGAAATGCGGGGGGAGGAGGAGGGGCGAGTGGGGGTGGTGTCGCCGTAGCTGGCTCTGCCCCTCTATAAGTAGGTAGGAAGGGAGCAGGGTGGTGACGGGGCCCCCGAAAGGTTGGTGGGCGTTGCGGCCGGGCTGCGTACGTACGGCTGTCCCTCCGCGCGTACGTTTCCGCGGCCCGCGCGCACGCAACGCCTTTTCCACTGCACCACTGCACCGGACGCGAGCGCACTCGACCGGTCGGGTTTCCTCCCTGTCATGTGATGTGATGTGATGTGATGATCC** |
| | Exon1 | **73 nt** | **ATATGTACTCGGCGATGATCTTGCCGGTGCAGCCGGACAGGTCGAGGCTGAGAAGGTCGGCGAGCCGAGACAT** |
| | Intron1 | **402 nt** | **TGAGTTAGGCAAAGCGTAAATGTGAGCAGATTTTCTTGTTTTTGTTCTGAGGGAGTAAATGTGCGCAGATTAGTAATGGCTTTTTTCTTCCTCTTTTGGAAAATGGATGCTGATCGGTGCTATTGCTTCTGTAACTTATCATCAAGACGTGAAGAAGGTGAAGACAGGGGTTTTTACTTGGCTCAAATCTGAGAAACCCAACCAGAAGGAAATTTCAGATGGGATTTGTGGGGAGGAGGAATAATCTGTGGCATGCCTAACTCGGGATAAGAACTAGAGATTTCAAGAAGCGGAAAAGAAAAGGATGAAGTGGATTTTGGTGTGAACTGTGAAGGAAGGTGAGATGGGAGGTTGGGACAGTGGTGAGGGAGGCGGAGGAAGAGGAAGCCGAGCGGTGTTACC** |
| | Exon2 | **40 nt** | **CTGGCTTTGCTCCTGACGTCCATCCCGGTGCCGCCGACCC** |
| | Intron2 | **153 nt** | **TGATTTGTGAACGTCCAAGGGATCCAAAGGGGCAGAAAAAGTGTTAGGAACTTGCTACTACTACTTGCAATGTATTTTTTTAGAGTGATGGTGATTCTCTTTAGGGTAAGGATTAAATGGATGAT TATGAGGGTGGGAGGAACTCGGCCTACC** |
| | Exon3 | **104 nt** | **GGAGGATGACTTCGCTGTCGTCGCCCGTGGCTTGGCCGGTGCTGGAGCCGTCGAAATTCCACTTTGGAAGCTTGCTGGGGTCGTCCACGGGTCCGGGAAGCGTC** |
| | Intron3 | **145 nt** | **TTGGCAGAAGCAAAAAACATGGAGTTCAAATCAGTTTGACCTCAGGCCCAAGGCACGCAAGCCAAGGCAGGGGTTGATTGAAGAGTGCAACCAAGAAGAAGTGCCGGAGGATTATCCTCAAGAAAAAAGGAAAAAAGGTAGTACC** |
| | Exon4 | **49 nt** | **AGGATGTTGTTCCCTTTCCTGAACGGGTCCCTGAAGATGGCTTGGGGTC** |
| | Intron4 | **169 nt** | **TAAAATTTACCATAGGTGAAAAAACAGTCTGGCATTCATGAACAAATTCCAAAGCTGATTCACGCAGACAAAATAAAATAACAATACAAAACTCATGCCATAACCTTTGTGTGTATATATATATCGTATTTATGAATGAAAAAACAGAGAGGAGGATCCTGAATTTACC** |
| | Exon5 | **107 nt** | **ATGGTTCTTCAGACTTGACATCAGGATGGCCGAATATCCTCGCCGCGTTGTACCGCTTGTTGCTCGGAATCGGCTCTCCGGTAGGCGCATAGCAGTCACAGATGACC** |
| | Intron5 | **110 nt** | **TATATTTCAGGCAACATAAGAGAGGGGGTACAAGATTAATTGCCACATAAATTATGTGCAAAGTTAATTACTTGACTAATTTCTATAGAACGGAGCAACTGATACTGACC** |
| | Exon6 | **86 nt** | **AGGGCCAGGGTAACCCCCTAGTGGCCAGCCAATGGGCCAGTTGGTGTCCTTCTGGAGAAGGGTGTACTCCTGCTCAATCCCATACC** |
| | Intron6 | **129 nt** | **CCACGCACATTTCATCGTTCATCACTTTACTGAAGTATGATGGGCAAGCATTTTTGACAGCAAGTAATTAGCTGAATTTTAGCATTTGCAACTGAAATGCTGAGTATTGAACATATCTATAGTT ACCTG** |
| | Exon7 | **131 nt** | TGCCCTGGCATGACTTCTGCATTGATGCCGCCGATGTTGATGCCGGCGTAGAGGC AGGCCTTGTAGTGGGCGTCGACGATGTCGCGCCCGTAAGATTTCTCCGCACCCGCGGCGCAGTAGTAAGGCCCCTG |
| | Intron7 | **105 nt** | TGGTTGGTTCAGACAGAAGATCATCAGCCCATGAGCATATTTTTTCATGGACAAAGGGGC ATCCAAATATGTGAATGTGATCCTGCGCCTGAACCTGAAATTACC |
| | Exon8 | **75 nt** | TCGAGAATGTAGCGAGCCGCCCAGAGCTCGTCGCCGGCGGAGATGCCGACGGAAGGGCCGACTTGGAACTCCCAC |
| | Intron8 | **94 nt** | TGTTGGGTCACACACAAAACAAAGATGTTCTTATCAGCAAAAGTTGCATTTGATTTGATTCATTCAACTAGACGAGGCAGTTTTACAATTTACC |
| | Exon9 | **56 nt** | CCGGGATCGGTTTGGGGTCGAAGGAGACGACGACGCCGGCGATCTCAGTGATCCTC |
| | Intron9 | **88 nt** | CCTGCAATCACAAACATGTAAGAAATGGCTAGCTGGCCAGTGCAGATCAAGTTGAGAATTTTGGTACAACAAG ATGAAGAAATGCTCA |

| | | | |
|---|---|---|---|
| | Exon10 | **34 nt** | TAGTTTGTGTGGGCACCGGCACCGTTCCACTCTC |
| | Intron10 | **84 nt** | CATTCAGACATCATCAGGATCATGCCATGACCCATAGCACAAGAGTAGCGATGCATTAGTTTCGCTTGCTCTTTAGATTACCTG |
| | Exon11 | **162 nt** | CATACGAAGGTGTTGATGTCGGCGGTCTCGTGGCGGCCGGTGAGCCGGCGCTCGTTGCCTTCCCCGTAGGCGGCTATGTGCTCCGTGTGCCGCGCCTCGAGCTTCTTGATCGCCCTCTTGATCACCTCGTACCCGCCCTCGCTCCTCATCGAC TTGGTGCTG |
| | Intron11 | **101 nt** | TGGAAGAAACAAGAAAAACAAACAATGTAGACGGAAAAGCAGTAAGAAGAGAGCAATGGTGACGCTCTTGTCTCAAGTCTCAACTCGCCGTATTACTTACC |
| | Exon12 | **172 nt** | CTACTTGCCATTGGAGAGACCGGCCTTCCAGAGGATGGTGGTCTCGGCGATCATGGAGGTGACGACGTAGGGATCCATGTTGGACGCCGGCCTCCGGTCCTCGAAGTAGCCCCTGCCTTCCTTCTC GGTGTCGCGCCCCACCCGCACCGACGCGCCGCGGTTTGCCACGCCC |
| | 3' downstream sequence | | CGTTGCCTATACTGCATACTCTGCTGTGCTGCCGGTACAGTATACGGCCGTAATCTTGTCACAGTGATTGTAATCAAGAGTGCGTTTCTTCATGCAATATATTGACGTGTTCCTTTTTTTTGCCGAGGATTCGTGTACCAATCTTATCTTGAACATCCAAAAGGAAAACATGAAAAACGTAGCAGGAGTTTCTGAAGGATATCATTTCGTGTAGGAAATGTTTCTTTGCTACAGTTTGGAACAGCGGAATTACCATCTTACTTTCCTTTGAAATTCCCTGTGGAATGGGCTACTCCATGGGAACTTTGGAGGAAACCAAATGAATCCTTGTGTTTACAATGGTTTGTCTTGCGATTGAAGAAGACCACAATTAAGTGCCAAACGTAGGTGCCATGACCGCATAACACGAGATGTTTGCAGAAGCGGAACATACATCCAGCAACGGCAAGCGCATCACATCACATCAGTTATTGCTTCATCATTTTTATTCAAACCTTGCCAGTCTCATCACTGCAATGCAGATCACGCACACACAACCACGGGGCGCTTACACATAACACCATCACAGGTAACT |
| | | | |
| | 5' upstream sequence | | GGTCGCCCCCCCTTCCCTCCCTCGCCTCGCCCGCGTCGCCGTCTCTCTGGTTTAGGGGCCGCGGAGTCGCTGTACGTAAGTAAGTAAGTACGTAGAGACG |
| | Exon1 | **239 nt** | ATGGCGCAGGCGGTTGTGCAGGCGATGCAGTGCCAGGTGGGGGTGAGGGGCAGGACGGCCGTCCCGGCGAGGCAGCCCGCGGGCAGGGTGTGGGCGTCAGGAGGGCCGCCCGCGCCACCTCCGGGTTCAAGGTGCTGGCGCTCGGCCCGGAGACCACCGGGGTCATCCAGAGGATGCAGCAGCTGCTCGACATGGACACCACGCCCTTCACCGACAAGATCATCGCCGAGTACATCTG |
| | Intron1 | **380 nt** | GTACGGTACGCCCGCCGTTGTTTCCGAGTCATATTAGCAGCTTAGGATAGGCCAAACCTGCACTTTTAGGGGATTGCACTAGGTTTGATTAGATTAGCACGTGAGCGAGCACTGCAGTGTGGATTTGCGCCCTTAAAGTGGATATACACTGCTAATAATAGATAAAGCTTCCAAAGGCGTGATGGGTGATTAGTGATTAGTGACGACTCACTGCTTTTGCCTTTGTAGTTCCGGCACTTGAATAAGATTTTGGGCAGTGGACACCCCACTTTTGGGGTGCAACCAGGAATAGATCCTGTCATGTCATGGCACGTACCACTATCATGTAGTCGCATTACTATTAATTAACAAATCTGACTTTCTTTCTTCACACTGTGCAG |
| | Exon2 | **40 nt** | GGTTGGAGGATCTGGAATTGACCTCAGAAGCAAATCAAGG |
| | Intron2 | **140 nt** | GTAAGATCTTCATGGCCATATCGCTGTTTCCTTCCATACATACTACTACATCACACTTGTCTCTGGAGTTGACTGCAGAGCATTGTTTCTGTACTTTCTATTTCTGCCACTGACAAGAAAATGCAATGCTACTGTTTCAG |
| | Exon3 | **104 nt** | ACGATTTCGAAGCCAGTGGAGGACCCGTCAGAGCTGCCGAAATGGAACTACGACGGATCGAGCACGGGGCAGGCTCCTGGGGAAGACAGTGAAGTCATCCTATA |
| | Intron3 | **114 nt** | GTAAGGGGACAATTACAGTTCATGTGTTCTTCAGCCTTGCACACACAGTCTAATACCGTAATCTCGATATCGACTTTGGCTGACTCTGTTCATGTTTTTCTGTCCTGCTTTCAG |
| | Exon4 | **49 nt** | CCCACAGGCCATATTCAAGGACCCATTCCGAGGAGGCAACAACATACTG |
| | Intron4 | **123 nt** | GTACCTTTCTTCTGGATGTGCTTTATGCTAATCATGAAGAAGTGATTAGTAGTGCTAGTGGTCACTTTACTGGTTACACATATACAATTGGCGTGTTAGTTAATACGGAATTTGTTTTTTCAG |
| | Exon5 | **109 nt** | GTTATCTGTGACACCTACACACCACAGGGGGAACCCATCCCTACTAACAAACGCCACATGGCTGCACAAATCTTCAGTGACCCCAAGGTCACTTCACAAGTGCCATGGT |
| | Intron5 | **95 nt** | AACTACGCATCGAGTACCCGTGCTATATAAAATCCTGCTATTTTCTGATCTCGCTTTTGTTTGCATACGAAACTGAAACATTTGCTTTTCTAGGT |
| | Exon6 | **87 nt** | TCGGAATCGAACAGGAGTACACTCTGATGCAGAGGGATGTGAACTGGCCTCTTGGCTGGCCTGTTGGAGGGTACCCTGGCCCCCAGG |
| | Intron6 | **112 nt** | TACTGCATCGAGAAGCTTGATTTATCAGATGACGATAAACCTTAGGCTGACTGAAATGTACACTTCATTGAACAACAACCAAGTAGTAATATTTGTGCTCAAATGTTGCAGG |
| | Exon7 | **128 nt** | GTCCATACTACTGCGCCGTAGGATCAGACAAGTCATTTGGCCGTGACATATCAGATGCTCACTACAAGGCGTGCCTTTACGCTGGAATTGAAATCAGTGGAACAAACGGGGAGGTCATGCCTGGTCAG |
| ***HvGS2*** | Intron7 | **135 nt** | GTAAGCCTCCGTATTTATATGCGTGCATGTATGCTTGTTATGTGTGAATATGTGTGATGTTTGCGACCTCTTCTTTCTTTCAAAGGAAAATCGAACTGTTTAATAAGTGAAAATTCACAACCTGTATTC ATGCAG |
| | Exon8 | **75 nt** | TGGGAGTACCAGGTTGGACCCAGCGTTGGTATTGATGCAGGAGACCACATATGGGCTTCCAGATACATTCTCGAG |
| | Intron8 | **315 nt** | GTACTTCGAACAACTATCCATGGTTCCCTGATGGCCTGATACCACAGAGCTTTTGTTATTTGCTTTTAGTTAGCTTAGTATGGCTAATAAGTCAACTTTCTTTGTTACATTATTGTAAACCTGATAGGTGCATGTGCTATGAGCTTATGATTTATTCTGACACTTTGAAGAATGCCATTCTTAAATACCATAAAAACAGTTTTCGCTAAAACATCAGAACCCATAAATCCATAACTGACCAGGTGACCATCCTTTGTGATATTAATTTTCTCATTAATAAGCATTTCTAATTAACATCCTACTCACTGTAATCAG |
| | Exon9 | **54 nt** | AGAATCACGGAGCAAGCTGGTGTGGTGCTCACCCTTGACCCAAAACCAATCCAG |
| | Intron9 | **75 nt** | GTATATTTCTGTAAGTTGTTTGATGAAGCAATTTATATATTAGAAGTTCGCAGTCTGAAGATTTATCTGATGTAG |
| | Exon10 | **37 nt** | GTGACTGGAACGGAGCTGGCTGCCACACAAACTACAG |
| | Intron10 | **370 nt** | GTTCCATTCTGTTATGTTAATATTTGTTCATCGTGCGTAACTTTTATAAAGTATATCTTGTCGTTTTCTTTTGAGAAACATATATCTTGCCGCATGTATATATTGAAAAAAAGCATCAGAAAACCTATTGCAGCTGACTAGCTGTGTTAGAGTAAATACTAAGGAAAAATATTCTTCGAAATGCAATGTTTCAAACAGGGCATGGATGTTCAGTGCTGGTTATTTTAAAATTACTATAAATGTTATTCCGAATAGACATGTATTTTCTATAGTTAGTTACTAACGGTTTAAGTAGTTTTCTCATTACTCGATAGAGTATCACCTTAAATTGCAAGTGAAAAACTGTGTTTTCTTCTTGTGGTAAATGTAG |
| | Exon11 | **161 nt** | CACATTGAGCATGCGCGAGGATGGAGGTTTCGACGTGATCAAGAAGGCAATCCTGAACCTTTCACTTCGCCATGACTTGCACATAGCCGCATATGGTGAAGGAAACGAGCGGAGGTTGACAGGGCTACACGAGACAGCTAGCATATCAGACTTCTCATGGG |
| | Intron11 | **94 nt** | TATGGGTGGAGCAAACCTTTTCTTTCTTTTTTATTTGTATTTTCCTTCCTATTTATTTACAAGTTATACTGATCACAACACTTTGTTTATCAGG |
| | Exon12 | **61 nt** | GTGTGGCGAACC GTGGCTGCTCTATTCGTGTGGGGCGAGACACCGAGGCGAAGGGCAAAGG |
| | Intron12 | **206 nt** | TATGTGCTCTCCCTTGTTCTGCAACCCTACTTGCAATGGTTGGGAATGCAGAAAAAGAACTATGATGTCCCAATTAAAATTCAAATAAAATTATGACAACCCAAATAAAATTCAAATAAACCTCTTGCCTATGTATATCGGTACTACGTGTCGTAGCTTGGTAATCTGCAATTTCAACTGAAAACAATGCCTTGTTGCCCAACAGG |
| | Exon13 | **139 nt** | ATACCTGGAGGACCGTCGCCCGGCCTCCAACATGGACCCGTACACCGTGACGGCGCTGCTGGCCGAGACCACGATCCTGTGGGAGCCGACCCTCGAGGCGGAGGCCCTCGCTGCCAAGAAGCTGGCGCTGAAGGTATGA |
| | 3' downstream sequence | | AGGACCTGAAAAAAGGACGAATTCTTCTTCCGGGGAAAAGAAAATAAATCGGCGAGCGGCGAGACCGTTGGCCGTCCATTCTTGTTGATCCTGTGGTTCCGTCGGGGCACTGCCTGTACAAAATCCTCACAGTTTGTAGAACCACTCCCGCGTGTGTTTTTCCGCTTGAACTGAGTCCATTTGATCTGTTGGGTCTGTACACTCACTGTACCTGAGTCCATTTGGAGAACTACGTTATTATAAAACGATAATGAATCGCATAGAGGATCTTTTTTTCTTCTTCTTCTTTGCTTTCAGCTTGGTTCGTGTGTAAATCTCGTTGTCCCGTACGAGAGTACGAAGCTGTGGGACGCCACTATGGCCACTGGAGGACGGTGGTCCGTTTGTAGGTGGTGGCTGCCGGCTGCCGGCTGCCATTGTTGCCCGGCGGCACTGCCTCTCTTTCTCTCTCAGCTCGAGGACACACTCTGAAGAAATGGGACAAG |
| | | | |
| | 5' upstream sequence | | GACAATACTCCGTCCCCAGAGAGACACGCAAGACCGTTCGGGTTTCCGCACGGTCAAACAAGAAGGCGCCGCCCCCGGAAAATCTCCCAGCACTCCCCGGCTGAAGCGTGAGCGAGCGACCGGCGGCGGAGC |
| | Exon1 | **168 nt** | ATGGAGGCCAGGTACGCGGAGCTGCGACGCGCGGTGGAGGAGACGGCGGTGGTGGACGCGCACGCGCACAACCTCGTCGACACGGCGTCCTCCTTCCCCTTCCTCCGCTGCTTCTCCGAGGCCGACGGCGACGCGCTCGCCTTCGCTCCCCACTCCCTCTCCTTCAAG |
| | Intron1 | **1469 nt** | GTACTACTGTACTACGGTGACTAACTGACTGACCGCACGCACCCAAGCTTACACCTCACGCCGCTGCTATGCTCGTCCACGGTCCACTCCCCGGCCGGCCATCCTCGACCGCGCCGTCTTGCTTACATCCTGCTCGAGATCGCAACATTTTCTTTTTCCAAAGTAAGATAGCACTGACATTTACAACGCCATCCTTTAGTCTAGTGTGTATGTGTTTGCCCCTCCCTCTGTTGCTGCCTGCTGGGGAGTGGAGTGTGTGTGCTAGTTTGTGTCGGCCATAGAGTTAGGTGTGCTATCAGGGTCCTATACATGTTGGCGGGTTGATTTACAAATGTGTGCTCTTAAGTTTTGTCTAAGATAAATTGAGCTTTTTGAAGATTGGCCCATATCAACATAAGACCTTACGTTCTTGTGTGGTGGGTAAGATGTTGTCAATGATCATGCAGGGCTGGTATGGACATCACAGTTCCGTAAATCAGGAACTAAATTATGGTGCCATTAAAACATGAAGGAAAATAAGGAAATGTATGATTCTAGTTGTTAATGTTATATTTGCTTTCCAATCTTAGTTTTTTTTTCTTTTTTTGCAATTTTTTTCAATCTGGTTTGGTCACGCTAGATCATACACTATTCGCGTAGACGATGTGGCGTATGTAAGAAATGCACATGAATATACCATGCACGTGTTTAAAAATACATAAATATGTTAGCTATATATTAATCACATGATTAATTTAGAGAAACTATATTTTTTACATTAGAAACACATACACATAGAAACTATCATTAGACATACTTAAGTACAAACAACAAAATTATATTTAAGTGGCGTATCATATGTTTTTTCTTCTAAAACCGTGGGATGTCATAAGATTAAGAAAAAATATAATTCAAGCATAACTGGTTGGTTTTGGAGGGCACAGATGAAAATGAGTCCATATTTATTGTCTATGCTACCATGTTTGATCCGTTAGTTATTACTGACCGGGGGTATCGGCAGAATATGCGGATGAATGAGTTTGTCTTTTTCCTTTAACTGGTGGCACGGCGGAGATCGCCAGCTTTATGCCTGCTTGGCCAATCGCCATTGTATGGTGGGGCGCAGTAGGCCTTTTGCCCCGTCTCTTTGCCCTGCTGTGAGGTCCATTCCATGGATGGGCGTGTGTGCTTTTAATGGCACGCTGCTGGACCCATGGGAGGTGCTGGGGCTATTTGGCATGGCTCTTGCTTCTGAGCTTAAGATGTTCATGCCATCATGTGATGTGGGTTATTTCTTCAGTGCTCTGTCTACCTCTTATGCACCAGTAATTTTTCTGTGGTTCCAATTTCCAAATGCTTGCAATGGGAGTGCTGCTCTTTTGTCTATAAATAGGAATAATGTGCCAGTTGCTTGTTCCATAGCTATTGCATGGTATCCAAGGATAGATTTTCAGCAACTCATGACATCCTTGCAATATCATTCAATGCAG |
| ***HvGS1_4*** | Exon2 | **253 nt** | AGAAGCCTCAAGGACATCGCCGCCTTGTACGGCTGTGAAGCCTCACTTGAGAAGGTGGAAGAGTTCAGGAAGTCCCAAGGGCTGTCGTCTATCGGTTCGAAATGCTTCCAAGCTGCCAATATATCCACCATCCTCGTGGACGACGGCATAGCATTTGATAAAATGCTAGAGCTGGAAGCCCACAAGGAATTTGTTCCCACAGTCGGCAGAGTTCTGAGAATCGAATGGCTGGCGGAAACAATTATTAATGATG |
| | Intron2 | **317 nt** | TGAGCCTCTCTCTGTACTCAACGATAAAATCTTTTTTTGGGGCCACCCTTGCAATAACTAGATTCTAATAATGGTAAATGACATTTAAGAATATTTTCTGTACGAATTAGTTGCCCACATTTCTCCACAAAAAGATGTTGCCCACCGTTCATCATCTACCAGCACAATGGTTGATTTACATGACATTGTGAGCCTGAAATAACAGAATAACTAGGGTAGTTAAATATCCATGTTTGATTCATTTCTATGTTGGCGTACTCCCTTTGCAGCTAAAGATTATGATGAGCTACTGATGAAATTACTTACTGCTTTCAGG |
| | Exon3 | **68 nt** | ATTCATTCAGTGGATCAAGCTGGACGTTGGACTCATTCACTGAAACTTTTGAGGCTAAGCTCAAATCA |
| | Intron3 | **94 nt** | TATCCTCCAGATTTTTTATTTGGGACAAAGTGGAAGTTTTTTTTTCTCTTTTTGTTCTTTCAACTACATGGATATCCTTGACCAAGTTCTCACA |
| | Exon4 | **117 nt** | GTTGCCAGTAAAGTTGTTGGGTTGAAAAGCATTGCTGCATACAGAAGTGGCTTAGAGATTGATCCATGTGTTAGCAAGACAGATGCAGAGGATGGTCTTCGTCAGGAGCTAACAGGT |
| | Intron4 | **81 nt** | GAAACAGATCAAACATGTTGCCGCCTATGATTATAAGATCTATTTTATGCTTACAATCCATATTTTTCAGGTCGAAGACCT |
| | Exon5 | **95 nt** | CTTCGGATTACAAATAAAAGCCTGATTGACTATCTATTTACTTGTAGTCTTCATATTGCTGTACAGTTTCACTTGCCAATGCAGATCCACACAGG |
| | Intron5 | **94 nt** | GTAATGACTGGCATTTCAGATTTTATGAAAGCACTTTCCTGTCTGATCTTTTTAAGATCTCAAGTATATACATCTTCCTTCTACACAATTTTAG |
| | Exon6 | **158 nt** | CTTTGGAGATAAAGACCTTGACTTGCGGAAGTGCAATCCTCTACATCTCCGTGCTATTCTTGAGGATGAAAGATTCGCTAAGTGCCAACTGGTCCTTTTACATGCTTCTTATCCATATTCTAAGGAAGCATCCTATCTTGCATCTGTTTACTCTCAGG |
| | Intron6 | **75 nt** | TTGGAGGCTCGTCTTCATGCAATTATTCACCCATTCTGTTTTAAGTTAAGTTAATGATCACAAACAATCCACAAA |
| | Exon7 | **99 nt** | CAGGTCTATCTTGATTTTGGTTTGGCGATTCCAAAACTTAGTGTTCAAGGAATGGTGTCATCACTTAAAGAGCTTCTGGAGCTAGCTCCCATAAACAAG |
| | Intron7 | **756 nt** | GTACATATCCGTCGTTTGGATAATTGCTTCTATAAAATCTGAAGAAGTTATTAATAATATGAAAACATATTTTGCTTTTCCATGGTGCAGAAAAACTGAACTAATTAATATTCTCCCACAACTCAATAACGTATTATCCTCCATTGTGCCATCACATGTACATTTTTGCCATAGTCCTCCTTCCAGTGACAAAGATTGC |

| | | | |
|---|---|---|---|
| | | | TAATGTGGACACTGACGCTCAATATTACTGATGCAGTTTACTGATAAGACTTAGTAGCATTATAATATTACAAAATAATAAATTGAGATGGATATAATC<br>GTATTTTTTCATATGTTCATTTGGAGGCCCTCAATATTCATAGAGAATCAATAGTCAAAGTTTAGAAAGTTTGACTGCACATATCCCAAAATGTCACTTT<br>CTTTACTGGAAGGAGACTACTAGACACAGAGATTACTGCAGTAAAACATATGGTCCATGTTCTTGAATGGAAAAGTAGCACACATGGTCCAGGATCC<br>AGGTCCGAGCAGTATTCGGTGCTTAGTATAGATTACTGTTGGCTTTTGATATTCGATGTTTGAGAATCTCTCGCTTATTTTTGTCTTGCAATGCATCTG<br>ATTTACTATCTTGTCTGAAATAGTTACTCTTAAGTTTCCTTTCTAATAGCACTCTTGATGTATGTCTCATTGTCAGTCACATATGTTCATTCTCATCTTCT<br>GTTGCTTCTGGAGATACTATGTGCATGATGTTTTTATTTGTTAACTGTTAACCATTGGCAG |
| | Exon8 | **51 nt** | GTCATGTTTAGTTCAGATGGATACGCTTTTCCGGAGACATACTATCTAGGT |
| | Intron8 | **593 nt** | ACAATAATCAGTTTTAACTCTTTCATAAGATGATGAAAGATCATTGTAAGTCAGTACAAATGTTAGTTATATTATCATAAATTAAGTAGGTCAAATAGTA<br>TCCTCTCATGGTAGGAAAACAAACTTTTGGGCCAATAGAAAATACAAAAAACAGAAAGATTTGGCACAGGATCTAGACCTTCAGATATACCACCTCTG<br>TTCCAAAATACTACTCCCTTCGTCCCATAATATAAGACGCTTTTTAACACTACACTAGTGTCGAAAAACGTCTTACATTATGGGACGGGGGGAGTACA<br>TGATTTCCTAAAAAGTGTACACTCAACCTTTTCTATCTTTGACAACTAATTAACAAAAGGTTGTTCAGATTTGTTTGGTAACAACTGTGACGTTAGATTT<br>GTCATGAAAAAATAATTTCAAAATATTTTTTACTAACATAAGTATATGAAAAATAGCGTCAAAGTTACGTATTTTGTAGTGGAGGGAGTATATAAATAAG<br>TAGTGCAGACAAGGAAAGTTATCCAAAAACCACACAAGAGTGTCCATTTAGTTGTTGATTTTAAGTGATAATACATGGTTTGTACTGATTTGCAGGT |
| | Exon9 | **266 nt** | TCAAGAAGGGCACGTGATGTTGTTTACCATGTCCTATCAGCTGCATGTGAAGATGGTGATCTTAGCATTCAGGAAGCTATTGATGCAGTTGAGGACA<br>TCTTTAGAAGAAATGCATCGGATCTATACAAGTTGAACGTTGCCAATGGGTCAACTCACCAGAAAACTATGATAGCTGACAGTAGGATAGCATCATCT<br>TGTGTTGAACAAGATGTCCTCTTTGTTCGCA TCGTCTGGAATGATGCTTCAGGTCAACATAGATGCCGCGT |
| | Intron9 | **339 nt** | AAGCACATCTTTCCAAGCACATGCTGCATTGTTTACATTGTGTGATGTAGTTTTCTCACTGATCATTTCTCTTCTTTTTTATTGACAAAAAATTCTCTTTT<br>AGAAAATTGTCTTAAATAAATTACCAACTAGACACAATAGTTAACATGAACTTTGTTTGAAGATTACCTACAAGTTGTTTCGTTTCAATAGATGGTCAAC<br>TTAGCACGTATAAAACTACTGATAATTGCATGGTGTTGACTGTGTTGACGGTCAGCTTACCCACCTGAACAACGGCATTGTTTTTAGAATTGCTAATG<br>CCAAATCCAATTTTGAGAAAATAGAACTTCACTTTCAGGT |
| | Exon10 | **173 nt** | TGTCCCAGCCGGAAGGTTTTATGAGATTGCAAGGAATAAGGGTGTCGGCCTGACTTTTGCATCAATGGGAATGACTTCCTTTTCTGATGGCCCAGCT<br>GATGGGACAAACCTTACTGGTGTAGGAGAGATCAGGCTTATGCCAGATATGTCAACGCTTTTGAGACTACCATGGT |
| | Intron10 | **168 nt** | AAAGTTAATTCATTTAACGTTTTCTCTGTACTTTGTATCGAACCACCCCTTAGAATCCCATAACTTAGGATCGTATATGAATAGTTTCTTGTTTGTTAAC<br>AGCAGGTCAATTCTCTGAACTTATTAAGGCATCATTGAACTAATGATAGTTTACTCACATTGCAAGGT |
| | Exon11 | **119 nt** | CAACACGTGAGGAAATGGTGATAGCTGACATGCAAATTAGGCCTGGAGAAGCCTGGGAATACTGTCCTAGATATGCCTTAAGAAAAGTCACAAAAGT<br>TCTGCTGGATGAATTCAATGTG |
| | Intron11 | **115 nt** | GTAAGAATAATCCCAGAATGTTGCCTACATGACTCTGAGAAGATCATTTTCGTCTCATGAGTTCTTTCTGTGCACTTGTTTTTTGTATTTATGCTTAGTT<br>GTTTGTTCTTTGTAG |
| | Exon12 | **53 nt** | ACAATGAAGGCAGGTTTCGAGAATGAATTTTATCTGCGGAGAAAATTAGTAAG |
| | Intron12 | **83 nt** | GTAGCTTTTTCCCGACTCTCCAAAATAAATTATGATAAAGGCCATGTTGTTCTGTATGTGACCCAAATCAGAAAACTCTACAG |
| | Exon13 | **130 nt** | TGAGGGGCATGAGCGTTGGGTTCCATATGATAATAGCAGTTACTGCTCAACCTCATCATTTGATGGTGCCTCATCTATACTACAAGAAGTGTATTCTT<br>CTCTTAAAACGGCAAATATTGTGGTTGAGCAG |
| | Intron13 | **147 nt** | TAAGACTTTATTTTACCACCAAAAAGATGTCAAAACTCGTGATGTTATATATCAAACTGTTTACCTTTTTGATTGTATGTCATGATTTAGTGATTGATCA<br>TTTTTTCTATGTTTACATTCACTTTCTTGCTCAACAAAATATAAAAG |
| | Exon14 | **146 nt** | CTGCATGCCGAAGCTGGGAAAGGGCAGTTCGAGGTTGCCTTAAAGTATGTGATGTGCACTCTTGCTGCTGACAATTTGATATATGCTCGCGAGATTA<br>TTAAATCTGTTGCTCGAAAGCACGGGTTGATAGCAACATTTCTTCCAAA |
| | Intron14 | **350 nt** | GTAAGCCAATGGATCTACAGTCTGTTTTTTCATTAGCATGCTCTATAGTTTGAATTGTATTGTGTACACTGCTTTGTGTTAACAGCAAATCAGAACACC<br>AGAGAGTGTGTCCAGGCAACATTACTGGCTTGTAAAAGAATTGACTTTTTACTTAGAAGCTTAAGAGAACTGAAATGAGCTGCGGTTGATCAAATAAT<br>ACTTCTATTTGCTGATATCCCTATGTTATACTCTGATGCATTATGCAAATATCAGGTTTTAGCAATACAAATGTGTGATTTGAAAACATAAAATATTGCC<br>AATAAGTGAAACTTGCCTTTCTGGTGCCTGATGCTTTATTCTGCTGGACACAG |
| | Exon15 | **189 nt** | ACCTGACCTGAATGATATTGGATCGGGTTCCCATGTGCATCTGAGTTTATGGAAGAATGAGCAGAATGTTTTTATGGGATCAAATGAATATAGCCACT<br>ATGGAATGTCAAAAGTTGGAGAACGGTTCCTTGCTGGAGTATACCGTCACCTTCCATCAATCTTGGCATTTACTGCTCCTCACCCTAACAG |
| | Intron15 | **217 nt** | GTAACATCTTCCTGAAAAGCTTCGAAGAATACATGTTAATTCGATAATAGCTTTGATAACTGATTTTGGTATTTGTTTTTTTGCATAAACTCTCTGATGT<br>CAGCGTATTTTTTATTATCTAGAATCTGGATAACACAGTTTTCTACTTGTTGGAAAAATGCCAGAACATCAAAGTAACCATTTTAACAATCATTATTATT<br>GGTTTCTCTATATGTAG |
| | Exon16 | **233 nt** | CTATGATCGAATTCAACCAAATACATGGAGTGGAGCTTACCTATGCTGGGGAAAAGAAAACCGGGAGGCTCCATTGAGAACCGCATGCCCACCTGG<br>TGTGCCTCTCGACATGGTCAGCAACTTCGAAATTAAATCATTTGATGGGTGCGCAAATCCGCACTTGGGGCTTGCTGCTATTGTCGCTGCTGGGATT<br>GATGGACTTAGGAACGGCCTTGAGTTGCCTGAACCAATTG |
| | Intron16 | **697 nt** | GTAGGAAAAAAAACCCGTTGCTTGACCTTTTTTATATTACTCTCATGCATTACTGATACTCCCTCCGTTCCTAAATATAAGACCTTGTAGAGATACTAC<br>ATACGGAGCAAAATGAATNNNNNNNNNNNNNNNNNNNNNNNNNNNNNNNNNNNNNNNNNAGTTCATAGTGAAATCTCTAAAAGGAGTATTTGTCATG<br>CTGCCATGGTGTCTGAAAATGCCCTAGGAACATATTGAGCATGTTCGGTGATCCTTGCAGGAACATGTACAACATGATAGAAAGTTGTCGAAAAACA<br>CAACTGAAGGTCCTGAAAATTCTGGGGAAAACTATCATGACATGGACACAATGCACATCTGTTATGCCCACATAGTGTCAAATCCAAATGAAATTCAT<br>AGCTTGATAAAAGGAAAGAGCTCAGGCTGGTACTATTCTGTCGGATTTGTCTTCTTTTTTTGTTGATCTGTGGGATGAAATTTGGAGACATGGTAGAT<br>GTTGTGTAATGTTGTGAAAGAAAGTCAGATTTCCTCATTATCGTTAAAGTTGCTAATCAATAATCTAACACATTACTATAACTTATGTGCCTAATGGATC<br>TGGCATGCCCCTAAACATGTTATGCTATATGTCTGGATTAGATTATTTCTGCCGACTAGCTTTTGGTTTCCTCAGTGCAAATGTTACATGTGTCTTGTG<br>TCTCTTCCAG |
| | Exon17 | **144 nt** | AATCAAATCCTGCAGATTATGCTACCAAGCTCAAAAGGCTACCGCAGGACTTGCTGGAATCTGTAGAATCACTTGCTGCAGACAAAACTTTGCATGA<br>GCTAATCGGGAATAAGCTTATTACAGCTGTTATCGCTGTACGCAAGG |
| | Intron17 | **144 nt** | TTAGTACTCATAAGTTGCTCCCTCCCAAAGTGTCTTGGGAGGGGGTATTATTTTCCTTCACGTGATATAACTCGACAGATTTGTCAGAAATGAAATGA<br>AAGCATGAGCGCGACTAACATCTTAGGTTCTATTTTATTTTTCAGG |
| | Exon18 | **59 nt** | CGGAGATTGATCATTACTCGAAGAACCCTGGGGCATTTGGCGATCTCATTCACCGTTAC |
| | 3' downstream sequence | | TAAGACAGAATACTTGTTTCAAGACCACTTTCTCTGTCGCTTGGAAGGTTAAGCCGACGAATAATGTTGCCCACGAGTTACTGATCCTCATGACTGAA<br>TTTGCGTGTTTTTCAGTTAATAATAAAGTGCTTAGAATATGAAATCTGTCATCGGACATTCTTTTTTATCTGACGAAAAATCGAACAGAAAAGATAAGA<br>CTTGATGCCTTCGTCTCTTGCTTGAACCCCCAAAAAAAAAAAAAAA |
| | | | |
| ***HvGS1_5 Fragment*** | 5' upstream sequence | | AGGAGTCACTTTCTGGATCAAGCTGGACACTAGACTCATTCACTGAAGCTTTTGTGGCAAAGCTCAAATCATATCCTACACAATTTCGGATGTTGTCT<br>GTTTGTGACTATGTGTGTGCGCGCAAGTGCATGTGTGTCTCTTTGTTTGTTTGTTTGAACTCTGTAAATAACTAAATATCCTTAAAAAATTCCTCACAG<br>TTACCAACAAAATTGTTGGATTGAAAAGCATAGCTGCACACAGAAGTGGATTGGATATTGATCCAAGTGTTAGCAAGGTGGATGCAGAGGATGGTCT<br>TCAGAAGGACCTAGCAAGTAAGCATATTTCTTTGTTGGGAGATAGAAATGTGTAAGCCGATAAAATAGGGTTGCTAGTTAATAATGACAATGGCCCAT<br>TAAGCTTATTAGTCGTCAATACTTTTCAGCACGGAGGCCTCTTCGGATTACAAACAAAAACCTGGTTGACTATATATTTATTCGTAGTCTTGAAATTGC<br>TGTGGAGTTTCACTTGCCAATGCAAATTCATACAGGGTAAGGACAACATTTTAAATCCTATATATCAAAACACGGTGGATTAATTTCAATATGTATCTC<br>TAGTATAAATATGGTTGTGTGCATCAGACAATCAGAGGCCAAGGATCATCCCCTCTTTTTAAAAAAAAAATCTCCAATGTATATGGCATATGATTTCCA<br>AGCAATTTCAGGTTTGGAGACAGGGACGTGGAATTACGAAATTGCACCCCTTTTCATCTGCGTTCTCTTCTTGAAGACAAAAGATTTGCCAAGTGCC<br>AAATAGTTCTACTAAATGCTTCATATCCATTTTCAAGGGAAGCATCCTATCTTGCATCTGTTTACTCCCAGGTGGAACCCCATTTTTACTACTCCCTCC<br>GATCCATATTAATCATCGCAGCTTTAGTAGAAAGTTGTACTAAAGCTGCGATAATAAATATGGATCGAAGTGAGTAACAAATTACACATTGTCCGTTTT<br>AATTATTTTCTTCATTTTAAGTTATTAACCATCTTACACAAACCACAATAGGTCTACCTTGATTTTGGCTTTGCATTTCCAAAACTTAGTGTTCAAGGAA<br>TAACATCATCACTTAAAGAGCTCCTAGAGCGAGCTCCCATAAAGAAGGTAAAGTATCTGTCTTCTGAATAGCTGTTTCTAGGAAGGTCATTGTTACGA<br>ATACATATTTTACTTTACTTTTCATGGATAGTGCTGAATACAAACTGAGGATTCTCATGCTGTTGGAACCAAAAACAACTCAAAGGTAGTACTCCTTCT<br>AGTCACCACTTGTCTTAGTGGATACCGACATAGTGCCAAAACTCCTTTGACCCTGTGAAATATTTATAAATACAGTACGGTTACAATATTAAGAAAATA<br>TTTGTGAAGGCAAATCACCTCACACTTTATCCAGATGCTCAATTTTAAGTTTATAATATTCACACAGATATTTATAGATAAATTAATTAATTAAAATTGAC<br>TGCATCTATATCAAGACCCTTGTCAATCCTTGTCTAGTATTCACTCTCATGATTCCTCTGAACAGAAGTCCATTGCTATCAATTCTGCTCATATTTGAT<br>GCATGTATTATTTTTATCTGAAGTTTGCCACCTCTGGAAATACTATACTCTCATACACCTAGCAATTTTTAACATTAATTTCAGGTCATGTTCAGCACAG<br>ATGGATATGCTTTTCCCGAGACATACTATCTAGGTATGTTTATCCATCTTATCTATATGAAGTACTATTAAATAAGGTGAATCTTATAAGTCAATACGAA<br>TGTATATGCTTATAAAGAATAAGGGACTTTGAACTGAATAAGTTAGGGAGCAATAATTTTTAAGTTTGTCACAAATAAAAGTTAAAAAAGTGGAATATA<br>TATTTGATGTGATACAGAGCCTATTGATATACATATTTCCTAGTTCATTTAGGAGTTTAGGACCGTAGACGTACAGTTATTTCTTTTAGGTATTGAGTTT<br>TAGCTTGGGCTGATTTACAGGTGCAAAATGGGCACGTGATATCGTTTACCGTGTCCTCTTAGCTGCATGTGAAGATGGTGATCTTACCATTCAGGAA<br>GCTATCGAAGCAGTTGAGGATATCTTGAGAAGAAATGCACTGCATCTTTACAAGTTGGATGTCGTCCATGAAAAAGTTATTAATATTGATGATAATAG<br>CACATCATCGTCATCCTGTGTCAAGAAAGATGATGTGATTCTTGTTCGAATGGTTTGGAATGATGCTTCAGGTCAACATAGATGCCGTGTAAGCATAT<br>CTTCCAGACCACATACAACATTGTTAAGTTTTTTTTTCCTTAGAATTGTTACTATTTTAGTAGGTAGTCTTGAGAAATGGAAACTTTACTTTCAGGCCTT<br>GCCAGCCGAGAGATTTTACGGCATTGCACGGAATAAGGGTGTTGGTCTGGGTATTGCAGCAGTGGGGTTTACTTCCTTTAGAGATGCCCCAGCAGC<br>TGGGACAAATCTTACTTGTGCAGGAGAGGAAATCAGGCTTGTGGCAGATATGTCAACACTTTTGAGGATCCCATGGTGAATGTCCTTAATTAATTTAT<br>TTTTGAACAAATTATTGTTATTTTTTATATATAAATTGGGTCAAACCTTAATTTTTGAACAATTTACTTATTTCTTATTATCGTTTCTCTCTCACACAGACC<br>CAACTTTCCTTTTAGATCAACCACCCCAACTTGTACATAAGCATGCCGATTTGTTGCTTATACCTGGCGAAGGATATCCTTATGGTTTCTGTTCTGTAC<br>ATAACTACTAGTCCCTCTGTATCACAATATAAGAGGGTTTTTTATAATCCCTTTGTATCAAAAAAAAGTATATTTCGATATAGAGGGAGTAATTACTTGG<br>TTCTTATCAGGCATATGCATTTCCAGATTGTTCACAACCTATCCGGACAAACCGAGAATTCACTACGACATAGTCTTAACAATGGCAACCTTAAATTTT<br>TGAAGGAAAAACATTAGCTTGTGATTGTACTAATCGCACTTTACACTTCTTACAAGGTCAAGAAACGAAGAAATGGTGATGGCTGACATGATAACCGG<br>TTCCGGCGAAGCCTCCGAATACTGTCCTAGAAATGCTTTAAGAAAAGTGATAAAAGTTCTGCTAGATGAATTCAATGTGGTAAGAATAAACCTATAAA<br>AATGTAGTTGATGTCTTTTTGTTGATGCTTAGTTTTTTTTCCTAGACAGTG |
| | Exon1 | **45 nt** | ATGGCAGGTTTCGAGAATGAATTTTATCTCCTCAGGAAATCATTC |
| | Intron1 | **93 nt** | AGGTAGCTTTGCCTGACTCTGTAGTCTGTGTAAAATTAATCGTGGTAGGAAGGATCTATCCGCTCGTTCTTTATATGTACTGAAAACTCATGC |
| | Exon2 | **129 nt** | AGTGAGGGCCATGAACAGTGGGTACCATATGATAATAGCAGTTACTGCTCGACTTCAGCATTTGATGGTGCATCATCTATACTGAAAGAAGCATATT<br>CCTGTCTTAAAGCTGCAGAAATTGTGGTTGAG |
| | Intron2 | **126 nt** | CAGGTAACAAGTGACTTTTGTAGATGTGAAGAACTTCTGGTGTCAAATTGGTTACCCTTTTTTTGTGTATTGATTTAGTGATCTTTTTTTCTGTTTTCAC<br>CTATATTCTTCCTCCTGCAAGTATAA |
| | Exon3 | **149 nt** | CAGATGCATGCTGAAGGTGGTAATGGGCAGTTCGAGATAGCCTTAAAGTATGTGTTATGCACTCTTGCTGCTGATAATCAAATATATGCCCGAGAAA<br>TTATTAAATCCGTTGCTCGAAAACACGGAGTGATAGCAACATTTCTACCAAA |
| | Intron3 | **95 nt** | GTAAGTCAACAGATTAACATATCGTAATCCATCTTGTTCAATGACACAAGCAGGTATCTAGTATATGATGCAATGTTTTCATTGGCAATTTCCAG |
| | Exon4 | **186 nt** | ACCAGACCTGAATGAACTTGGATCCGGTTGTCATGTGCACCTGAGCTTATGGGAGAATGACCAAAATGTTTTTATGGGCTCAAATAAATATAGTTACC<br>ATGGGATGTCAAATACCGGTGAACGGTTCCTTGCTGGAGTTTATCATCATCTTCCATCGATACTGGCATTTACTGCCCCTCACCCACA |
| | Intron4 | **260 nt** | CAGGTATTACTTCAATCATAAGCAGGAGATTTTGTAAATATAGTCTCCAATGCGATTGAATACCCAATCACTACTTTTTTAGGCATATGTTGCTAGACA<br>TTTATGGTTTTTGCTATCCTGAAAGTAGTTTTCATGGCAAGGTTATTGATTTTTTTAGGATTACCAATCCAAATATTTTTTGTAAATACATATAGTTTTTT<br>ATTTGTTTACCGAAGATGAGGGGTATGATGATTGTGATTTTATTTCTGTGCATACATATA |
| | Exon5 | **236 nt** | CAGCTATGACCGAATTCAGCCACACACATTTACTGGAGCATATCTTTGCTGGGGGAAAGAGAACCGGGAGGCTCCGTTGAGGACCTCCTGCCCACC<br>GGGCGTGCCTCCTGATTTTGTCAGCAACTTCGAGGTCAGATCTTTCGACGGGTGCGCGAACCCTCACCTTGGCCTCGCCGCCATCGTCACCGCCG<br>GCATCGACGGATTAAGGAGAGGCCTGAAGCTGCCTCCACCAACTG |
| | Intron5 | **91 nt** | GTAAACACTAATTTCTCATTGCTTTCTTGAGCTTTCAACATCTTAACCACGAGTGTTTCACTCGGGGAAATGTGTCTCTTGGCTCTTGCAG |
| | Exon6 | **141 nt** | AACTGAATCCTGCAGATTGCGCTTCACACGAGAGGCTACCGCACGACTTGCTGGGATCTGCAGAGGCACTTGCGGCGGACGAGATTTTTCATGAAC<br>TGATGGGTGATAAACTTGTTACTTCTGTTATAGCTATGCGAAAGG |
| | Intron6 | **71 nt** | TAACTAGTAGACACACTCCAGTGTACTGTTTCCATCTGCTCTTCATATTACTAATCTTCGTGATCTACAGG |

| | | | |
|---|---|---|---|
| | Exon7 | **62 nt** | CTGAGATCGAGCATTACGCGAAGAACCCAGTGGCAGTCCACCATCTCATTCACCGTTACTAG |
| | 3' downstream sequence | | CGATGCCTATTTGCCTTGCTTGGTGATCGATCGGGATCGGGTTGAAAAAGTACTGCTCTGCTTTCAGACGTTGCTCTGTTCTCAGCACTTCACATGT<br>ATGTGGATGAGTAAATGTTGTCCAGATGATTGCTCATGATCTCGGCAATGCACTTGCCGTTGTCAGACTTTTTCAGTTAATAAGTGGTAGAGACCAAG<br>GTTTAGATGATTGCTCACCGACAAAGCTGCGCCAGGCCCTGGGATGGTGGCAGCAGGGCCATTTTCACCCTCCGCCCTTCCCCCAGATCTGGTCC<br>CCGGCAGCACCGCCAGCTTCCACCGACGACGGGGGCGGCCTCCCCGGCCATCCCGTCCTCGAGGTGCTGCTCCCGTCGGTTGCATGTTCAAAAG<br>TTTGATTTGTCAGTACCGGAGCTTGATGTGTCGATGTGTTGGATCTTCAGGCTATGAGCAACCCTTACACCTGATAAAACTGATATGTGGATGTGGAT<br>TCACACATGTGAGTGGCTTGATGTATACAATGGACATATCTATCTTTCTTTGACAAATCTGTGCATATATAAATGTCGTACAATTGATGC |
| | | | |
| ***HvASN1*** | 5' upstream sequence | | GATATGCTCAGACTGCTATGCTTTCCTCTGCTTCGCTGGATATCGTCGTCGTCGATCGTTGTCTACAGCCAACAGCGAGGTACGTAGTCGATCTGAA<br>TTGTGCATCTAGTATATACGAGTATTACAGATGCACAGCGTTGAGACTTCCTTTGCAGGAAGCCTATTATAATCCGTGTCTGGACACTGAAAAATCAG<br>AGAAAGAGAAAAGAAGAAAAGAAAGAAAAAGGAAGAAGGTGTTGGGAGCTCGATACTTTGAATTCTCATTACAAAGAATCGATCATCCGTGACGACG<br>GCTGGGGAAAATTTCGAGAGGAGTCACTTGACAGGGCAATGCCGCTGTGGGGGGCGTTCACTTCCATTGGTTGGCGACGATGTCGGCGAGGTCG<br>ACGACC |
| | Exon1 (no match in genome seq) | 314 | AGATGGGCGATGACCTCGCAGTCACTGCCGGTCCGGAACCTGTGGCCGGAGAGCCGTTCCCGGAGCTCCTCATGGTTGTAGATCTCCCCGTTGAC<br>GGCAACAGCAACGGACTTGTCCTCGTTGTAGAGCGGCTGGTCGCCGGAGGCCGGGTCGATGATGGCGAGGCGCTGGTGGCAGAGGTAGTTGTCG<br>GCGACCTGGTGCAGGCCGCTCCAGTCCGGGCCGCGGTGCTTGAGCCTGCGCGAGAGCTCGAGCACATGCACCCTCTTCCCCTGCGACTCGTCGC<br>CGCACCCCAGCACCGCCAGTATGCCGCACAT |
| | Exon1 | 144 nt | CCATCGATTCCCCAGCCAATGTAGAGAGACGTGACACCAATGGCATCACGAGCAGCAATGAAGCTGTGATCTCGTGCGTCAAGCAACACGAACGA<br>GAAAACACCATCCAACATGTCAATGAAGCTTTCTCCATGTTCCTCATAC |
| | Intron1 | 100 nt | CCTGACAATTTTTCAGAGACGTGCATGTTTAGAAACGCAGTGCAAATGATATAGGTTTGTTTCATGAAAAGATTCCTCAAGCACTTTTGAGAGTGTTA<br>TA |
| | Exon2 | 188 nt | TTTCAAATGCCGATCTGAGACGTAGTGGGTCATAGGGAACCGAGGGGATGACCTCAGAGAACCAAGGAGGGTTATACCATCTCTTGAAGCATTTTT<br>CTTTGCTGGAGTAAAGATTACCAGGTGGGAAGATCTCGAAGTGCTCACAATCATCGTTTAGTCCTTTCATCTCCGAAGATATCCACACTGAA |
| | Intron2 | 108 nt | GAATGAAGAACATGAAGAATCATGGCCATGAGTCCGAAATTTTATTACATCTCTGTTTTTCAAAGATACTAGATGATGGTTAGTTGAGGCCGTTACAG<br>TTTGCGACCT |
| | Exon3 | 162 nt | CAAGCCCAACACAGAAGGAGTGGAGCCTAGTTCCCCAGCGCTTTGCAGCCTTCGTCCCTGCGAAATGACGGGCTGCGACGGCTGCCACCAGTGAT<br>GAGTCGAGGCCACCAGAGAGGAGGACACCGAATGGAACATCTGTCATGAGCCTCTTGATGACAGCCT |
| | Intron3 | 90 nt | ACAATATAAATTGAGATAACAGCATGGATTCAAGTTCTATTACTCCGAAACTTCAATAAACGTGTACATGTTCCTTTCAATTTCACACCT |
| | Exon4 | 71 nt | GAACTGTGAAGTTGAACTCATTACTCCGAAACTTCAATAAACGTGTACATGTTCCTTTCAATTTCACACCT |
| | Intron4 | 85 nt | ACAGCAATTTGTGCAACTAAAATAAGTGATTGTGTACAAATAAGATAGGATCGTTATACTTCTTAGTGGTACTACTACCGTACCT |
| | Exon5 | 222 nt | TCCGACATGTTTCTTGGTGGAACTCCTCCTTGTTGGGGGCCTTGTGGAAATACAAGTACCCCCCGAAGATTTCATCAGCACCCTCACCAGAGATGAC<br>CATCTTGACACCAAGCGCCTTGATCTTGCGTGACATCTGGAACATTAGTGTGCTTGCCCTGATCGTCGTCACATCATATGTTTCAATGTGATATATCA<br>CGTCTTCAATTGCATCGATGCCGTCCT |
| | Intron5 | 84 nt | GCAAAACAAATCATGTTAGAGCAGAGTGTTATGTGTCCTATATATTGTTTCTGCTCCTGTGAAATGTTTGTATTTTAAAGACCT |
| | Exon6 | 136 nt | ATCTTCCATTCAGGATCTATGCTCATAGCCTCATCGATGAACTCCTTGTCCAAGAATGGCACACGAACCTCAAGGCCCCATGCAGATGTTGCTTTATT<br>GGCCCTCAAGCAATCGTACTGATGGAGAGCTTTGATCT |
| | Intron6 | 65 nt | TGTGTGATTAAATTTAGAAAACAATGCAGTGCACTCCCATTCTTGTGAAATATTTTGTTCTTACC |
| | Exon7 | 69 nt | GGTTTCTCCTCATCGTCGAACGCTTTCCTCAGTATCCATTTTTCAATTCTTCCAAGATCAGGCCGGATC |
| | Intron7 | 89 nt | CATAGATCACACAGAAGAACGTGTTAATAACTCCACTACTCCAGCTGGATCTGACTGTTGAGTCGAAAAATGTTACCTTCGGCAAGAAG |
| | Exon8 | 100 nt | ATTTGATGCTGCATGATCCTTAAGGCCATCAATCCAGCTATACCCAACACCATCACTAAACTGCTCCTTCTGCCTGTACAGAATGTGCTTCGGCAAGA<br>AC |
| | Intron8 | 118 nt | CTGCAGTCAGTGATTTCATTTCATATCAGTTAGATATTGCAATCACATACTCGTATCGAGGAACGTACGTATTAGTTTTACCAAAACTAATTAACATGG<br>AAAATATTTGTTTATTTAC |
| | Exon9 | 104 nt | GGGAAGTACCTCTCGAAGATCATCCTGTAATAGTAGGCCTCTTTAGTTGTTGGGGTGTTGTGTGGGTAGATGAACTTTGCATTGGACATCATCTTAT<br>CACTCAC |
| | Intron9 | 141 nt | AACAAGGTCAAGCAACCAACATTGCTTCCATTAAACATTGCTCCAATGGTATTGAAGTTAAGGTATCTATTTCATTCCTTTACGCAAAAGGATAATGTC<br>CATGTTGCTCAACATTTCTTTTGTATTTACTCACTCACCTGG |
| | Exon10 | 223 nt | GCTCTCTATGGCAACACCATTCACCACGGTCTCCATCGGCCCAGGCTTATTGCTACCACCCACAGCGATCGTGTCCTGCTCGTAGGCTGAGAGATG<br>GACTCCAAGCGCTGCTCTCCCAGAGGGGTCCAGGTTCCCAGACCACTGCGCGTCCCACTCTATTGCCTTGGCTGTGCTGCACGCCACGCTTGGCC<br>CGCCTGGCACCGTGAGGATCGCCGAGCTCTGA |
| | 3' downstream sequence | | GAGTATCATATCATGATACCGTATCATATTAAATGTTGTGCTAGTATGTGTTATGCATGGCAATAAATGATATATCATATGATACTATGCATTACGGGT<br>GTAGTATCATATGCATGATATTAGTATATGATACTCTCCATTACAACCAGCCTAACCGGATACACCATAAATTTCACCATCATATTTAGCTTATATATGC<br>GGGCTATAAAAAGGACAAGTAACGACCAAACAGACCGGACCCACCATAAATTTCAGCAAATCCCGCTCCCCTAAGCTTGCCGGACAAGCCCCACTT<br>TTCTTCTCCACTCACCTCCCTCGTCCACGCCGGCTCTCACCTAGCTTTGCTTGCCATCCTAGCTCACCGACCCCTCCAATCTAGATTTTCCAATTCAT<br>AGGCCAGGCCAAGAGAATCTTTTAAATTAAACAGGAGAAACATAGGCCTGTCTTATTACAACACCGACAAGAACTTATGCATCATATATGTCTTTAGT<br>AGGACACGATATACGTGTCATTCATCA |
| | | | |
| ***HvASN2*** | 5' upstream sequence | | TTTCGCTGGATCGACGGCTACAGTATGTAGCTAGAAGACCTCTCTCCGTTCTTCCGAATGAGGATGGTTGTC |
| | Exon1 | 219 nt | AGTGACGACGATGGACTTGTCCTCGTTGTAAAGTGGCTGGTCGCCTGAGGCAGGGTCGATGATGGCGAGGCGCTGGTGGAGAGGTAGCAGTCA<br>CCGACCTGGTGCATGCCACTCCAGTCGGGGCCGCGGTGCTTGAGCCTGCGCGAGAGCTCGAGCACGCGCACTCTCTTCCCCTGGGTGTCATCGG<br>CGCAGCCCAGCACTGCCAGTATGCCACACAT |
| | Intron1 | 79 nt | CTGCAAGGAGAAGCTTGTTTTAGCAGAAAGAAGCAAGCTACAATAGTGGCAGAGAAATTCACACAGAAGGTAAACATAC |
| | Exon2 | 96 nt | CAGGTGTGCGATGACCTCGCAGTCGCTGCCTGTCCTGAATGTGTGGGAGGAGAGCTGCGCCCGGAGCTGTTCATGGTTGTAGATCTCTCCATTCA<br>C |
| | Intron2 | 324 nt | CTATACAATGGTAAAATTAAGTTATGCATCCACTTGTTAATCACATATATTTCCAGTTTGGTTAATTGTCTTTTTATCTTCATGTTTATCGATGACATGG<br>AATCATTTCTCTGTTTACGGTGGTCAAGGACTACAAATGCAGGAAATGTCATTCTGGTGTTATTCGTGCCTTTCCCGTACCAGAGGTGTTCTTTTTTT<br>GACTTGTGATAGATATGTTAGTGTCTCGCAACTTGGCATATCTTAATGATATCATGACTAGTAATTATTGTTAAGGAGGAGGAAGAGGAGAGTTGACA<br>TTTGAGTGTTGTCATGTAGGTGACTTAC |
| | Exon3 | 141 nt | ATCAATTCCCCAGCCAACATACAGGGGTGTGACACCAATGGCATCACGGGCAGCAATGAAGCTGTTGTCGCGTGTGTCGAGCAAGACGAAGGAGA<br>AGACACCATCCAGCATGTCGATGAAATTCTCCCCATGCTCCTCGTA |
| | Intron3 | 138 nt | TGGCAATTTGCAGAACATGAGCAGGTATGTTAGACAGATTTTACTTGTCCTTGACTAGGTAGTATTACTACTGTGTGACAAGATTGTCTGAGGGTAGG<br>TATTGTGTGTCATTATTGAGTAAAATTAACATGACTCACC |
| | Exon4 | 192 nt | CTTTTCGAAAGCTTTCCTGAGAGCAAGTGGATCATATGGCACTGAAGGAATGACCTCCGAGAACCAAGGTGGGTTGTACCATCTCTTGAAGCCTCC<br>CTCCTTGCTGGAGTAGAGATGACCAGGTGGAAAGATCTCAAAGTGCTCACAATCATCATTCAGGCCCTTCATCTCTGATGATATCCACACCGACCC |
| | Intron4 | 83 nt | CTGAAAGAAGACTACACAGTTAGACCATGGTGGGATCTTAAAATTGTTTTTTTTTTTAATTTCAGGCAATGGTCTCGTCTGAC |
| | Exon5 | 160 nt | CAAGTCCGACACAAAAAGAGTGAAGCTTAGTCCCCCAGCGCTTTGCAGCCTTTGTCCCTGCCAGGTGACGAACTGTAACCGCTGCAACCAATGATG<br>AGTCAAGGCCACCAGAGAGTAGAACACCGAATGGAACGTCTGTCATAAGCCTCTTGGTGACAGC |
| | Intron5 | 80 nt | GCGTAATAGTAATGTGGTGCAATTTGTTGGAAATCTGCTAAAGGTTACAGTCATTGATGATCTTTTTCAGTTCCATACCT |
| | Exon6 | 82 nt | TGAACAGTGAAGGTGAACTCATGGTGCATGGTGCCCAGGTAATTGGCTACCTCCTTTGCAGCCTTCAGATCAGGTGACCCCT |
| | Intron6 | 81 nt | TGCAATGCTTTGTGCTATTAGTACAACAACTAGTCTGGTATGATCGACAGATGAAGCATGCAGCTTCTCATGCTTGCTACC |
| | Exon7 | 221 nt | TTTGACATGTCTCACGGTGGAGCTCCTCTTTGTTGGGTGCCTTGTGGAAGTAGAGGTACCCTCCAAAAATCTCATCGGAACCCTCACCAGAGATGAC<br>CATCTTGACCCCAAGTGACTTGATCTTTCGTGACATCAGGAACATTGGTGTGCTTGCCCTGATTGTTGTCACATCATATGTTTCGGTGTGATAAATCA<br>CATCCTCAATTGCATCAATGCCATCC |
| | Intron7 | 68 nt | ACAGTGTAATCATTATGTCAGGGTGTCTGAATCCGCCATTTTTCTGAACATTTCTATCTTATTCACCT |
| | Exon8 | 136 nt | ATCTTCCACTCAGGATCAATGCTCATTGCCTCATTGATAAACTCCTTGTCCAAGAATGGCACACGTGCTTCGAGGCCCCATGCAGATGTTGCCTTGT<br>TGGCCCTCAAGCAATCGTACTGATGCAGAGCTTTGATCT |
| | Intron8 | 115 nt | TGTAACATCAGATATAGTAATTGTCTAAAAGTAGAAACTGGGAAGACATTTTACAACAATATGGTATGTTCTTTCTCAAGTTTCTAGATTCGTAAGGTT<br>TGAGCTTCAGCATACC |
| | Exon9 | 80 nt | TCGGCAGGAATGGTTGCTCCTCGTCATCAAATGCTTTCCTCAGAACCCACTTCTCAATTCTTCCAAGATCAGGCCGGATC |
| | Intron9 | 86 nt | GCAATTGCCACACAATAGAAACTGTTAATTCCACTTCTCTATTAACATATTGATGTAGCACAAATTTGAATTAAAAACTGTTACCT |
| | Exon10 | 73 nt | GCCTTTAGGCCATCAATCCAGCTGTAGCCAACACCATCACTGAACTGCTCTTTCTGCCTGTACAGAATGTGCT |
| | Intron10 | 47 nt | TGTTGACCATGAGAGGCAGCAATGTTTTTACATTTGATTCTGTGTGA |
| | Exon11 | 120 nt | GGGAAGAACCTCTCAAATATCATCCTGTAACAGTAGGCCTCTTTTGTAGTCGGGGTGTTGTGTGGGTAAATGAACTTCGCATTTGACATCATCTTATC<br>GGTCACATTTGATTCTGTGTGG |
| | Intron11 | 95 nt nt | TACAAGAGCAACAGATATGCTTGCATCAACATAGTTATTCCATGGAAAAGTGAGTTTTCTGTTTCATGTTCTTGCTGTTTTATTTGCTCACCTGG |
| | Exon12 | 235 nt | ACTCTCAATTGCGACACCAGGTGCCGCAACCTTGATCATCCTCGGCTTCTTGCTGGTTCCTGCCATGATGGTTGCTGGGAGATGCTCCTGCTCATA<br>GGCCGAGAGATGGACTCCAAGTGCTGCTCTCCCTGAGGGATCCAGGTTCCCCGACCACCGAGCATCCCACTCTACTGCCTTCGCCGTGCTGCATG<br>CGACGCTTGGCCCGCCTGGCACTGTCAGGATTGCCGAGTTCTGG |
| | 3' downstream sequence | 148 nt | CAGAAATCATCCGGTCTCTGATTTCATAAGAACTATAGAAACATTAATGCACAATCTTCACTCAACCTTAGGTCTGCATCACTCTAATGGCAAGACTA<br>GGTACATCTTATTTCTTATCAGAAACGGCAAGCAGGACAGGACACCATCA |
| | | | |
| ***HvASN3*** | 5' upstream sequence | | GAGCCGCCGTTCCGTTCCGCCCGTACCTTACCCCTCCCCCCCACCACCACCCGCGCCCGTCCCTCCTCGTCGGGGCGTCGCCGCCTCCGCCTCC<br>GCCTCCGCCCCGCTCGTCCGCGGCGTCTGGTCGGTCCAGCGACCGAGGCCTGAGGTCCCGCCGGCCGCCACC |
| | Exon1 (no match in genome seq) | 79 nt | ATGTGCGGCATCCTCGCCGTCCTCGGCGTCGGCGACGTCTCCCTCGCCAAGCGCTCGCGCATCATCGAGCTCTCCCGCC |
| | Exon1 | 142 nt | GATTACGGCACAGAGGCCCTGATTGGAGTGGTATACACAGCTTTGAGGACTGCTATCTTGCACACCAGCGGTTGGCTATTGTTGATCCCACATCTG<br>GGGACCAGCCATTGTACAATGAGGACAAAACAGTTATTGTGACGGT |

| | | | |
|---|---|---|---|
| | Intron1 | 249 | CAGTATCTGGAGTGAGAATGACACTTCACTGTTTCCATTTATCTTATTTCTGCTTTCATGATTCATTTCCACGTACAACACATGATTATGTCTCACCATTCTGCTTTTAGTGACTAGCATTTGCTAGTATAATTTTGTGATGGGATTTTTGAAATGTTGTGTATCGAGCTCCGCTCAATACATCTTTTGAGACACAACCTACCCACCATATTTTTGTAACTAAACTAATTATGTTTTACATTTCCAGGT |
| | Exon2 | 137 nt | GAATGGGGAGATCTATAATCATGAAGAACTGAAAGCTAAGCTGAAATCACATAAATTCCAAACTGGTAGTGATTGTGAAGTTATTGCTCACCTAGTAAGTTTGTATTCTTATTTCTATGTTCAGCTTCTCTTAGTGC |
| | Intron2 | 121 nt | TCTATTGTCTAACTTGGCTTCTGCCAATACTTTCAAATGTCTTCTCCTAAGCATCATCCTCCAGTACATATATTTTATATGTTAACATCTGTACTTCCAAATTCTATTGTTTTCACAGTAC |
| | Exon3 | 134 nt | GAGGAATATGGGGAAGAATTTGTGGATATGTTGGATGGCATGTTCTCATTTGTGCTTCTTGACACACGTGATAAAAGCTTCATAGCTGCCCGTGACGCTATTGGCATCTGTCCTTTATACATGGGCTGGGGCCT |
| | Intron3 | 927 nt | GACGGTATGCATGGAATCTGTTCTTGTAGCTACTTGTCTGTTTGATCAAAATCACAACCATGAAGTTATAGCTTTTTTCCTCTCAAATTTATCTTGGTCTGGACTTTGTGTATGGTTTGCTTGGTTATTCAACTTTTTCAACGGTCAGTCAATGTATCATGGGGAGGTGCAAAGCAGGCTGCGTTTAGGTTCATTTAAATTGAAGTTAAGATTAGAAGTAGATATATCATTGCTTGCCTACTTCATTAGAATAGAACTGGATAAGGCCAATTCATTTGCAAAAATAACAATAAAAGATAATAACCCTGGATCTATCGTCGGCTCACATCCCACAAAATCGGAGTGATATCTCTCACCATTTGCAAAGAATATAGAGGTGTTACTCCCTCTGTCTTGATTTATAAGGCGTATTGTGATTGTAAAAGTTTAATCTTTGACCATATTTTTTTATAATATTTGCTATGAGTTAAAAAATCGCAATCAAGAGAAAGAGCTTTTGTATTTGAATCTAAAGGTATAAATTCTGTGTCACATAGCTCACGTATCATGAGATTAAATTTTGGGTCAGATCTTTAGACAATCAAAATATGCCTTATATACCTGCACGAAGGAAGTATCTAGGTTGTGATGACGCCCATGATATATGTGCAACGGGAATGTCTGATGGGAAAGATGTGGATAGACATAATATTTGCCAAGCTGACTGTTAAATTTGCAATACGGAGTTAGCTATGCAAAGATTCTGAGATGTTCCGTGAGCTGTTGAAAACTATGGTTGGCTGTCTGCATTTGCAAGTGCACAGTTCTGGAACATTAGACACTGTAGTGTTTGCATGTTTCACAACTTGTTACAGTTATATGGATTTAAGTAGCTGCTACAAATCTACTATGCATTAATCTGTACATTCATTGTTATTCCAGGATCCG |
| | Exon4 | 92 nt | TTTGGTTCTCTTCGGAGATGAAGGCGTTGAGTGATGATTGCGAGCGCTTCATATCGTTCCCCCCTGGACACTTGTACTCAAGCAAAACAGGT |
| | Intron4 | 163 nt | TCAAATGTCTTGCTGTAACATATTGACGATCTCATCAGGCATTATACTAGAAATGCAGAGTTGCAAAGTCTGAAGGCAAAAATAGTATAAGTTACCATGTATGTTGCAAATATCCGTTTGTCTTGCCTACTAATCTGCTTTCTAACATTTATCCTAGGTGGCT |
| | Exon5 | 91 nt | TAAGGAGGTGGTACAACCCTCCATGGTTTTCAGAAAGCATTCCCTCAGCCCCCTATGATCCTCTTCTCATCCGAGAGAGTTTTGAGAAGGC |
| | Intron5 | 404 nt | ACGCCTTTTCTTATGCTCTGTTACATTTCATACCTTTTGATGCATAAATTGTATGTTTATAATTATTTAATGATAATCTCTTTTTTATTTTGTTTCATGTTGATATGATGTATACACGCCTCTGATTAAGTGATGCTTGTTTGGAATACCCTTGTTGATAATTCATGAGTCGTAATAGACTAATAGGTTATTGTAGCTCCCTGCAATCACATTTATGTCTTAGTGCGATGATTTCTATATTAATATCTTAGATATATGGCATTTTATTCGGAATCATGCTAATTGGTTATTAAGTATGTACGGAGTATAAATAAATTATTGTGTCTGACGCATATTTTGTAAGCTAAGTTTCTTTACGTATGTCATCTACTATATTTCTTAGATGAATTGTAAAATTCGCAGGC |
| | Exon6 | 161 nt | TGTTCTCAAGAGGCTAATGACTGATGTGCCATTTGGTGTTCTCTTGTCTGGTGGGCTTGACTCTTCTTTGGTGGCTTCTGTTGTTTCACGCCACTTGGCAGAAACAAAAGTTGCCAGGCAGTGGGGAAACAAACTGCACACCTTTTGCATTGGTTTGAAGG |
| | Intron6 | 176 nt | TACATTTCTTAATGTTGTTTACTGGTGTATCCCCATTGCTGGGAATCTTTCAAATTTAAGTTGTTTTCTTGCTCTTATGAGTGTTTTTTACAATAAAAAAGAAGGTTTTTCAGTGGAGTAACATAAGCAGAGGATAAAATATGATAGAAGTTCTAACACATTCCAATTTTTATAGG |
| | Exon7 | 81 nt | GTTCTCCTGATCTTAAAGCTGCTAAGGAAGTTGCTGACTACCTTGGCACAGTCCATCATGAATTACACTTCACAGTGCAGG |
| | Intron7 | 78 nt | TTCGTTATTTTTATTTGTCCTTCGCATAGAGGAAGAGACATTTTGAAGTTATTAAATCATCTGAAAAAAAATTGCAGG |
| | Exon8 | 221 nt | AGGGCATTGATGCTTTGGAAGAAGTTATTTATCACATTGAGACGTATGATGTAACGACCATTAGAGCAAGTACCCCAATGTTTCTAATGTCTCGGAAAATCAAATCGTTGGGTGTGAAGATGGTTCTTTCGGGAGAAGGTTCCGATGAAATATTTGGTGGTTATCTTTATTTTCACAAGGCACCAAACAAAAAGGAATTCCATGAGGAAACATGTCGGAAG |
| | Intron8 | 98 nt | GTAATTAATGATAACATTGCTCCTTCTGGTTTATATCTGCTAAGTTGTTTAGATGGTCAGCTGATTAATTTATTTGAGTTCTCTTGTCTGGCGTTTAG |
| | Exon9 | 135 nt | ATAAAGGCTCTCCATTTATATGATTGTTTGAGAGCAAACAAAGCAACTTCTGCCTGGGGTCTCGAGGCTCGTGTTCCATTCCTCGACAAAAACTTCATCAATGTAGCAATGGACCTGGATCCGGAATGTAAGATG |
| | Intron9 | 108 nt | GTATGTAATTTCTTGCTATAATACACTAATCTCACTCTAGCTCTATCCATGTATTACAATTTACAACTAGCATTCTCATCTTATATTTGTCATCTTGAAAAAAATTAG |
| | Exon10 | 81 nt | ATAAGACGTGATCTTGGCCGGATCGAGAAATGGGTTCTGCGTAATGCATTTGATGATGATGAGAAGCCCTATTTACCCAAG |
| | Intron10 | 97 nt | GTTGGAGAAGTTGATCTTTTTATGGTGTTATATTTGCTCTGTTACTTTCACAAGCTTGCGTCTATGCTCATAAGGAAGATTTTTTTTCTGTGAACAG |
| | Exon11 | 89 nt | CACATTCTTTACAGGCAAAAAGAACAGTTCAGCGATGGTGTTGGGTACAGTTGGATTGATGGATTGAAGGACCATGCTAATGCACATGT |
| | Intron11 | 122 nt | ATGCCATCTTTTCTTTGTCAAACCATTCATAACTTGACAATTGTTTACTTAGTCGACTTGCGATGGTTTCATATTACTTTACCCCCATTAGAATTAACGTTTGCTCCCATTCATGTTCAGGT |
| | Exon12 | 90 nt | GTCAGATTCCATGATGACAAACGCCAGCTTTGTTTACCCTGAAAACACACCCACAACAAAAGAAGCCTACTATTATAGGACAGTATTTGA |
| | Intron12 | 127 nt | AAAGTTTTATCCCAAGGTCCATAAGCTACAACCAGCATTTACTTTTATCATGATATTCCAAGCTATTTCATTTGCGTGGTAAAAGAATTAATGCCTGCTCTCCACTTAACTAACAATTCAATTGTGC |
| | Exon13 | 251 nt | AGAATGCTGCTAGGCTGACGGTGCCAGGAGGTCCCAGCGTTGCATGCAGCACCGCGAAAGCTGTTGAATGGGACGCCGCCTGGTCCAAGCTCCTCGACCCGTCTGGCCGTGCTGCTCTCGGTGTGCATGACGCAGCATACGAAGAGGAAAAGGCTCCCGCATCGGTCGATCCCATCTTGGAGAATGCCTTCCGTTCACCAGCCCATGGAGAGAGCACCCTAGTCAAAACCGTTGTTCCAACAGCTGCTGTT |
| | 3' downstream sequence | | TAACCTTCCACCCCATGGTTTCATATAGAATGCTCGAGAAAATGTTGTCACTTAGTTTAATTCTAGCTTCATTGCAGCTTGTCTGTAGCTTCAATCATTCAGTGTAGAAATTGCTTTCCTCTATTTTCCTTGACGCTTGCTGTTATGTTTTGCTTTCGCATGTATGTACTAAGTTTGTTTATGTGGTGAGCATTGCATTTGTAAATATTTCACCATTGTTGTGCTATCCTTGAGTTGCTCAACGGTTGGTTTTCAAACTGCAAAGTTCCATTAATAACTTCCAATGTTGGTTTCTAGATTAC |
| | | | |
| ***HvASN4*** | 5' upstream sequence | | GCGCCGCCGTAGAACGCCGTACCTCCACCACCACCCGCGTCGCCGCCGTCGCCGTCGTCGTCGCCCGTCCGTCGGGGCATCGCCGCCGCCGACGTTGCCCGTTCGTCCGCGGCGTCTGGCCACCGAGGCTTGAGGTCCCGCCGGCCGCAACC |
| | Exon1 (no match in genome seq) | 78 nt | ATGTGCGGCATCCTCGCCGTCCTCGGCGTCGGCGACGTCTCCCTCGCCAAGCGCTCGCGCATCATCGAGCTCCCCGCC |
| | Exon1 | 142 | GATTACGGCACAGAGGCCCTGATTGGAGCGGTATACACAGCTTTGAGGATTGCTACCTTGCGCACCAGAGGTTGGCTATTGTTGATCCCACATCTGGAGACCAGCCATTGTACAATGAGGACAAAACAGTTGTTGTGACGGT |
| | Intron1 | 289 nt | CTGTATCTGGAGTGAGAATAAGATTTCACCGTTTTCATCCATCTCATTGCTTTCCATGATTCATTTCAACGTGTAACACATGACTATGTCTCACTATTCTGCTTTTGGCAACTAGAATTTCTTTGTGGATAATTTTGTGATGGGATTTTATAAATGTTGTCTTCGATCTCTGCTCATTGCATCTCTGGAGACACAACCTTTAATCCACTATTTCTGTACCAATTGAGAAAATATGTTTTGTTCTTTTATTTTATTTTAACCACATCTTGTCTGTTTGACTTTTCCAGGT |
| | Exon2 | 94 nt | GAATGGTGAGATCTATAATCATGAAGAACTGAAAGCTAAGCTGAAATCTCATCAGTTCCAAACTGGTAGTGATTGTGAAGTTATTGCTCACCTA |
| | Intron2 | 152 nt | GTAAGTTTGTACTCTTATTTCTCTAATCATCGTCCTCGTGATTGTGTAGCTTCGCTTCTGCCAACACTTTTTAGGGTCTTCACCCAAGCATCGTCCTTGTGCATATATTTTATTTGTTAACATTTGTGTTTCAAACTATATTGTGTTCACAG |
| | Exon3 | 141 nt | TATGAGGAATATGGGGAAGAATTCGTGGATATGTTGGATGGCATGTTCTCTTTTGTGCTTCTTGACACACGTGATAAAACCTTCATCGCTGCACGCGATGCTATTGGCATCTGTCCTTTATACATGGGCTGGGGTCTTGAT |
| | Intron3 | 987 nt | GGTATGCACAGATTCCATTCTTATAGCTACTTTGTCCCTTCGATCAAAATCAGGAATGTGAAGTTATTATCTTTTATCTCTAAATTTATCTTGGTCTGTACTTTTGTATGGTTTCCTTGGTTATGCAACTGTTTTCAGTGGTCAATACATATAACACGACAAGGAGAAAAGCAGGTTGCATGTAGGCCTATTTAAATTGAGGTTGAGATTAGAAGTAGATATATCACTGCTTGCCTATTTCCTTAGAATAGAATTGGACAAGGCCAATTAATCTGGAAAAATAACAGTAAAAGATGATAACCCTAGAGTATAGAGGTGCTACTCCCTCTATCTGGATCCCTCTATCTGGATATTTAAGACGTATTATAATTGTCGAAGTTCAATCTTTGACCATATTTTTCCATAATATTTGTTACTGGTTACAGAATCACGATCATAGGAAGCTTTTGAATTCGACTCAAACAGTAACAATTTTGTGTCGCATAACTCACATATCGTAGGAGTAAAATCTTTAGCCAATCCAGACGAAGGTTGTGATGCCTATGATAGTGTGCAATTTGAATGTCTGATGTGAAAGATGTGGATAGACACAATATTTCCCAAGCTGACTGTTAAATTCGTTATACGGAGTTAGCTATGCCGAAGTTTCTGAGTTGTTCTGTGAATTGTCGAAACATATAGTTGGCTATTTGTATTTGCAAGTACACCCTTATGGAACAAGTAGGTACTGTAGTGTTTGTATCTGTTACAACTTGGCACAACTCTGCATTTGTGTAGTTGCTACTACTCCCTCCGTTCCTAAANNTAAGTATTTTAAGAGATTTACTAGGAGTCTACATACGGAGCAAANNNNNNNNNNNNNNNNNNNNNNNNNNNTGTCTATATACATCTGTATGTACTCTACTAATGAAATCTCTAGAAAGACTCATATTTAGGAACAGAGGGAGTAGTGTACTATGCATTAATTCGTATGTTCACTTTTATTCCA |
| | Exon4 | 99 nt | GGGTCAGTATGGTTTTCTTCAGAGATGAAGGTATTGAGTGATGATTGCGAGCGCTTCATATCGTTCCCCCCTGGACACTTGTACTCAAGCAAAACAGGT |
| | Intron4 | 165 nt | TCAAATGTCTTACTGAGTTTGTGACAAATTGACGATCTTATCAGACATTATACCAGAAATGTAGATTTGCAAACTCTGAAGGCAAAAATAGTATAAGCTACTATTAGTTGTTTCAGATATCAAATTGTCTTGCCTGGTAATTTGTTTCTAACATTTATGCTAGGT |
| | Exon5 | 94 nt | GGCCTAAGGAGGTGGTACAACCCCCCATGGTTTTCAGAAAGCATTCCCTCAACCCCTTACGATCCTCTCCTCATCCGAGAGAGTTTTGAGAAGG |
| | Intron5 | 401 nt | TCACACCTTTCCTTATGCTCTCTGTCAAATTTTTGTACCTTTTGATGCATAAAGTATACATTTATAGTTATTTCATGTTAATCTCTTCTTGATTTGCTTCATGTTGATATGATGTATTGATGCCTCTGAATAGGCGATGCTTGTATGGAATACCCTGGTTGATAATTCATGAGTACTAATAGGTTATTGCAGCTGCCTGCAATCACATTTATGTCTTAGTGCGACGATCCCTATATTGAGATCTTAGATAGATGGCATGTTATTCAAAACCATGCTAATTGGTTATTATGTATGTGAAGCGTATAAATAAGCTATTGTGTCTGACGGTATATTTCATAAGCTAAGTTTCTTTACGTATATCATCTACTACATTTTCTTAGATGAATTGCAAATTTTGCAGG |
| | Exon6 | 161 nt | CTGTTATTAAGAGGCTAATGACTGATGTGCCATTTGGCGTTCTCTTGTCTGGTGGACTTGACTCTTCCTTGGTGGCTTCTGTTGTTTCACGGCACTTGGCAGAAACAAAAGTTGCCAGGCAGTGGGGAAACAAACTGCACACCTTTTGCATCGGTTTGAAG |
| | Intron6 | 160 nt | GTACACCTCATTGCTGAGACTCACTTTAGGTCATCTTTCAAAATTTAAGTTGTTTTCTTGCTCTTATGAGTATCTTTTATAAGAAAAGGAGGGATGTTTAGTTGAGTGATACAAGCAGAGGATACAGTAGGACAAACTTAACAAGTTCCATTTTTTGTAG |
| | Exon7 | 82 nt | GGTTCTCCTGATCTTAAAGCTGCTAAGGAAGTTGCTGACTACCTTGGCACTGTCCATCATGAATTACACTTCACAGTGCAGG |
| | Intron7 | 75 nt | TTCGTTATTTTTTTCATCCTTTACATAGAGGAAGGGTCATTTTGTAGTTATTAATTCATCTGACGAAATCCCAGG |
| | Exon8 | 221 nt | AGGGTATTGATGCATTGGAAGAAGTTATTTACCACATCGAGACATACGACGTAACGACCATTAGAGCAAGTACCCCAATGTTTCTAATGTCTCGGAAAATCAAATCATTGGGTGTGAAGATGGTTCTTTCAGGAGAAGGTTCCGATGAAATATTTGGTGGTTATCTTTACTTTCATAAGGCACCAAACAAGAAGGAACTCCATGAGGAGACGTGTAGGAAG |
| | Intron8 | 99 nt | GTAATTAAAGACATAATTGCTCTTTTGGTTTATCATAATCTATATGCACAACATGGTCAGCTGATGAACTTACTAGAGTTGTTTTGCCCTGACATTTAG |
| | Exon9 | 135 nt | ATAAAAGCTCTTCATTTATATGATTGTTTGAGAGCAAACAAAGCAACTTCTGCCTGGGGTCTTGAGGCTCGTGTTCCATTCCTCGACAAAAACTTCATCAATGTAGCAATGGACCTGGATCCCGAATGTAAGATG |
| | Intron9 | 99 nt | GTATGTAATATCTTGCGATAATACACTAATTTCACTCTGGCTCTACTCATTTATTATAACCAGCATTATCATCTTATATTTATCATCTTGAAAAAATAG |
| | Exon10 | 81 nt | ATAAGACGTGATCTTGGCCGGATTGAGAAATGGGTTCTGCGTAATGCATTTGATGATAATGAGAAGCCCTATTTACCCAAG |
| | Intron10 | 86 nt | GTTGGAGAAGGCTCATCTTTTAATTGCTCTGTTACTTGTGCAAGCTTGCTTGTATCTCATAATGAATATTTATTTTCTGTGAACAG |
| | Exon11 | 89 nt | CACATTCTTTACAGGCAAAAGGAACAATTCAGCGATGGTGTTGGGTACAGTTGGATTGATGGACTGAAGGACCATGCTAGTGCACATGT |
| | Intron11 | 122 nt | ATACCATTTTTTCTTCGTTATACCATTCATAAGCTGACAGCTGTTTATTTATTTGACTTACAATGGTTTTGTATCACTTTACCCCAATCAGAATTCACAGTTTCTCCTACTCATGTTCAGGT |

| | | | |
|---|---|---|---|
| | Exon12 | 106 nt | GTCGGATTCCATGATGACGAACGCTAGCTTTGTTTACCCTGAGAACACACCCACAACTAAGGAGGCCTACTATTACAGGACTGTGTTCGAGAAATTCTATCCCAAG |
| | Intron12 | 94 nt | GTCCAGAAGCCACAACCAGCCTTTAATCTGCATCATGATATTCCAAGCTATTTCATCTGCATCGTGAATCTTAATTATCGTTCTAATTGTGCAG |
| | Exon13 | 243 nt | AATGCTGCTAGGCAAACGGTGCCAGGAGGTCCCAGCGTCGCATGCAGCACCGCTAAAGCTGTCGAATGGGATGCTGCCTGGTCCAAGCTCCTCGACCCGTCTGGCCGCGCCGCTCTTGGCGTGCATGATGCGGCGTACGAAGAAAAGGCTCCTGCATCGGTCGATCCTGTCGTGGATGACGTCTCCCGTTCACCTGCACATGACGTCAAAAGACTGAAAACCGTCGTTTCAGCAGCTGCTGTA |
| | 3’ downstream sequence | | TAACCTTCCATTCCATGGTTCTACAAATGTCGTCGTTTAGTTTAATTCTAGCTTTCCTTGCAACCTGTCTGTAGTTCCTTCAATCACGCCAGTGCGGAAATTGCTTTGCTCTACTTTCGTTCATGTTTTGCTTTCACATTTATGTACCAATTTTGTTTATGCAGTGAGCATTTTGGTTGTAAATATTTCACCGTGGTTGATATCCTTGAGTTGCTCAGCATTTGGTTTGC |
| | | | |
| ***HvASN5*** | 5’ upstream sequence | | GGCATCTCTCTCTCTCTCTCTCTCTCAACACGAGCATCATTCACTCCTCCTTCCTCTTCTTCAACCTCATCCGCCCCCCGAACCCCGCGTCGTCCGTCGCCGCGTCCACC |
| | Exon1 | 94 nt | GTGACCTGCTGCTCGGTAAAAGCCTTGAGGCCATCGATCCAGTTGTAGCCAACGCCGTCGCTGAACTGCTCCTTCTGCCTGTACAAGATATGCT |
| | Intron1 | 96 nt | GTGGACGGCAGTTGCAGACAGCATTAGTTCAGAACGCCCTGTGCATGCTACGCTGGATATCTGATCTGAAAACTCAAAGAAGGTGGTGTCTGTACC |
| | Exon2 | 81 nt | TTTGGCAGGTATGGCTCCTTCTCGTCGTCGAAAGCCTTCCTCAACACCCACTTCTCGATGCGGCCAAGATCGGCATCGTAC |
| | Intron2 | 114 nt | TGCGTCGCAGCAAACAACAAGAAAACGTCAGCAAATTCAGGGCCAAAACAAATCAGACATGCGCTTAGCACACCATTTCTCCTTTCACGGCAGAGATGAGTGAGAGCTTGTACC |
| | Exon3 | 136 nt | AGTTTCCACTCGGGGTCCATGCTCATGGCGACGTCGATGAACTCCTTGTCGAGGAACTGCACGCGGACTTCCAGCCCCCAAGCGGACGTCGCCTTGTTGGCGCGCAGGCAGTCATACTGATGAAGCGCTTTCACCT |
| | Intron3 | 111 nt | GCGGGTTTCACCACATCAGTTTCGCGTCAAACCTGAGGGAATCCGACGAGTGTGTCTTGGTTGTTTGTGCAGTATTATTAATTAGGTATGATGTAGTTTCAGTTAGCACCT |
| | Exon4 | 211 nt | TGCGGCAGGTCTCCTTGTGGAACTCCTCCTTGTTGGGGGCGAAGTGGAAGTAGAGGTAGCCGCCAAGGAGCTCGTCGGAGCCTTCCCCTGACAGCACCATCTTAACACCGAGCGCCTTGATCTTGCGCGCCATCAGGAACATCGGCGTGCTCGCACGTATCGTCGTCACGTCGTACGTCTCGTTGTGGTAGATCACCTCCTCGATGGCGTC |
| | Intron4 | 217 nt | CATCCTAAACAGGACAAAATGACTCATCACTACCTCTGAATCTCTGATCCCCCGCTGTCTGTAGTGAGCAGTCTTTTCATTTATTATATGCCGAGATGTCTTAAGGCTTAAGGTTTCAATCTCAAATTTCTGGGGTTGTCAGTGTGAGCGTTCTGCAAATTTATTATGCCAAGCATCAAGATGTCCTGAGGCTTGAGGTTTTATTTGCTTTTCTTTA |
| | Exon5 | 84 nt | CCTGGACAGTGAAATGGAACTCGTGATGGATGGTTCCCAGATAGTCAGCAACCTCTCTCGCGGCCTTCAGGTCAGGTGACCCCT |
| | Intron5 | 94 nt | ACAATGTTCAGCAACATGTTAGAATCATCAGAATTCCATGGTTCTTGAATAAGAAGAAAGAGTAGGACAAAAACATGTGAAGAAAAACAAACCT |
| | Exon6 | 84 nt | CCTGGACAGTGAAATGGAACTCGTGATGGATGGTTCCCAGATAGTCAGCAACCTCTCTCGCGGCCTTCAGGTCAGGTGACCCCT |
| | Intron6 | 217 nt | CATCCTAAACAGGACAAAATGACTCATCACTACCTCTGAATCTCTGATCCCCCGCTGTCTGTAGTGAGCAGTCTTTTCATTTATTATATGCCGAGATGTCTTAAGGCTTAAGGTTTCAATCTCAAATTTCTGGGGTTGTCAGTGTGAGCGTTCTGCAAATTTATTATGCCAAGCATCAAGATGTCCTGAGGCTTGAGGTTTTATTTGCTTTTCTTTA |
| | Exon7 | 211 nt | TGCGGCAGGTCTCCTTGTGGAACTCCTCCTTGTTGGGGGCGAAGTGGAAGTAGAGGTAGCCGCCAAGGAGCTCGTCGGAGCCTTCCCCTGACAGCACCATCTTAACACCGAGCGCCTTGATCTTGCGCGCCATCAGGAACATCGGCGTGCTCGCACGTATCGTCGTCACGTCGTACGTCTCGTTGTGGTAGATCACCTCCTCGATGGCGTC |
| | Intron7 | 111 nt | GCGGGTTTCACCACATCAGTTTCGCGTCAAACCTGAGGGAATCCGACGAGTGTGTCTTGGTTGTTTGTGCAGTATTATTAATTAGGTATGATGTAGTTTCAGTTAGCACCT |
| | Exon8 | 136 nt | AGTTTCCACTCGGGGTCCATGCTCATGGCGACGTCGATGAACTCCTTGTCGAGGAACTGCACGCGGACTTCCAGCCCCCAAGCGGACGTCGCCTTGTTGGCGCGCAGGCAGTCATACTGATGAAGCGCTTTCACCT |
| | Intron8 | 114 nt | TGCGTCGCAGCAAACAACAAGAAAACGTCAGCAAATTCAGGGCCAAAACAAATCAGACATGCGCTTAGCACACCATTTCTCCTTTCACGGCAGAGATGAGTGAGAGCTTGTACC |
| | Exon9 | 81 nt | TTTGGCAGGTATGGCTCCTTCTCGTCGTCGAAAGCCTTCCTCAACACCCACTTCTCGATGCGGCCAAGATCGGCATCGTAC |
| | Intron9 | 114 nt | TGCGTCGCAGCAAACAACAAGAAAACGTCAGCAAATTCAGGGCCAAAACAAATCAGACATGCGCTTAGCACACCATTTCTCCTTTCACGGCAGAGATGAGTGAGAGCTTGTACC |
| | Exon10 | 81 nt | TTTGGCAGGTATGGCTCCTTCTCGTCGTCGAAAGCCTTCCTCAACACCCACTTCTCGATGCGGCCAAGATCGGCATCGTAC |
| | Intron10 | 96 nt | GTGGACGGCAGTTGCAGACAGCATTAGTTCAGAACGCCCTGTGCATGCTACGCTGGATATCTGATCTGAAAACTCAAAGAAGGTGGTGTCTGTACC |
| | Exon11 | 94 nt | GTGACCTGCTGCTCGGTAAAAGCCTTGAGGCCATCGATCCAGTTGTAGCCAACGCCGTCGCTGAACTGCTCCTTCTGCCTGTACAAGATATGCT |
| | 3’ downstream sequence | | TGACGAGATGATGAAGAACGCCGCAGAGGAGTACCCGTACAACACGCCCATCAACAAGGAGGCCTACTACTACCGGATGATCTTCGAGAGGCTCTACCCTCAGGAGTCGGCGAGGGAGACGGTGCCGTGGGGTCCGAGCATCGCGTGCAGCACGCCGGCGGCCATCGAGTGGGTGGCCCAGTGGAAGGCCTCCAACGACCCCTCCGGCCGGCTCATCGCCTCCCACAACGATGCCACCCCCGCTCATGCCCACGCCAACGGCAACGGTAACGGGGCGGTGGCCAACGGGAAAGCCAACGGGAACGGCACGGCGAACGGGAACGGGAACGCCAACGGGACTCTGGTCCCGTGCTAGCCGTCGCCGCTCGCGCTAGCTGGTTCGGCCGTGGTGGGTGCGTGTGCGTGTGCGCGTGTCCTCGGACTGATGAGCCATATGCCATTGGCGCCGCGGCCGGTGCTGTTGTTGTGTACGTGTTGTAGTACGTTTCTTCAGTGTTGTTGTGTGTGCGTGTGCGTGGGATGGAGCTCGCGGTCCGAGCATGTGGCGGGGGTGATGGATCGCCTGTAGAACAAGAACGTGAATCAAGTGAACTTGTGATGTATTGATCGATCCGTCTCATATTTCCTC |

| | HvGS1_3 | OsGS1_3 | ZmGS1_2 | TaGS1b | TaGS1c | TaGS1a | HvGS1_1 | ZmGS1_4 | ZmGS1_5 | SoGS1a | OsGS1_1 | AtGS1.4 | PsGS1 | AtGS1.1 | AtGS1.2 | HvGS1_2 | LpGS1 | ZmGS1_1 | ZmGS1_3 | SbGS1 | OsGS1_2 | VvGS1 | AtGS1.3 | AtGS1.5 | |
|---|---|---|---|---|---|---|---|---|---|---|---|---|---|---|---|---|---|---|---|---|---|---|---|---|---|
| HvGS1_3 | 100.00 | 87.85 | 84.49 | 81.41 | 81.13 | 81.69 | 82.54 | 83.62 | 83.62 | 83.38 | 83.10 | 79.44 | 80.79 | 81.97 | 83.38 | 79.66 | 80.51 | 81.69 | 82.82 | 83.10 | 80.85 | 82.25 | 81.64 | 78.98 | |
| OsGS1_3 | 87.85 | 100.00 | 85.56 | 82.02 | 81.74 | 82.30 | 82.87 | 82.54 | 82.54 | 83.71 | 83.99 | 80.34 | 80.85 | 82.02 | 82.02 | 80.79 | 80.51 | 81.79 | 82.35 | 83.47 | 80.67 | 82.58 | 81.07 | 78.47 | |
| ZmGS1_2 | 84.49 | 85.56 | 100.00 | 80.85 | 80.56 | 80.85 | 81.97 | 81.92 | 81.92 | 83.10 | 83.10 | 80.00 | 80.79 | 83.10 | 81.97 | 79.60 | 80.17 | 80.06 | 81.46 | 81.74 | 80.62 | 83.10 | 81.59 | 78.92 | |
| TaGS1b | 81.41 | 82.02 | 80.85 | 100.00 | 99.72 | 99.72 | 98.31 | 90.99 | 90.99 | 91.01 | 91.57 | 84.83 | 84.23 | 84.83 | 85.11 | 80.79 | 81.64 | 82.58 | 82.58 | 84.27 | 82.30 | 85.39 | 87.01 | 80.97 | |
| TaGS1c | 81.13 | 81.74 | 80.56 | 99.72 | 100.00 | 99.44 | 98.03 | 90.70 | 90.70 | 90.73 | 91.29 | 84.55 | 83.94 | 84.55 | 84.83 | 80.51 | 81.36 | 82.30 | 82.30 | 83.99 | 82.02 | 85.11 | 86.72 | 81.25 | |
| TaGS1a | 81.69 | 82.30 | 80.85 | 99.72 | 99.44 | 100.00 | 98.03 | 91.27 | 91.27 | 91.29 | 91.29 | 84.55 | 84.51 | 84.55 | 85.39 | 81.07 | 81.92 | 82.87 | 82.87 | 84.55 | 82.58 | 85.67 | 87.29 | 81.25 | |
| HvGS1_1 | 82.54 | 82.87 | 81.97 | 98.31 | 98.03 | 98.03 | 100.00 | 91.55 | 91.55 | 92.13 | 92.70 | 85.67 | 85.07 | 85.67 | 85.39 | 80.79 | 81.92 | 83.15 | 83.15 | 84.83 | 82.87 | 86.80 | 86.72 | 80.97 | |
| ZmGS1_4 | 83.62 | 82.54 | 81.92 | 90.99 | 90.70 | 91.27 | 91.55 | 100.00 | 100.00 | 96.06 | 93.80 | 85.07 | 85.03 | 86.76 | 87.32 | 83.85 | 83.00 | 85.07 | 84.51 | 85.92 | 86.20 | 87.32 | 85.84 | 79.77 | |
| ZmGS1_5 | 83.62 | 82.54 | 81.92 | 90.99 | 90.70 | 91.27 | 91.55 | 100.00 | 100.00 | 96.06 | 93.80 | 85.07 | 85.03 | 86.76 | 87.32 | 83.85 | 83.00 | 85.07 | 84.51 | 85.92 | 86.20 | 87.32 | 85.84 | 79.77 | |
| SoGS1a | 83.38 | 83.71 | 83.10 | 91.01 | 90.73 | 91.29 | 92.13 | 96.06 | 96.06 | 100.00 | 95.22 | 86.52 | 86.48 | 87.36 | 88.20 | 85.31 | 85.03 | 85.96 | 86.24 | 87.36 | 86.52 | 89.04 | 86.72 | 80.40 | |
| OsGS1_1 | 83.10 | 83.99 | 83.10 | 91.57 | 91.29 | 91.29 | 92.70 | 93.80 | 93.80 | 95.22 | 100.00 | 86.80 | 86.20 | 87.64 | 87.64 | 84.18 | 83.05 | 85.11 | 85.11 | 86.80 | 85.67 | 88.48 | 87.01 | 80.97 | |
| AtGS1.4 | 79.44 | 80.34 | 80.00 | 84.83 | 84.55 | 84.55 | 85.67 | 85.07 | 85.07 | 86.52 | 86.80 | 100.00 | 86.20 | 88.48 | 86.52 | 84.46 | 84.75 | 83.71 | 83.71 | 84.55 | 85.39 | 85.39 | 83.33 | 80.40 | |
| PsGS1 | 80.79 | 80.85 | 80.79 | 84.23 | 83.94 | 84.51 | 85.07 | 85.03 | 85.03 | 86.48 | 86.20 | 86.20 | 100.00 | 86.48 | 86.76 | 83.00 | 84.99 | 85.63 | 85.35 | 86.48 | 82.82 | 85.07 | 83.29 | 79.77 | |
| AtGS1.1 | 81.97 | 82.02 | 83.10 | 84.83 | 84.55 | 84.55 | 85.67 | 86.76 | 86.76 | 87.36 | 87.64 | 88.48 | 86.48 | 100.00 | 92.13 | 84.18 | 85.03 | 86.52 | 85.96 | 87.36 | 85.67 | 88.20 | 86.16 | 79.55 | |
| AtGS1.2 | 83.38 | 82.02 | 81.97 | 85.11 | 84.83 | 85.39 | 85.39 | 87.32 | 87.32 | 88.20 | 87.64 | 86.52 | 86.76 | 92.13 | 100.00 | 84.46 | 85.31 | 84.27 | 84.55 | 85.96 | 86.24 | 87.08 | 84.18 | 79.55 | |
| HvGS1_2 | 79.66 | 80.79 | 79.60 | 80.79 | 80.51 | 81.07 | 80.79 | 83.85 | 83.85 | 85.31 | 84.18 | 84.46 | 83.00 | 84.18 | 84.46 | 100.00 | 94.63 | 86.72 | 87.01 | 89.27 | 89.27 | 84.46 | 81.36 | 77.84 | |
| LpGS1 | 80.51 | 80.51 | 80.17 | 81.64 | 81.36 | 81.92 | 81.92 | 83.00 | 83.00 | 85.03 | 83.05 | 84.75 | 84.99 | 85.03 | 85.31 | 94.63 | 100.00 | 88.14 | 87.29 | 89.55 | 88.70 | 85.31 | 80.79 | 77.27 | |
| ZmGS1_1 | 81.69 | 81.79 | 80.06 | 82.58 | 82.30 | 82.87 | 83.15 | 85.07 | 85.07 | 85.96 | 85.11 | 83.71 | 85.63 | 86.52 | 84.27 | 86.72 | 88.14 | 100.00 | 95.80 | 96.64 | 89.92 | 85.11 | 81.92 | 77.84 | |
| ZmGS1_3 | 82.82 | 82.35 | 81.46 | 82.58 | 82.30 | 82.87 | 83.15 | 84.51 | 84.51 | 86.24 | 85.11 | 83.71 | 85.35 | 85.96 | 84.55 | 87.01 | 87.29 | 95.80 | 100.00 | 95.52 | 89.64 | 85.39 | 81.36 | 77.56 | |
| SbGS1 | 83.10 | 83.47 | 81.74 | 84.27 | 83.99 | 84.55 | 84.83 | 85.92 | 85.92 | 87.36 | 86.80 | 84.55 | 86.48 | 87.36 | 85.96 | 89.27 | 89.55 | 96.64 | 95.52 | 100.00 | 91.04 | 86.80 | 83.62 | 78.69 | |
| OsGS1_2 | 80.85 | 80.67 | 80.62 | 82.30 | 82.02 | 82.58 | 82.87 | 86.20 | 86.20 | 86.52 | 85.67 | 85.39 | 82.82 | 85.67 | 86.24 | 89.27 | 88.70 | 89.92 | 89.64 | 91.04 | 100.00 | 85.39 | 82.49 | 77.56 | |
| VvGS1 | 82.25 | 82.58 | 83.10 | 85.39 | 85.11 | 85.67 | 86.80 | 87.32 | 87.32 | 89.04 | 88.48 | 85.39 | 85.07 | 88.20 | 87.08 | 84.46 | 85.31 | 85.11 | 85.39 | 86.80 | 85.39 | 100.00 | 85.59 | 80.11 | |
| AtGS1.3 | 81.64 | 81.07 | 81.59 | 87.01 | 86.72 | 87.29 | 86.72 | 85.84 | 85.84 | 86.72 | 87.01 | 83.33 | 83.29 | 86.16 | 84.18 | 81.36 | 80.79 | 81.92 | 81.36 | 83.62 | 82.49 | 85.59 | 100.00 | 83.24 | |
| AtGS1.5 | 78.98 | 78.47 | 78.92 | 80.97 | 81.25 | 81.25 | 80.97 | 79.77 | 79.77 | 80.40 | 80.97 | 80.40 | 79.77 | 79.55 | 79.55 | 77.84 | 77.27 | 77.84 | 77.56 | 78.69 | 77.56 | 80.11 | 83.24 | 100.00 | |
| CsGS1 | 61.08 | 59.60 | 60.97 | 63.07 | 62.78 | 63.07 | 62.50 | 61.25 | 61.25 | 61.93 | 61.36 | 60.23 | 61.54 | 61.36 | 60.80 | 60.51 | 60.23 | 61.65 | 61.65 | 62.50 | 60.51 | 60.80 | 61.93 | 59.54 | 1 |
| DmGS1 | 53.85 | 53.98 | 53.71 | 53.56 | 53.56 | 53.85 | 54.13 | 54.00 | 54.00 | 54.70 | 54.99 | 53.28 | 54.29 | 52.71 | 52.42 | 53.71 | 51.43 | 52.71 | 52.99 | 52.71 | 52.99 | 54.99 | 53.43 | 55.01 | |
| HsGS | 54.08 | 54.14 | 52.78 | 54.55 | 54.55 | 54.83 | 55.11 | 54.42 | 54.42 | 56.25 | 55.11 | 53.41 | 55.27 | 53.41 | 54.26 | 53.14 | 53.71 | 52.97 | 52.69 | 53.54 | 54.11 | 54.26 | 55.71 | 56.86 | |
| HvGS1_4 | 16.43 | 17.18 | 15.34 | 15.36 | 15.36 | 15.36 | 15.07 | 15.12 | 15.12 | 15.65 | 15.94 | 16.23 | 15.70 | 15.65 | 16.52 | 16.33 | 15.74 | 16.47 | 16.47 | 15.90 | 15.90 | 15.36 | 16.03 | 16.08 | |
| HvGS1_5 | 22.67 | 23.38 | 21.30 | 21.97 | 21.97 | 21.97 | 21.97 | 21.62 | 21.62 | 21.97 | 21.97 | 22.42 | 21.97 | 21.97 | 22.42 | 21.27 | 21.27 | 20.98 | 21.43 | 20.54 | 21.88 | 21.52 | 22.17 | 21.82 | |
| AtGS1.6 | 15.76 | 15.97 | 14.69 | 14.70 | 14.70 | 14.70 | 14.41 | 14.16 | 14.16 | 14.41 | 14.41 | 15.56 | 14.16 | 15.27 | 15.27 | 14.78 | 14.78 | 14.94 | 15.23 | 14.66 | 15.52 | 14.99 | 14.49 | 15.12 | |
| AnGSlike | 13.66 | 13.35 | 14.00 | 14.20 | 14.20 | 14.20 | 13.91 | 14.54 | 14.54 | 14.50 | 14.20 | 15.09 | 13.35 | 14.50 | 14.20 | 15.18 | 14.88 | 13.86 | 14.45 | 14.16 | 15.34 | 14.20 | 13.99 | 14.63 | |
| SfGS1 | 17.99 | 17.39 | 16.86 | 16.22 | 16.22 | 16.22 | 16.52 | 15.66 | 15.66 | 15.62 | 15.62 | 16.52 | 15.32 | 15.92 | 16.52 | 16.31 | 16.31 | 15.27 | 15.87 | 15.27 | 17.07 | 16.22 | 15.71 | 16.11 | |
| EcGS | 16.03 | 16.62 | 15.80 | 16.32 | 16.32 | 16.32 | 16.32 | 16.07 | 16.07 | 16.02 | 15.43 | 16.32 | 15.73 | 16.32 | 17.21 | 15.22 | 15.22 | 15.68 | 15.98 | 15.68 | 15.98 | 16.02 | 17.01 | 16.22 | |
| SaGS | 16.92 | 17.22 | 17.27 | 17.55 | 17.55 | 17.55 | 17.55 | 16.35 | 16.35 | 16.30 | 16.61 | 16.93 | 17.55 | 17.24 | 18.18 | 16.40 | 17.03 | 16.88 | 16.56 | 16.88 | 16.56 | 16.93 | 17.03 | 16.83 | |

## Supplementary Table 5. Percent Identity Matrix - Asparagine Synthetase proteins

| | OsASN4 | OsASN5 | HvASN5 | OsASN2 | ZmASN3 | ZmASN4 | HvASN3 | HvASN4 | ZmASN1 | OsASN3 | AtASN2 | AtASN3 | HvASN1 | ZmASN2 | HvASN2 | GmASN1 | GmASN2 | AtASN1 | Os. |
|---|---|---|---|---|---|---|---|---|---|---|---|---|---|---|---|---|---|---|---|
| OsASN4 | 100.00 | 82.62 | 11.21 | 11.32 | 11.17 | 10.99 | 11.59 | 11.42 | 12.31 | 12.69 | 12.31 | 12.34 | 12.50 | 12.27 | 11.94 | 12.13 | 11.94 | 12.50 | |
| OsASN5 | 82.62 | 100.00 | 10.31 | 10.75 | 10.61 | 10.42 | 11.57 | 11.57 | 11.95 | 12.33 | 11.55 | 11.57 | 11.76 | 11.91 | 11.57 | 11.95 | 11.95 | 12.52 | |
| HvASN5 | 11.21 | 10.31 | 100.00 | 86.11 | 83.79 | 82.32 | 69.56 | 70.34 | 70.04 | 68.99 | 71.25 | 71.88 | 72.36 | 73.05 | 70.68 | 71.10 | 70.89 | 70.68 | 1 |
| OsASN2 | 11.32 | 10.75 | 86.11 | 100.00 | 86.22 | 84.86 | 66.78 | 67.52 | 67.97 | 67.80 | 70.02 | 69.15 | 70.74 | 71.23 | 70.40 | 69.43 | 70.00 | 68.61 | 1 |
| ZmASN3 | 11.17 | 10.61 | 83.79 | 86.22 | 100.00 | 95.24 | 68.44 | 68.56 | 69.57 | 68.55 | 70.54 | 69.84 | 70.91 | 71.92 | 69.54 | 71.16 | 71.38 | 69.81 | 1 |
| ZmASN4 | 10.99 | 10.42 | 82.32 | 84.86 | 95.24 | 100.00 | 67.41 | 68.21 | 68.21 | 67.69 | 69.67 | 68.98 | 70.74 | 71.58 | 69.36 | 70.81 | 70.52 | 68.95 | 1 |
| HvASN3 | 11.59 | 11.57 | 69.56 | 66.78 | 68.44 | 67.41 | 100.00 | 95.93 | 88.12 | 91.17 | 82.35 | 83.22 | 77.07 | 75.17 | 75.95 | 77.30 | 78.47 | 75.65 | 1 |
| HvASN4 | 11.42 | 11.57 | 70.34 | 67.52 | 68.56 | 68.21 | 95.93 | 100.00 | 88.78 | 90.82 | 82.15 | 83.36 | 77.03 | 75.13 | 75.74 | 76.91 | 77.74 | 75.78 | 1 |
| ZmASN1 | 12.31 | 11.95 | 70.04 | 67.97 | 69.57 | 68.21 | 88.12 | 88.78 | 100.00 | 90.19 | 83.39 | 82.70 | 76.42 | 75.09 | 75.47 | 75.82 | 77.51 | 75.56 | 1 |
| OsASN3 | 12.69 | 12.33 | 68.99 | 67.80 | 68.55 | 67.69 | 91.17 | 90.82 | 90.19 | 100.00 | 83.39 | 84.78 | 77.45 | 74.40 | 75.30 | 76.68 | 78.55 | 76.08 | 1 |
| AtASN2 | 12.31 | 11.55 | 71.25 | 70.02 | 70.54 | 69.67 | 82.35 | 82.15 | 83.39 | 83.39 | 100.00 | 90.83 | 76.82 | 77.16 | 76.22 | 76.52 | 77.22 | 76.43 | 1 |
| AtASN3 | 12.34 | 11.57 | 71.88 | 69.15 | 69.84 | 68.98 | 83.22 | 83.36 | 82.70 | 84.78 | 90.83 | 100.00 | 77.51 | 76.30 | 75.87 | 76.87 | 78.61 | 76.78 | 1 |
| HvASN1 | 12.50 | 11.76 | 72.36 | 70.74 | 70.91 | 70.74 | 77.07 | 77.03 | 76.42 | 77.45 | 76.82 | 77.51 | 100.00 | 88.01 | 87.59 | 83.74 | 84.26 | 80.90 | 1 |
| ZmASN2 | 12.27 | 11.91 | 73.05 | 71.23 | 71.92 | 71.58 | 75.17 | 75.13 | 75.09 | 74.40 | 77.16 | 76.30 | 88.01 | 100.00 | 83.48 | 78.93 | 79.69 | 77.05 | 1 |
| HvASN2 | 11.94 | 11.57 | 70.68 | 70.40 | 69.54 | 69.36 | 75.95 | 75.74 | 75.47 | 75.30 | 76.22 | 75.87 | 87.59 | 83.48 | 100.00 | 81.28 | 81.83 | 79.35 | 1 |
| GmASN1 | 12.13 | 11.95 | 71.10 | 69.43 | 71.16 | 70.81 | 77.30 | 76.91 | 75.82 | 76.68 | 76.52 | 76.87 | 83.74 | 78.93 | 81.28 | 100.00 | 90.14 | 83.59 | 1 |
| GmASN2 | 11.94 | 11.95 | 70.89 | 70.00 | 71.38 | 70.52 | 78.47 | 77.74 | 77.51 | 78.55 | 77.22 | 78.61 | 84.26 | 79.69 | 81.83 | 90.14 | 100.00 | 83.99 | 1 |
| AtASN1 | 12.50 | 12.52 | 70.68 | 68.61 | 69.81 | 68.95 | 75.65 | 75.78 | 75.56 | 76.08 | 76.43 | 76.78 | 80.90 | 77.05 | 79.35 | 83.59 | 83.99 | 100.00 | 1 |
| OsASN1 | 8.04 | 8.38 | 12.50 | 12.95 | 12.31 | 12.31 | 12.87 | 12.89 | 12.65 | 13.86 | 13.04 | 11.85 | 12.67 | 12.41 | 13.04 | 12.52 | 12.20 | 12.31 | 10 |
